# Population size modulates the transition from gene amplification-mediated heteroresistance to stable resistance

**DOI:** 10.1038/s44259-026-00280-x

**Published:** 2026-10-09

**Authors:** Johannes Kupke, Yuwen Fang, Fereshteh Ghazisaeedi, Dennis Hanke, Frank Schreiber, Karsten Tedin, Niclas Nordholt, Marcus Fulde

**Affiliations:** 1 Department of Veterinary Medicine, Institute of Microbiology and Epizootics, Center for Infection Medicine, Freie Universität Berlin, Berlin, Germany; 2 Institute of Microbiology, University of Veterinary Medicine Hannover, Hannover, Germany; 3 Department of Materials and the Environment, Division of Biodeterioration and Reference Organisms (4.1), Federal Institute for Materials Research and Testing (BAM), Berlin, Germany; 4 Present Address: Department of Biochemistry, Charité - Universitätsmedizin Berlin, Berlin, Germany

**Keywords:** Ecology, Ecology, Evolution, Genetics, Microbiology

## Abstract

Heteroresistance is characterized by antibiotic-resistant subpopulations which can be underpinned by reversible gene duplication-amplification (GDA) of antibiotic resistance genes. It is often considered a precursor of stable resistance mutations. We investigated the evolutionary dynamics between GDA-mediated heteroresistance and stable resistance mutations in a clinical *Enterobacter cloacae* complex isolate. Exposure to ceftazidime resulted in enrichment of *ampC* β-lactamase GDAs in a concentration-dependent manner. Long-term exposure and high concentrations of ceftazidime selected for stable resistance mutations in *dacB* and *ampD*, a decrease of GDAs and a loss of population heterogeneity. Evolution experiments and mathematical modelling suggest that, in large populations, competition with GDA-carrying subpopulations can prolong fixation of stable resistance mutations in the population. By contrast, heteroresistance rescues small populations upon sudden antibiotic challenge, thereby acting as a precursor of stable resistance. This study highlights a previously underappreciated dual, population-size dependent role for GDAs in the transition from heteroresistance to stable resistance.

## Introduction

The world is facing an antibiotic crisis, with an increase of untreatable infections causing an estimated 1.27 million deaths due to antibiotic resistance in 2019^1^. Antibiotic consumption rose in human medicine by 65% between 2000 and 2015, and in veterinary medicine it is expected to rise by 67% between 2010 and 2030^2,3^. Consequently, extensive research has been conducted to elucidate the mechanisms leading to the parallel increase in bacterial resistance to antibiotics and the spread of stable, population-wide resistance. The resistance mechanisms discovered so far are diverse and include mutations leading to drug target alterations, alteration of function or expression of efflux/influx systems and the degradation or modification of drugs^4^. Generally, these mechanisms are underpinned by population-wide genetic determinants in all cells of the population that are either acquired by de novo mutations or horizontal gene transfer^4,5^. Moreover, antibiotic resistance often incurs a fitness cost to the bacterial population^6^.

However, several resistance phenomena have emerged that are of a transient nature, such as antibiotic persistence and heteroresistance^7–9^. Heteroresistance (HR) has been detected for different antibiotic classes and bacterial species^10^. HR describes the presence of resistant subpopulations that can grow under antibiotic pressure to become fully resistant populations, but which often revert back to antibiotic-susceptible populations after antibiotic withdrawal^9,10^. These transient characteristics, combined with the low frequency of these subpopulations in the absence of antibiotic pressure, make them difficult to detect using standard antimicrobial susceptibility testing^10–12^. Furthermore, HR complicates the clinical therapy of infections and has been associated with persistent bacteraemia^13^, treatment failure and increased mortality^12–18^. Prevalences for HR are not fully elucidated, due in part to the lack of standardised definitions and standard methods for the detection of HR^19^. Alarmingly, transient resistance phenomena can serve as a stepping-stone towards stable, genetically inherited resistance^20–25^. It has previously been suggested that mutations in chromosomal core genes are the predominant mechanism of HR in Gram-positive bacteria^26^. However, in Gram-negative bacteria, HR appears to occur often through gene dosage variation, notably through gene duplication-amplification (GDA)^27^. GDA formation has been described as a two-step process with an initial gene duplication event through homologous recombination between directly repeated regions (transposases, insertion sequences etc.) or by means of complex non-homologous recombination mechanisms with subsequent RecA-mediated amplification of higher frequency^28^. In antibiotic-susceptible populations, GDAs occur at relatively high frequencies compared to stable, population-wide resistance mutations, and segregate in the absence of selection through unequal crossing over events with loss of amplified regions^29^. In addition, the fitness costs associated with GDAs select against carrying excess GDAs. In the presence of antibiotic selection, GDAs rapidly become enriched even under sub-lethal antibiotic pressure, in a dynamic time- and concentration- dependent manner^30^. They lead to a very heterogeneous phenotype of resistant subpopulations^31^ and may serve as a bet-hedging strategy^32^. Interestingly, GDAs allow bacteria to adapt to toxic environmental conditions in several ways: (i) directly *via* copy number increase of a stressor-degrading enzyme (e.g. β-lactamases)^27^, (ii) indirectly via amplification of genes that compensate for fitness loss due to resistance mutations or horizontal acquisition of resistance genes^33,34^, or (iii) by increasing the probability of mutations in certain genes through copy number effects^35,36^.

Under selective conditions, such as exposure to antibiotics, GDAs are highly prevalent within bacterial populations yet remain intrinsically unstable. This combination enables them to function as an evolutionary intermediate, bridging rapid but reversible gene regulatory responses and the emergence of rare, genetically stable resistance-conferring point mutations^25,37,38^. Both stochastic and deterministic mathematical models have been used to better understand the population dynamics involved with GDAs^25,39–44^. Because GDAs arise more frequently than stable resistance mutations in heteroresistant (HR) strains, they are thought to represent evolutionary precursors of stable resistance mutations^25^. However, when stable resistance mutations emerge, they will have to compete with GDA-carrying subpopulations, which may ultimately interfere with the fixation of population-wide, stable resistance. Currently, the effect of GDA-driven resistant subpopulations on the evolutionary dynamics of resistance mutations is not well understood. Whether GDAs may attenuate the fixation of stable, population-wide resistance has not been studied. Understanding the evolutionary dynamics of GDAs and stable resistance mutations in HR populations may help to devise treatment strategies with improved outcomes for HR infections.

Here, we work with a heteroresistant, clinical *Enterobacter cloacae* complex (ECC) strain to investigate the evolutionary dynamics of GDA-carrying subpopulations and stable resistance mutations in the presence of the third-generation cephalosporine ceftazidime (CAZ). This widely used broad-spectrum cephalosporin is associated with higher resistance levels in *Enterobacter* spp. than in other *Enterobacterales* and is often mediated by ampC β-lactamases^45^. Heteroresistance in the ECC strain used in this study is based on a *recA*-dependent GDA mechanism of a 17-kilobase-pair fragment containing an ampC β-lactamase *bla*_DHA-1_ located on a single-copy IncHI/2A plasmid^31^. We conducted single-step evolution experiments to obtain information about the prevalence of GDAs and stable resistance mutations at different CAZ concentrations. To gain insights into the transition dynamics from HR to stable resistance under CAZ pressure, we performed long-term evolution experiments. We demonstrate that an early response to antibiotic pressure with GDAs is replaced by less costly resistance mutations, with a stable, population-wide resistance phenotype. Importantly, experiments informed by mathematical modelling demonstrate that, in large populations, stable resistance mutations compete with GDA-carrying subpopulations, which can delay the fixation of population-wide, stable resistance. In contrast, the ability to form GDAs can rescue small populations under antibiotic pressure. These results extend our understanding of the population dynamics of GDA-carrying subpopulations and stable resistance mutations.

## Results

### Gene duplication-amplification of ECC is reduced under high or long-term antibiotic pressure

**Fig. 1:**
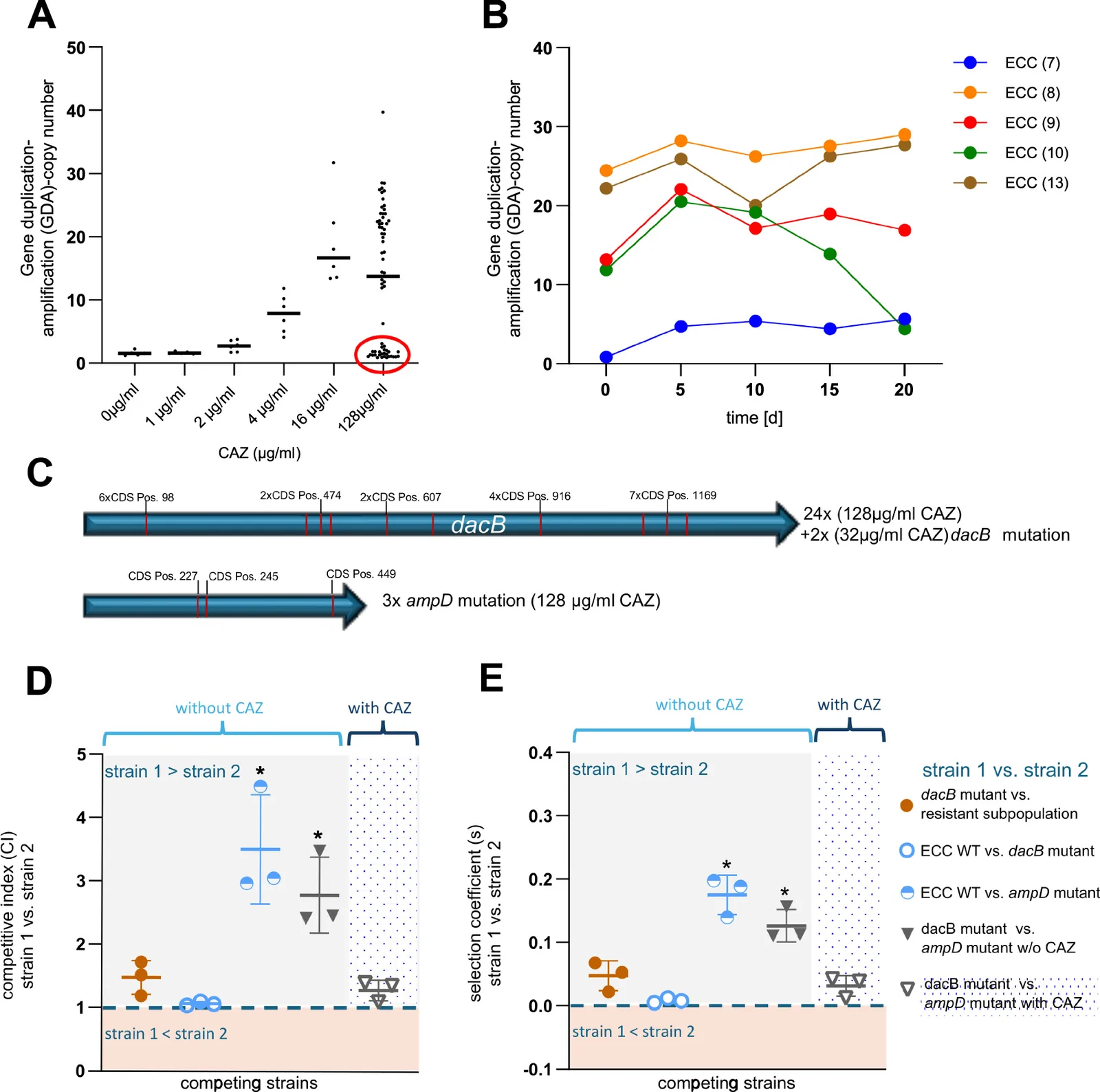
*Enterobacter cloacae* complex (ECC) evolves from gene duplication-amplification (GDA) to stable, mutation-mediated resistance. **A** ECC (WT) parental population on plates with ceftazidime (CAZ) (*x*-axis) selects for resistant subpopulations with increased gene duplication-amplifications (GDA) (*y*-axis) at increasing antibiotic exposure. Black dots represent the median GDA copy number of single colonies and horizontal black lines show the median GDA copy number of all measured colonies for the respective CAZ concentration. At 128 µg/ml CAZ, 27 of 66 colonies show strongly reduced GDA copy numbers (red circle). GDAs are determined with qPCR and the use of (g)DNA for the detection of the β-lactamase *bla*_DHA-1_. Results are displayed as a fold change value relative to the ECC (WT) parental population. **B** Long-term evolution in liquid broth with 32 µg/ml CAZ with five different ECC resistant subpopulations. A loss of GDAs is observed between 15 and 20 days of evolution in ECC 10. GDAs are detected using qPCR, representing a fold change value to the ECC (WT) parental population and the median of *n* = 3 replicates. **C** Representation of the *dacB* and *ampD* genes (blue arrows) and the positions of mutations (red vertical lines) that occurred during long-term evolution (32 µg/ml CAZ) or at high antibiotic concentrations (128 µg/ml CAZ). In the *dacB* gene, the positions of mutations that occurred repeatedly are indicated. The number of colonies which carried mutations in the respective gene and the conditions from which they were isolated are indicated to the right. **D** Results of competition assays shown with competitive indices (CI) of different strain combinations. Shown are the mean and standard deviation of competitive indices (CI) as a measure of fitness after 8 h of competition (markers indicate three independent experimental repeats). A CI > 1 indicates a fitness-advantage of strain 1 (see blue line and grey/red background). * indicates *p* < 0.05. **E** Results of competition assays shown with selection coefficients (*s*) as a measure of fitness for the same strain combinations as in **C**. Shown are mean and standard deviation of selection coefficients (*s*) of *n* = 3 independent experimental repeats indicated with symbols after 8 hours of competition. A *s* > 0 indicates a fitness-advantage of strain 1.

Previously, it has been shown how initial antibiotic pressure revealed the presence and emergence of genomic plasticity of GDA in ECC, resulting in a heteroresistant phenotype^31^. In the present study, we sought to investigate the population dynamics of the heteroresistant ECC strain in the context of the transition from unstable GDAs to stable, mutation-based resistance. To this end, we initially exposed the ECC (WT) parental population to a range of different ceftazidime (CAZ) concentrations (0, 1, 2, 4, 16, 128 µg/ml) on plates for 24 h and determined the average GDA copy numbers of CAZ-resistant colonies (Fig. 1A). GDAs were measured as fold change values relative to the ECC (WT) parental population by qPCR using genomic DNA and primers for GDA (*bla*_DHA-1_) and the house keeping gene tryptophan synthetase β-chain (*trpB*) used as a control (primer sequences are shown in Supplementary Table S1). With increasing antibiotic concentrations, the GDA copy number of the resistant ECC subpopulations increased (Fig. 1A). However, at very high antibiotic concentrations (128 µg/ml), we found that 41% (27 out of 66 colonies) of the colonies examined contained very low or no GDAs (Fig. 1A (red circle), Supplementary Tables S3.1–S3.4). The low GDA values indicated a GDA-independent resistance mechanism in these colonies. Additionally, we randomly picked five different colonies of the ECC (WT) parental population after growth on plates with 32 µg/ml CAZ (two-fold the clinical breakpoint concentration for CAZ and *Enterobacterales*^46^). These resistant subpopulations were exposed to 32 µg/ml CAZ in a 20-day long-term evolution experiment in liquid broth cultures, and the GDA copy number was determined over the course of the experiment (Fig. 1B). Within five days of evolution, the number of GDAs in four of five replicates increased to between 19 and 28 copies but decreased in one of these replicates (ECC 10) from ~14 to ~5 GDAs between 15 and 20 days (Fig. 1B). Moreover, in population ECC (7), qPCR revealed a reduced number of GDAs prior to the long-term evolution, which did not increase during the following 20 days under CAZ pressure. The GDA copy number for the remaining three replicates (ECC 8, 9, 13) fluctuated between ~ 17 and ~ 29 during the course of the experiment. The experimental setup for both evolutionary experiments is shown in the Supplementary Fig. S1. In summary, a reduction in GDA copy number occurred primarily under very high CAZ selection pressure, while it was also observed under long-term CAZ exposure. The evolved replicates were still resistant to CAZ despite reduced GDA copy numbers, which suggests the emergence of GDA-independent resistance mechanisms in ECC.

### Loss of gene duplication-amplification is due to resistance conferring SNPs with varying fitness traits

To investigate the underlying GDA-independent resistance mechanisms, we performed whole-genome sequencing of all ECC evolved replicates from the long-term evolution experiments and of 55 replicates from the single-step evolution experiment. Sequencing data revealed that all replicates with reduced GDAs had single nucleotide polymorphisms (SNPs) primarily in the carboxypeptidase *dacB* (*n* = 24/55, 43.6%) and to a lesser extent in the peptidoglycan amidase *ampD* (*n* = 3/55, 5.5%) (Fig. 1C, and Supplementary Tables S4 and S5). Both replicates with reduced GDAs from long-term evolution had SNPs in *dacB* (Fig. 1B). Replicates with high GDA did not show SNPs in the *dacB* or *ampD* genes. Mutations in *dacB or ampD* are known to result in antibiotic resistance *via ampC-*derepression and lead to the degradation of β-lactams like CAZ^47–49^. Consistent with this, RT-qPCR confirmed an upregulation of *ampC* β-lactamase gene expression in the evolved ECC *dacB* and *ampD* mutants (Supplementary Fig. S2).

Given that antibiotic resistance is frequently a fitness burden^6^, we anticipated that the population dynamics during the evolution to stable, population-wide resistance might be attributable to fitness differences. To test our hypothesis, we used competition assays in antibiotic-free media and subsequently calculated competitive indices (CI) and selection coefficients (*s*) as quantitative fitness measures (Fig. 1D, E, and Supplementary Tables S8.1 and 8.2). The *dacB* mutant (ECC_*dacB_*ev_mutant (37)) outcompeted a GDA-containing resistant subpopulation arising from the ECC (WT) parental population (Fig. 1D, E, and Supplementary Tables S8.1 and S8.2), indicating that the *dacB* mutation is less costly than carrying GDAs. Consistent with this, colony growth assays on ScanLag^50,51^ indicated that ECC SNP mutants evolved on antibiotic plates (6 *dacB* mutants & 3 *ampD* mutants out of 36 screened CFUs) accounted for fast-growing colonies (lag time < 814 min) within ECC GDA-containing resistant subpopulations (Supplementary Tables S3.1 and S3.2). Interestingly, the *dacB* mutant had only a low fitness cost relative to the ECC (WT) parental population, whereas the *ampD* mutant (ECC_*ampD*_ev_mutant (39)) was clearly out-competed by the ECC WT parental population (Fig. 1D, E, and Supplementary Tables S8.1 and S8.2), suggesting that the *ampD* mutation has substantial fitness costs. In direct competition, the *dacB* mutant outcompeted the *ampD* mutant in antibiotic-free media as well as in media with the breakpoint concentration of CAZ (16 µg/ml)^46^ (Fig. 1D, E), although the *ampD* mutant has a higher MIC than the *dacB* mutant (Supplementary Table S10, and Supplementary Fig. S10). The results of the competition assays were in good agreement with growth rate assays in liquid broth and single colony data in ScanLag (Supplementary Tables S6 and S7, and Supplementary Figs. S7.1 and S7.2). The growth defect of the *ampD* mutant was also apparent by smaller colony size distributions at 24 h in ScanLag compared to the *dacB* mutant and the ECC (WT) parental population (Supplementary Fig. S7.1 inset & Supplementary Fig. S7.2). These fitness and competition assays highlight that the higher frequency of SNPs in *dacB* over SNPs in *ampD* during the evolution from GDA- to SNP-driven resistance is likely due to the greater fitness cost associated with *ampD* mutations.

### ECC evolved SNP mutants show long-term, stable resistance and loss in heterogeneity

**Fig. 2:**
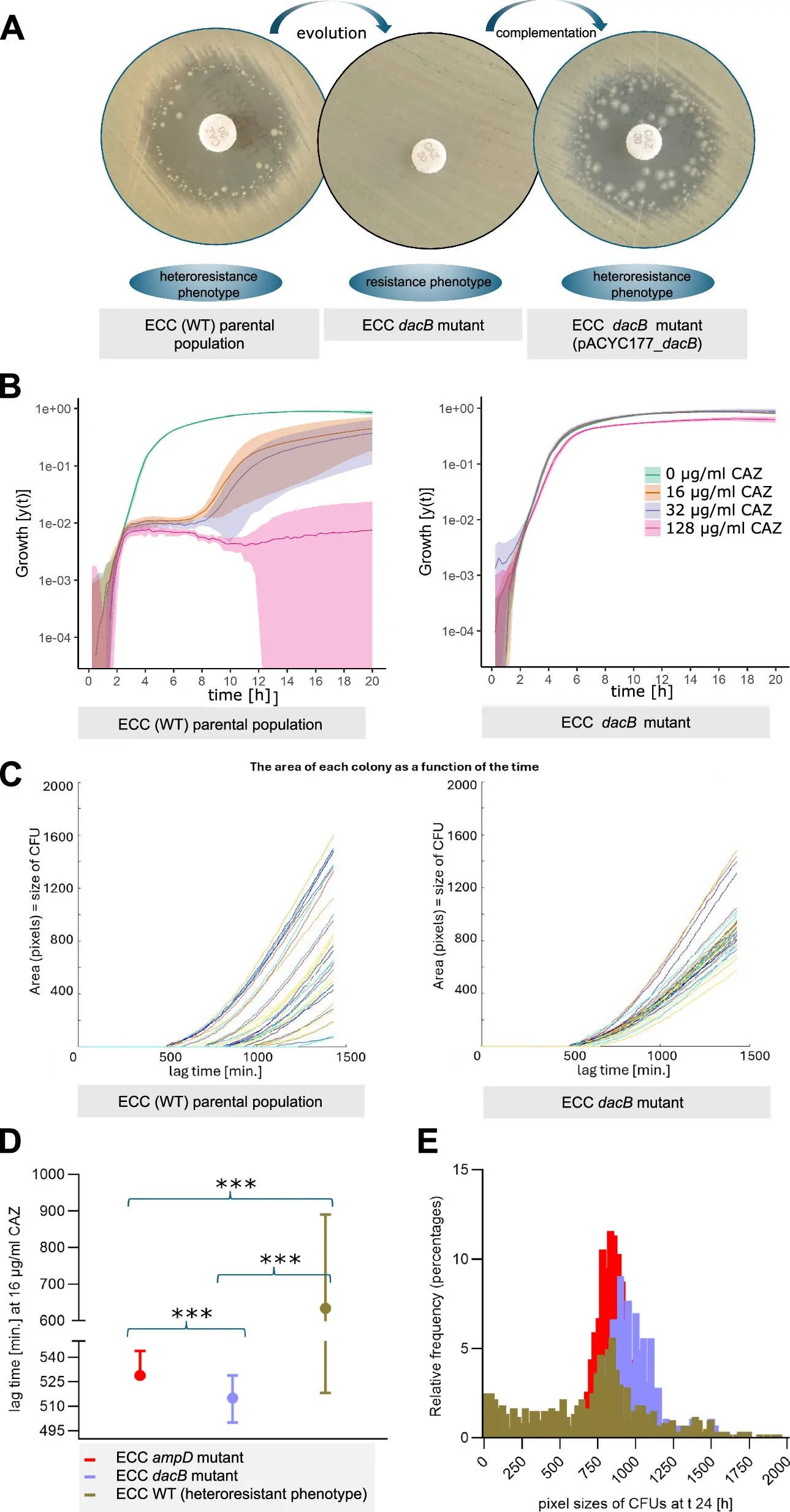
The acquisition of stable resistance is accompanied by a loss of phenotypic heterogeneity. **A** ECC (WT) parental population shows a heteroresistant phenotype (left) in agar-disk diffusion with colonies within the zone of inhibition. The ECC *dacB* mutant shows a resistant phenotype, approaching the CAZ-disk (middle). Complementation of the ECC *dacB* mutant with pACYC_*dacB* restores the heteroresistant phenotype (right) and confirms the single nucleotide polymorphism (SNP) in *dacB* as the resistance determining factor (see Supplementary Fig. S6 for identical resistance- and complementation phenotype in ECC *ampD* mutants). **B** Growth-curve data over time on the *x*-axis and OD600 on the *y*-axis and different CAZ concentrations; 0 µg/ml CAZ (green), 16 µg/ml CAZ (orange), 32 µ/ml CAZ (light blue) and 128 µg/ml CAZ (light red). The ECC (WT) parental population (heteroresistant phenotype, left panel) shows heterogeneous growth pattern for resistant subpopulations in the presence of CAZ, while the *dacB* mutant (right panel) shows homogeneous growth at all CAZ-concentrations (see Supplementary Fig. S8B for homogeneous growth of the ECC *ampD* mutant). Graphs show the result of *n* = 3 biological replicates with 3 technical replicates each. **C** The area of single colonies as a function of time for both, ECC (WT) parental population (left) and ECC *dacB* mutant with reduced GDAs on a plate with 16 µg/ml CAZ determined with the ScanLag system. The ECC (WT) parental population shows heterogeneous lag times, while colony growth of the ECC *dacB* mutant is homogeneous (see Supplementary Fig. S8A for homogeneous lag times of the evolved ECC *ampD* mutant). Each line, coloured by colony identity, represents one colony that grows over time (*x*-axis) and increases in size (Area in pixels on *y*-axis). **D** Lag times (*y*-axis) of the ECC (WT) parental population, the ECC *dacB*- and *ampD* mutant, acquired with the ScanLag system with 16 µg/ml CAZ. The median of lag times is indicated with dots; error bars show the IQR. All lag times differed significantly (*p* < 0.0001, Mann–Whitney-U-Test). **E** Distribution of colony sizes (number of pixels) show the growth defect of the ECC *ampD* mutant and wide heterogeneity in colony sizes of resistant subpopulations of the ECC (WT) parental population on plates with CAZ (time point 24 h).

Next, we determined whether the mutations in *dacB* and *ampD* affected the HR phenotype of ECC. Here, we observed a direct influence of the resistance mechanism (GDA vs. SNP) on the phenotype: evolved ECC *dacB* or *ampD* mutants displayed a ‘classical’ population-wide resistance phenotype in agar-disk diffusion (Fig. 2A, middle panel). Strikingly, complementation with wildtype *dacB* and *ampD* genes, respectively, restored the heteroresistant phenotype of the original ECC (WT) parental population (Fig. 2A, right panel, and Supplementary Figs. S3, S5, and S6). We further confirmed that SNPs in *dacB* and *ampD* led to a stable resistance phenotype as determined by subcultivation in the absence of CAZ (Supplementary Fig. S4). Targeted gene deletion mutagenesis of *ampD* and *dacB* in the parental strain confirmed the same resistance phenotype in agar-disk diffusion as the evolved ECC *dacB* and *ampD* mutants, confirming them as the causal mutations (Supplementary Fig. S3). The identical resistance phenotype observed in the knockout mutants, together with the complementation experiments, suggests that the mutations identified in the evolution experiments are likely loss-of-function mutations. Furthermore, while the growth of the ECC (WT) parental population in liquid broth under increasing CAZ was highly heterogeneous (Fig. 2B, left panel, and Supplementary Fig. S9), showing 83% reduction at 128 µg/ml CAZ compared to its original growth rate, the evolved ECC *dacB* or *ampD* mutants exhibited homogeneous growth across all tested antibiotic concentrations (Fig. 2B, right panel, and Supplementary Figs. S8 and S9). We also confirmed population heterogeneity of single colonies via ScanLag. Here, lag times of the ECC (WT) parental population in antibiotic conditions were widely heterogeneous (Fig. 2C, left panel; Fig. 2D). As we previously reported, this lag time heterogeneity is associated with different copy numbers of GDAs in resistant subpopulations^31^. We detected a median lag time of 634 min. and an interquartile range (IQR) of 518.3–890 min. In the *dacB* mutant, lag times were shorter (median of 515 min) and appeared more homogeneous (IQR of 500–529 min) than in the ECC (WT) parental population (Fig. 2C, right panel, Fig. 2D). A similar trend was observed for the evolved *ampD* mutant (Fig. 2D, and Supplementary Figs. S7.1 and S8A) as well as for the *dacB* or *ampD* mutants generated through targeted mutagenesis (Supplementary Table S9, and Supplementary Fig. S7.2). Population heterogeneity was also reflected in the distribution of colony sizes after 24 h (Fig. 2E). In conclusion, the transition from a heteroresistant phenotype via GDAs to a stable, population-wide resistance phenotype via SNPs in *dacB* or *ampD* was associated with an outstanding loss of heterogeneity.

### A mathematical model explains the population dynamics of GDA-carrying subpopulations and stable resistance mutations

**Fig. 3:**
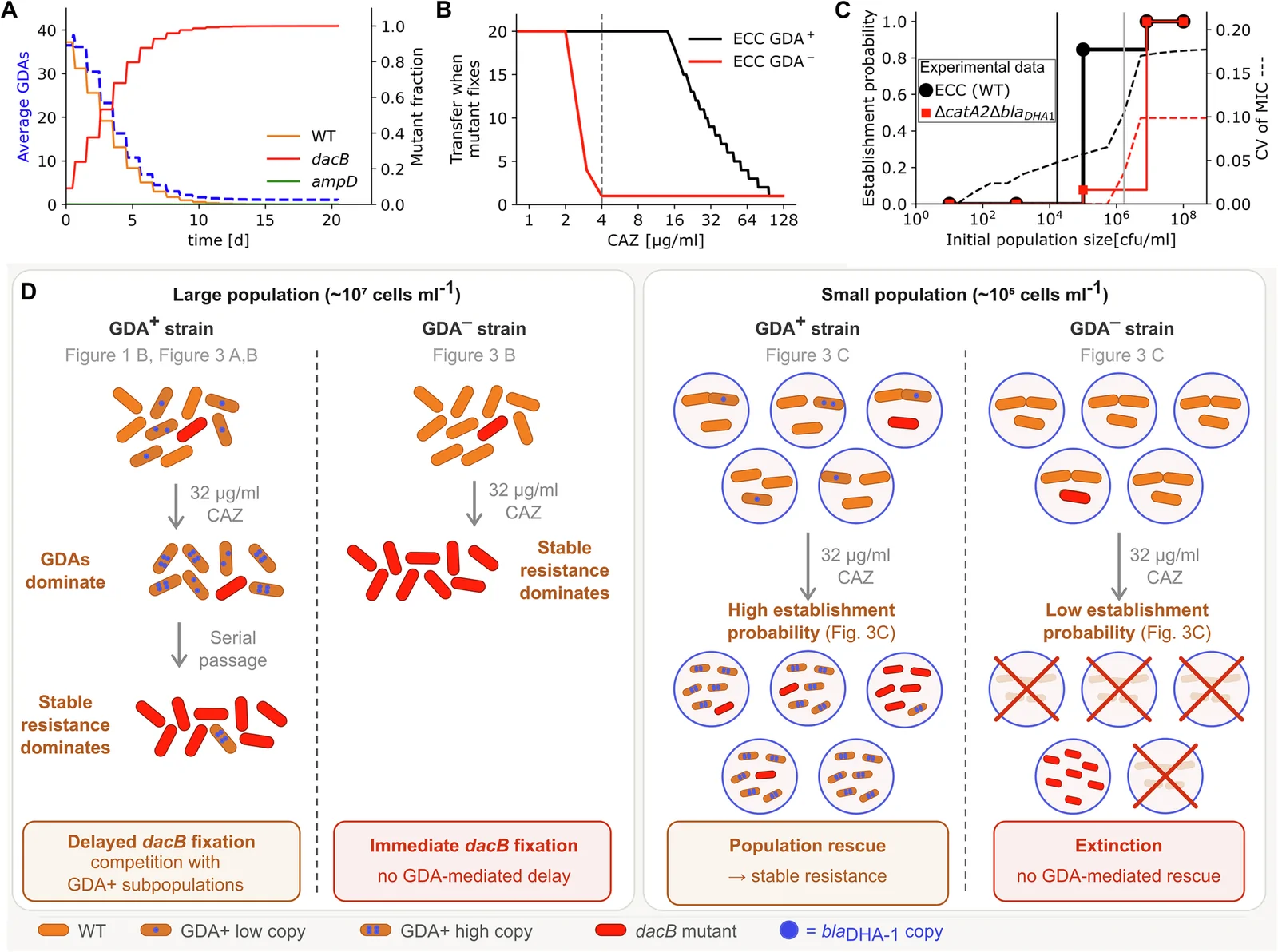
Gene duplication-amplification (GDA) delays fixation of stable resistance and rescues small populations under sudden antibiotic challenge. **A** Simulation of population dynamics under long-term exposure to 32 µg/ml CAZ, reproducing the experiment from Fig. 1B. The blue dashed line (left axis) indicates average GDA copy numbers in the ECC (WT) parental population. The right axis indicates the fraction of different genotypes: ECC WT (orange), *dacB* (red), *ampD* (green). **B** Simulation of long-term evolution at different CAZ concentrations over 20 cycles in presence (black) or absence (red) of the ability to form GDAs. Fixation timepoint of a chromosomal resistance mutation is defined as the cycle when the WT makes up less than 5% of the population. In absence of GDAs, this occurs immediately beyond the MIC of the ECC with a single GDA copy (grey dashed line), as confirmed experimentally (Supplementary Table S11.1). **C** GDA-mediated heterogeneity rescues small populations upon challenge with 32 µg/ml CAZ. Markers connected by solid lines: experimentally determined population establishment probability (left axis; data in Supplementary Table S11.2; see ‘Methods’ section for details) of the ECC (WT) population and the ECC strain unable to obtain resistance through amplification of *bla*_*DHA-1*_ (ECC ∆*catA2*∆*bla*_DHA-1_). Vertical lines indicate the model prediction of the lower bound of population size above which population growth can be established at 32 µg/ml CAZ in presence (black line, left) or absence (grey line, right) of the ability to form GDAs. Dashed lines: simulation of the coefficient of variation of the MIC (right axis) in the population carrying GDAs (black) or unable to form GDAs (red). **D** Conceptual summary of the context-dependent role of GDA-mediated heteroresistance in the evolution of stable resistance. Orange rod-shaped symbols represent bacterial cells; blue dots indicate *bla*_*DHA-1*_ copy number; red cells represent *dacB* mutants carrying stable resistance. Left: In large populations (~10⁷ cells), serial CAZ passage selects GDA-carrying subpopulations (GDA^+^ strain), which transiently dominate. Over passages, *dacB* mutants fix, but GDA^+^ subpopulations delay this via competition. In the GDA-deficient strain (GDA^–^strain), *dacB* mutants fix immediately upon challenge. Right: In small populations (~10⁵ cells), GDA^+^ cells rescue populations from extinction under CAZ, enabling subsequent emergence of stable resistance, whereas GDA^–^ populations are eliminated unless pre-existing *dacB* mutants exist.

Next, we developed a mathematical model (Fig. 3) to understand the population dynamics that govern the fixation of stable *dacB*- and *ampD*-mediated resistance in the original heteroresistant ECC (Fig. 1), and to investigate the consequences of the loss of GDA-mediated heterogeneity (Fig. 2) on population survival under sudden antibiotic challenge. To this end, we implemented a previously described mathematical model^25,39,41^ and extended it to account for different antibiotic concentrations and competition for a common, growth-limiting resource. In the model, subpopulations carrying different numbers of GDAs are constantly generated and lost through GDA^39,40^ (see ‘Materials & Methods’). Additionally, the abundance of GDAs in the population changes due to competition. Each GDA is associated with a fitness cost but increases the capability to degrade CAZ, thereby changing the MIC of the subpopulation. Evolved resistant mutants (*dacB* or *ampD*) with experimentally determined fitness costs (Fig. 1) and MIC values (Supplementary Table S11) emerge in the model at a rate set by the mutation rate observed in previous studies for *Enterobacter* spp^52^. In the *dacB* and *ampD* background, we assume that GDAs do not provide a benefit but maintain their fitness cost. The model was parameterized based on literature values and experimental data from this study (Fig. 1A, D, E) and then evaluated based on the model’s ability to reproduce the experimental observations in Fig. 1A (Supplementary Table S11, and Supplementary Figs. S11, S12, S13 and S14). We aimed to investigate why the *dacB* mutant fixed so late (cycles 15 - 20) in the long-term evolution experiment under 32 µg/ml CAZ (Fig. 1B), despite its low fitness costs as compared to GDA-carrying subpopulations (Fig. 1D, E). Hence, we simulated the population dynamics during the long-term evolution experiment in Fig. 1B. This simulation mimics the experimental setup in which a population is repeatedly grown in 32 µg/ml CAZ until stationary phase corresponding to a population size of 8 × 10^9^ CFU/ml, then diluted in fresh medium supplemented with 32 µg/ml CAZ. The simulation showed that the population is initially dominated by GDA-carrying subpopulations, resulting in a high average GDA copy number, before fixation of stable resistance in *dacB* occurs and the average GDA copy number decreases (Fig. 3A). The increase of *dacB* in the population and the concomitant decrease of GDA copies suggest that the fixation of stable resistance in heteroresistant populations is influenced by the competition between *dacB* mutants and GDA-carrying subpopulations. To further investigate this question, we simulated the long-term evolution experiment under different antibiotic concentrations and extracted the transfer cycle when either mutant, *dacB* or *ampD*, fixed at 95% in the population (Fig. 3B). The model predicted that at CAZ concentrations up to 16 µg/ml, *dacB* and *ampD* do not fix in the population within 20 transfers (Fig. 3B, black line; denoted as GDA^+^). Beyond 16 µg/ml CAZ, the number of transfers required for the mutants to fix in the population decreases as the concentration rises. The model suggests that this occurs because the average number of GDAs increases in a CAZ-dependent manner until the fitness costs of maintaining many copies become too high. Eventually, resistant mutants become less costly and increase in frequency, which results in a sharp decline in the average GDAs in the population (Supplementary Fig. S11C blue dashed line). These simulations provide an explanation for our observations in the long-term evolution experiment (Fig. 1B) that the low-cost *dacB* mutant requires multiple transfers to fix in the population, because it competes with cells harbouring GDAs. Indeed, we found that the *dacB* SNP detected at 20 days of evolution in ECC (10) was already present in a fraction (9.2%) of the WGS reads at day 15.

We next tested whether the ability to form GDAs was responsible for the observed dynamics. To this end, we omitted the ability to form GDAs from the model and repeated the simulations. Indeed, stable resistance mutations were immediately fixed in the population at CAZ concentrations exceeding the MIC of the ECC without GDAs (Fig. 3B red line; denoted as GDA^-^). To experimentally test these predictions, we repeated the evolution experiment with a *bla*_DHA-__1_ gene knockout strain (ECC Δ*catA2* Δ*bla*_DHA-1_)^31^, which is unable to acquire resistance through gene amplification of *bla*_DHA-1_. As predicted by the model (Fig. 3B), resistance that emerged in this ECC strain already after one cycle of evolution was attributable to stable resistance mutations, whereas resistance in the ECC wild-type was mostly associated with increased GDA copy number (Supplementary Table S11.1, and Fig. 1A, B). A single evolutionary cycle confirmed the model predictions of rapid mutation fixation in a GDA-deficient strain. Since the chromosomal mutation rates of the *bla*_DHA-1_ knockout strain and the parental ECC strain showed no difference (Supplementary Table S13), these results are consistent with the idea that the presence of GDA-carrying subpopulations can slow the selective sweep of stable resistance mutations through competition. These findings broaden the prevailing view of HR as a precursor of stable resistance^25^, suggesting that competition with GDA-carrying subpopulations can slow the fixation of stable resistance mutations.

### Evolutionary rescue of small populations through GDA-mediated heterogeneity

Despite the low fitness cost of the *dacB* mutant, GDAs are maintained in ECC, indicating that they provide a net benefit to ECC that offsets their fitness costs. We hypothesized that the heterogeneity generated by the GDA mechanism provides a net benefit to ECC by preparing the population for adverse conditions such as sudden antibiotic challenge. Heterogeneity is often considered to be a bet-hedging strategy to ensure the survival of a population under uncertain conditions without compromising overall population fitness^53,54^. Decreasing population size also decreases the chance that rare genotypes will be present in small populations. To test whether GDA-mediated heterogeneity (Fig. 2) can sustain the survival of the ECC population under conditions such as a sudden challenge with high concentrations of antibiotic, we performed model simulations and experiments in which we challenged the ECC and the ECC which cannot obtain resistance through amplification of *bla*_DHA-1_ (ECC Δ*catA2*Δ *bla*_DHA-1_) with 32 µg/ml CAZ, starting from different population sizes and recorded the establishment probability, i.e. the fraction of cultures at a starting population size that was able to establish growth (Fig. 3C, black and red markers; Supplementary Table S11.2). As predicted by the model, our experiments showed that heteroresistant ECC populations were able to establish growth with high probability (above 0.8), starting from as few as 1 × 10^5^ cells per ml (Fig. 3C, black markers), whereas the probability of ECC Δ*catA2*Δ *bla*_DHA-1_ to establish growth below 8 × 10^6^ cells per ml in the initial population was below 0.1 (Fig. 3C, red markers). The experimental findings are in line with the model predictions for the lower bounds of population establishment (Fig. 3C, vertical black and gray lines). Because ECC Δ*catA2*Δ *bla*_DHA-1_ cannot obtain resistance through amplification of *bla*_DHA-1_, the strain relies on rare resistance mutations. Such mutations are unlikely to be present in small populations, i.e. with a mutation rate for cephalosporine resistance of approximately 2 × 10^−8^ ^52^. In contrast, the heteroresistant population is rescued by the ability to form GDAs, which occur at higher frequencies and introduce heterogeneity into the population. The rate for the initial duplication event has been found to be orders of magnitude higher, in the range of 10^−2^ to ~10^−4^ ^40,55^. From the model, we calculate the coefficient of variation (CV) in the MIC of the subpopulations (Eq. (2)) prior to CAZ challenge as a measure for population heterogeneity and find that the failure to establish growth in small populations is accompanied by a decrease in heterogeneity (Fig. 3C, dashed lines), which is caused by the absence of subpopulations with high GDA copy numbers. Therefore, our experimental and theoretical results provide evidence that GDA-mediated heterogeneity can rescue small populations in uncertain conditions, which resembles aspects of a bet-hedging-like strategy.

## Discussion

In this study, we investigated the evolutionary transition from heteroresistance (HR) to stable population-wide resistance using a clinical *Enterobacter cloacae* complex (ECC) isolate exhibiting ceftazidime (CAZ) HR. We demonstrated that GDA-driven resistant subpopulations influence this transition in two fundamentally different ways: Firstly, rather than generally facilitating the evolution of stable resistance, our results and model simulations suggest that resistant subpopulations carrying GDAs compete with stable resistance mutations, slowing their fixation in large populations. Secondly, GDAs can rescue small populations from extinction, thereby facilitating the emergence of stable resistance through subsequent target gene mutations, which then replace the HR phenotype in the long term. Together, these findings broaden the prevailing view of HR as a precursor of stable resistance, suggesting a context-dependent dual role in which GDAs act as precursors of stable resistance in small populations, but may delay the fixation of stable resistance in large populations (Fig. 3D).

In the ECC strain investigated, GDA-mediated HR evolved into stable resistance through the acquisition of low fitness-cost mutations in *dacB* and *ampD* during antibiotic selection. Whereas resistance in GDA-containing subpopulations initially resulted from transient amplification of *bla*_DHA-1_, prolonged or high CAZ concentration selection favoured mutations that derepressed *ampC* expression, resulting in stable β-lactamase overproduction independent of gene amplification. Consequently, the initially heterogeneous, transient resistance phenotype was replaced by genetically stable, population-wide resistance. To our knowledge, this study is the first to demonstrate the evolutionary transition from GDA-mediated heteroresistance to stable resistance driven by *dacB* and *ampD* mutations in *Enterobacter cloacae* complex. Notably, *ampC*-derepression via SNPs is considerably more prevalent in *Enterobacter* than in other *Enterobacterales*^52^, underscoring the relevance of this resistance mechanism in this genus. DacB and AmpD are involved in cell wall recycling and metabolism. Their loss of function leads to a constant *ampC* β-lactamases upregulation through metabolic feedback^47–49^. While *ampC*-derepression in *Enterobacter cloacae* has been mainly assigned to SNPs in *ampD*^48,56–59^, *dacB* SNPs confer resistance in *Pseudomonas aeruginosa*^48,60^. One possible reason for the prevalence of *dacB* SNPs in this ECC is the observed fitness loss and growth defect of *ampD* mutants shown (Fig. 1D, E).

The fitness cost associated with a resistance mechanism can determine the development, stability and reversibility of resistance^61^. GDA is a costly resistance mechanism compared to a *dacB* mutation (Fig. 1D&E). Three mechanisms have recently been proposed that reduce GDA-associated fitness costs under prolonged antibiotic stress^25^: gene silencing^62^, replacement of the GDA copy number-based resistance with other mutations, and restructuring of the amplified gene fragment to a minimal length containing only the resistance target gene^63^. In our study, mutations served as the long-term strategy to reduce fitness costs in the heteroresistant ECC strain. Replacement of GDAs by rare gene mutations was also shown for *Salmonella* Typhimurium, *Klebsiella pneumoniae* and *E. coli*^25,41^. Recently, Pal and Andersson observed similar transitions from GDA-mediated HR to less costly, stable resistance, which they attributed to compensatory mutations^25^. From the fact that an increase in GDAs precedes the occurrence of stable resistance mutations, they conclude that GDAs act as precursors that facilitate the evolution of stable resistance. Our results build on these findings, suggesting that the role of GDAs in this transition may be more nuanced than a uniform facilitation, and that their effect on the fixation of stable resistance is shaped by population size: stable resistance mutations are in competition with GDA-carrying subpopulations in large populations, which may slow the fixation of *dacB* SNPs in comparison to a knock-out strain that is unable to acquire resistance through GDAs (Figs. 1B and 3B). In contrast, the comparably high frequency with which GDAs are generated introduces heterogeneity and may rescue small populations (Fig. 3D) and hence, enable the evolution of stable resistance once population size becomes large. The central conclusion regarding population size was drawn in a similar manner by Schenk et al.^64^. They showed that population size determines the type of mutations in the population. In small populations, mutations with a high rate and lower impact on resistance were more likely to occur, while in large populations, they observed a shift towards low-rate but high-impact mutations and thus a high degree of resistance. If we relate these observations to our results, the frequently occurring GDAs can be considered high-rate, low-impact mutations, and SNPs in *dacB* and *ampD* can be considered low-rate but high-impact mutations that ultimately fix in the population. In fact, SNPs in *dacB* and *ampD* showed higher resistance than the WT with the ability to form GDA (Supplementary Table S10). Importantly, both resistance mechanisms can provide resistance to CAZ as defined by the clinical breakpoint (16 µg/ml)^46^.

Modelling of GDA- or SNP-probability has been previously reported^25,39,41^. We extended these prior models to account for different environments, i.e. CAZ concentrations, competition for a common resource to allow selective sweeps, and incorporated it into a flexible framework that allows simulation of experimental and environmental conditions. The model predicted two qualitative properties of the population dynamics during resistance evolution in the presence of GDAs: (i) fixation of stable resistance is slowed down and (ii) small populations are rescued under sudden antibiotic stress. In alignment with Pal and Andersson^25^, model simulations suggested that population dynamics are initially dominated by GDA subpopulations, whereas SNPs quickly take over the population at high CAZ concentrations, when GDAs become too costly due to their high copy number (Supplementary Fig. S11C). Consistent with our evolution experiment (Fig. 1B), the model suggests that GDAs can slow the fixation of mutants due to competition (Fig. 3B). The difference in the time to fixation of SNPs (Fig. 3) is not explained by differences in mutation rates, as the chromosomal mutation rate is independent of the ability to form GDAs (Supplementary Table S13), indicating that the observed dynamics can be attributed to competition with GDA-carrying subpopulations. The qualitative model predictions, i.e. the delay of SNP fixation through competition with GDA subpopulations, are largely independent of the parameter choice or the form of the cost- and benefit function (Supplementary Figs. S12–14). However, the quantitative agreement between experimental observations and model predictions depends on the parameter choice and on the equations that describe the relation between CAZ concentration and fitness, GDA copy number and CAZ degradation (Supplementary Figs. S12–S14). Here, we assume constant costs and benefits per GDA, with benefits, i.e. resistance, scaling linearly^65–67^ and costs multiplicatively with copy number^25,39,41^, consistent with previous studies; in bacterial populations, both relationships may vary non-linearly with copy number or antibiotic concentration. In this ECC, resistance levels increase linearly with copy number, as most recently shown for the range of 2 to 12 GDAs^67^. The extent to which evolutionary trajectories are shaped by population dynamics between alternative resistance mechanisms may depend on epistatic interactions of mutations^68^, which create complex fitness landscapes^69^. We further assumed no epistatic interactions between GDAs and chromosomal mutations; the extent to which such interactions shape competition dynamics between alternative resistance mechanisms needs further investigation.

HR can be considered to increase population plasticity, which prepares populations for environmental fluctuations, such as sudden antibiotic stress. Lalejini et al. show with digital evolutionary simulations comparing plastic and non-plastic populations that non-plastic populations, somewhat comparable to our GDA-deficient knockout strain, were more frequently subject to selective sweeps, i.e. mutations that quickly become established in the population and replace other beneficial clones^70^. The frequency of GDA and the associated heterogeneity of copy numbers in a heteroresistant strain may rescue the population under uncertain conditions such as sudden high antibiotic pressure, when population size is small (Fig. 3D), and thereby facilitate the emergence of stable resistance at later times.

The clinical implications of GDA-mediated HR differ fundamentally from those of a stable, population-wide resistance. In contrast to stable mutations, GDA-mediated HR is copy-number dependent, reversible and characterized by phenotypic plasticity. As a result, heteroresistant populations frequently evade detection by routine antimicrobial susceptibility testing, leading to treatment decisions being made without recognition of resistant subpopulations^12,18,30^. Understanding the transition to stable resistance is thus of direct relevance for both antimicrobial therapy and surveillance strategies.

In summary, our findings identify GDA-mediated HR as a dynamic evolutionary intermediate that shapes, rather than simply precedes, the emergence of stable antibiotic resistance. By combining experimental evolution, mathematical modelling, and functional validation, we demonstrate that the evolutionary consequences of GDAs depend on population context. In large populations, GDA-containing subpopulations compete with genetically stable resistant clones and thereby delay their fixation, whereas in small populations GDAs promote survival under antibiotic stress, creating the opportunity for stable resistance mutations to arise and eventually dominate. More broadly, our work provides a conceptual framework for understanding how reversible gene amplification and stable genetic adaptation interact during bacterial evolution and highlights the importance of considering population dynamics when predicting the emergence of clinically relevant antibiotic resistance.

## Methods

### Strains, media and antibiotics

The strain used in this study, IMT 49658-1, is a clinical isolate of an *Enterobacter cloacae* complex (ECC), *Enterobacter hormaechei* strain, which was isolated from the wound of a horse in the equine clinic of the department of Veterinary Medicine at the Freie Universität Berlin (NCBI bioproject number: PRJNA1020684, biosample number: SAMN37527804, accession numbers CP135270-CP135274). Details on bacterial identification to species level can be found in Kupke et al.^31^ In agar disk diffusion antibiotic resistance assays with ceftazidime (CAZ), the strain exhibits a heteroresistant phenotype with CAZ-resistant colonies within a large inhibition zone. The subpopulations of resistant colonies show a reversible CAZ-resistance phenotype, due to a GDA of a plasmid-derived, *ampR*_DHA_-*bla*_DHA_ β-lactamase module^31^. In this study, we define the strain as the ECC (WT) parental population.

In evolution experiments (see below), we isolated spontaneous *dacB* and *ampD* mutants from CAZ-resistant subpopulations. Antibiotic resistance tests (agar disk diffusion, MIC assays), evolution experiments, growth curves, ScanLag experiments, and fluctuation assays were all performed using cation-adjusted Mueller-Hinton II (MH II) agar and broth (Becton Dickinson and Company, Sparks, USA) as the growth media. LB broth (Lennox formulation; Carl Roth, Karlsruhe, Germany) was used as the growth medium for competition experiments and for the growth of strains for transformation and generation of competent cells for mutagenesis experiments. In the latter, we also used SOC-Medium. Experimental cultures were incubated at 37 °C, with the exception of ScanLag experiments, where the plates were incubated at 33 °C. Ceftazidime (Sigma-Aldrich, St. Louis, USA) was used at the concentrations indicated in the text and figure legends. Nalidixic acid sodium salt (Sigma-Aldrich, St. Louis, USA) was used in fluctuation assays. Antibiotic stock solutions were stored at −20 °C, prepared and aliquoted at 10 mg/ml until use. Prior to MIC assays, working solutions were prepared by dilution of the frozen stock solutions immediately before use.

Experiments were generally performed using three biological replicates with three technical replicates each. For MIC and competition assays of evolved ECC *dacB*/*ampD* mutants, one biological mutant clone per mutant variant was analysed on three independent days. Long-term evolution in 32 µg/ml CAZ liquid broth was carried out with 5 biological replicates.

### Generation of gene deletion strains in *Enterobacter cloacae* complex (ECC)

Deletion of the *dacB* and *ampD* genes was conducted as described by Kupke et al.^31^ using the λ Red recombinase gene deletion/replacement protocol as previously described^71^, with minor modifications. Briefly, a derivative of plasmid pKD4, pKD4ble, in which the kanamycin resistance was replaced with a bleomycin/phleomycin resistance *ble* gene cassette^31^, was used as a template for the generation of the mutagenic PCR products containing the *ble* gene flanked with homologous regions of the target gene to be deleted. The mutagenic PCR products were purified using the QIAquick PCR purification Kit from Qiagen and introduced by electroporation into electrocompetent cells of *Enterobacter cloacae* (ECC) (WT) IMT 49658-1 containing plasmid pSIM18^72^ encoding a heat-inducible λ Red recombinase and hygromycin-resistance selection, as previously described^73^. The mutants were then selected after 24–48 h incubation on plates containing 15 µg/ml phleomycin. Gene/sequence deletions were confirmed by PCR and sequencing, followed by the preparation of electrocompetent cells from the potential mutants. To eliminate the antibiotic-resistance cassettes, plasmid pCP20^74^ encoding an FLP recombinase was introduced, followed by subsequent screening for loss of bleomycin resistance and PCR verification of loss of the bleomycin resistance cassette. Subsequently, clones that had lost bleomycin resistance were cured of pCP20 by growth at 37 °C, and screened for loss of plasmid-derived chloramphenicol resistance at 30 °C, as previously described^74^. As noted in Kupke et al.^31^, all gene deletions were performed in a Δ*catA2* strain background to allow chloramphenicol selection and screening for plasmid pCP20. Supplementary Table S1 lists all primers used for the generation of *dacB* and *ampD* knockout strains.

### Agar-disk diffusion assays

Agar disk diffusion method was performed according to CLSI VET01 (performance standards for Antimicrobial Disk and Dilution Susceptibility Tests for Bacteria isolated from Animals) with ceftazidime (CAZ; Becton Dickinson BBL^TM^ SensiDiscTM, Sparks, USA) for ECC (WT) parental population IMT49658-1, IMT49658-1 Δ*catA2* Δ*dacB*, IMT 49658-1 Δc*atA2* Δ*ampD* and for the *dacB*_ev and *ampD*_ev mutant strains generated in the evolution experiment as well as for the corresponding pACYC_*ampD*- and pACYC_*dacB*-complemented strains. Briefly, a bacterial density of McFarland standard 0.5 determined in NaCl was incubated on Mueller-Hinton II (MH II) plates for 16–20 h at 35 °C and subsequently the phenotype (inhibition zone size and colonies in the inhibition zone) was assessed.

### Evolution experiment with ECC IMT 49658

Five different colonies/replicates of the ECC (WT) parental population IMT 49658 grown for 24 h on 32 µg/ml ceftazidime (CAZ) plates in the ScanLag apparatus were randomly harvested and grown for 20 days at 37 °C and 200 rpm in 10 ml MH (Mueller Hinton) II broth containing 32 µg/ml CAZ. After each overnight growth (24 h), 100 µl of bacterial suspension was transferred to fresh 10 ml MH broth containing 32 µg/ml CAZ and incubated further. At time points 5, 10, 15 and 20 days, aliquots of the bacterial cultures were centrifuged, and the resulting pellets containing approximately 1 × 10^9^ cells were stored at −20 °C for DNA extraction and subsequent sequencing and SNP analysis as well as GDA copy number detection with qPCR. An additional aliquot of each culture was stored at −80 °C in ~20% glycerol (final concentration). In a second approach, the ECC IMT 49658 was spread uniformly on MH II agar plates with 0, 1, 2, 4, 16 and 128 µg/ml CAZ at a density of 1 × 10^7^ cells and incubated at 37 °C for 24 h. The colonies were then harvested and processed for DNA extraction, SNP analysis and GDA copy number detection as described. A schematic overview of the evolution experiments is shown in Supplementary Fig. S1. GDA copy numbers in Fig. 1A at lower concentrations (≤16 µg/ml CAZ) were used for illustration purposes only.

### Growth rate assays

Bacterial growth rates were determined using bacterial suspensions of 1 × 10^6^ CFU/ml in MH II broth prepared from strains grown overnight on MH II plates. From each bacterial suspension, 100 µl was transferred to wells of a 96-well, non-treated, polystyrene plate with flat bottom wells (Costar/Corning 3370; Corning incorporated, 2 Alfred Road, Kennebunk, ME 04043 USA), resulting in a starting inoculum of 1 × 10^5^ cells. For each strain, three technical replicates were performed for growth in MH II broth containing 0, 16, 32 or 128 µg/ml CAZ, respectively. Bacterial growth was determined by optical density measurements recorded every 15 min at 600 nm (OD600) and 450 nm using a BioTek Agilent Synergy HTX Multimode Plate Reader (BioTek). Bacterial growth was determined for 20 h at 37 °C with intermittent shaking. The growth curve determinations were performed independently, three times, including growth medium controls of MH II broth in triplicate to ensure no cross-contamination. The maximum growth rates of the ECC (WT) parental population IMT 49658-1 and of evolved ECC *dacB*- and *ampD*_ev_mutants as well as the respective knockout strains were calculated using the 600 nm data from growth without CAZ and the quantitative growth curve evaluation software QurvE (Version 1.1) [Wirth, N.T., Funk, J., Donati, S. et al. QurvE: user-friendly software for the analysis of biological growth and fluorescence data. Nat Protoc (2023). https://doi.org/10.1038/s41596-023-00850-7]. The relative growth rate was calculated with the formula: max. growthrate mutant / max growthrate ECC WT IMT 49658-1.

### ScanLag growth analysis of *dacB* and *ampD* mutants of the heteroresistant ECC strain IMT 49658

To determine the colony growth of individual colonies over time, ScanLag experiments were performed as described by Levin-Reisman et al.^50,51^. The time of colony appearance (colony appearance time **≙** lag time) is defined as the time at which a colony reaches the detection threshold area of 10 pixels. Growth times are defined as the time required for a colony to increase its area from 15 to 45 pixels.

To compare the ECC *dacB_*ev and *ampD_*ev mutants and the corresponding knockout strains with the ECC (WT) parental population IMT 49658-1, the experiment was performed on MH II plates with or without 16 µg/ml CAZ (1x breakpoint concentration of CAZ for *Enterobacterales*^46^ (Table 2A from ref.^46^). Only resistant subpopulations of the original heteroresistant ECC (WT) parental population can grow on CAZ plates, as shown in a previous study^31^.

Briefly, strains were grown overnight on MH II plates and the following day, single colonies were inoculated and grown to exponential phase (OD_600_ 0.5) in 10 ml MH II broth at 37 °C with aeration (shaking). To detect single colonies in ScanLag, a dilution of the culture to 10^3^ CFU/ml was performed and 100 µl of the resulting bacterial suspension was plated uniformly on MH II plates for experiments without CAZ and for *dacB*- and *ampD*_ev_mutants or the corresponding knockout strains with CAZ. For the ECC (WT) parental population IMT 49658-1, 100 µl of bacterial suspensions at 1 × 10^7^ CFU/ml were required to measure resistant subpopulations on CAZ plates. To achieve optimal contrast and moisture absorption, a sterile black felt cloth was placed between the plates and the lids of the plates as described in ref.^51^. The plates were then incubated for 24 h at 33 °C (upper limit of temperature of the scanners) on the plateholders of the scanners (Epson Perfection V370 photo). Image acquisition was controlled by the custom software UnixScanningManager (https://github.com/nirda/UnixScanningManager, commit 5d94cd3) on a Linux operating system (Ubuntu 20.04 LTS AMD 64) with an initial waiting time of 240 min and a scan of the plates every 15 min. The resulting image data were analysed using MATLAB R2020a (MATLAB, 2020) version 9.8.0.1396136 (R2020a). Natick, Massachusetts: The MathWorks Inc.) with a custom script containing the previously released analysis image software NQBMatlab(https://github.com/oferfrid/NQBMatlab/tree/V16, commit 65306ce)^51^. After removal of segmentation artifacts in the curated image analysis, results were exported to Microsoft Excel and analysed for appearance and growth time^50,51^ information per colony. Plots of the data were generated using GraphPad Prism version 8.4.3 (686) for Windows, GraphPad Software, San Diego, California USA, www.graphpad.com (Fig. 2D, and Supplementary Fig. S7.1). Pixel-size data (Fig. 2E, and Supplementary Figs. S7.1 inset & S7.2) was visualised with GraphPad Prism after being extracted from the P.data.mat file created with the image software NQBMatlab and deletion of merged colonies and artifacts. Figure 2C and Supplementary Fig. S8 originate from the plate view in the ScanLag App of the image software NQBMatlab.

### Competition assays

Competition assays comparing the growth of the wildtype and mutant strains in mixed cultures were conducted as described by Kupke et al.^31^. Briefly, different combinations of strains were used to inoculate batch cultures at the same initial concentrations (CFU/ml) for each strain, consisting of an equal inoculum of 200 µl of 2 × 10^6^ CFU/ml in 6 ml Luria Bertani (LB Lennox) broth per strain, and incubated at 37 °C with shaking. We compared the ECC (WT) parental population IMT 49658-1 vs. ECC *dacB*_ev_mutant (37) -and *ampD*_ev_mutant (39), the ECC *dacB*_ev_mutant (37) vs. ECC *ampD*_ev_mutant (39) and the ECC *dacB*_ev_mutant (37) vs. ECC IMT49658 resistant subpopulation with GDAs. For the competition between ECC *dacB*- and *ampD*_ev_mutant, we additionally performed a competition in liquid broth with 16 µg/ml CAZ. To distinguish the two strains, we used a neomycin-cassette containing plasmid pProbe´-gfp (LAA)^75^ (Addgene Cat. Nr. 40171) harbouring the red fluorescence protein mScarlet under transcriptional control of the constitutive pnptII promoter, as previously described^31^. At time points 0, 8, 24 and 48 h post-inoculation, pellets were prepared from aliquots of the bacterial suspension by centrifugation followed by resuspension of the cells in PBS. The bacterial suspensions were diluted in 1× PBS and spread in a volume of 100 µl onto plates (triplicates) either with or without neomycin. The strains could therefore be distinguished by the growth on neomycin and by colour of the colony (red). To control for a possible influence of the plasmid on growth, two competition assays were performed simultaneously, one with pProbe-gfp (LAA)+mScarlet in one strain and a second one with the same plasmid in the corresponding competing strain. In parallel, two control assays were performed with each of the competing strains alone and containing the plasmid to determine plasmid loss over the course of the experiment in non-selective media. Finally, competition was calculated by counting colonies (CFU) for the time points 0, 8, 24 and 48 h after the plates had been incubated for 24 h. The number of CFU on plates with and without neomycin was used to calculate the competitive index (CIs) using the following formula: CI = (strain 1 output/strain 2 output)/(strain 1 input/strain 2 input). Selection coefficients (*s*) were calculated from the data at time point 8 h: S = (ln(strain 1 output/strain 1 input) – ln(strain 2 output/strain 2 input))/8 h.

### Complementation experiments

Complementation of the ECC Δ*dacB* or Δ*ampD* loss-of-function mutants, as well as of the *dacB* or *ampD*_ev_mutants evolved under CAZ pressure, was performed by introduction of the intact *dacB* or *ampD* genes into the *ECC ΔcatA2 ΔdacB* or ΔcatA2 Δ*ampD* knockout strains, respectively. For this purpose, an X*ba*I restriction site was first introduced into the low copy number plasmid pACYC177^76^ by performing an inverse PCR reaction using the primers 177XbaIF and 177XbaIR with purified plasmid pACYC177 as a template, and PCR amplification using Promega GoTaq^®^ long PCR Mastermix according to the manufacturer’s instructions. The PCR product results in amplification of the plasmid sequence without the ampicillin resistance cassette gene of the original plasmid, and which has been replaced with an X*ba*I site. Restriction digestion of the resulting PCR product with X*ba*I and re-ligation with T4 DNA Ligase (Promega) was followed by transformation of 100 µl of CaCl2 competent *E. coli* DH10B cells with 5 μl of the ligation products according to standard protocols. After a centrifugation step, pellets were resuspended in 200 µl LB and plated onto agar plates containing 50 µg/ml neomycin. After overnight incubation at 37 °C, successful transformants were identified by colony PCR using the Promega GoTaq DNA Polymerase (M300) Protocol, including the 5× Green GoTaq^®^ Reaction Buffer 1 (7,5 mM MgCl_2_) and primers 177seqF/R, resulting in the modified vector, pACYC177XbaI. Plasmids for complementation of the Δ*dacB* or Δ*ampD* deletion mutations were constructed by cloning PCR products encoding the intact *dacB* or *ampD* genes derived from template DNA of the ECC (WT) source population IMT 49658-1 using the Promega GoTaq^®^ long PCR Mastermix according to the manufacturer’s instructions and the primer combinations For/Rev_dacB_comp_XbaI and ampDIMT1_XbaI_F/R, respectively. Subsequently, 1 µg DNA of both the plasmid and the insert (*dacB/ampD* PCR product) was digested with *Xba*I, and ligated with plasmid pACYC177XbaI, also digested with X*ba*I. The correct insertion in pACYC177XbaI and the integrity of the *dacB* and *ampD* genes were confirmed by Sanger sequencing (LGC Genomics GmbH) of PCR products. Transformation of pACYC177XbaI_*dacB* or pACYC177XbaI_*ampD* clones into *E. coli* DH10B and the corresponding *dacB* or *ampD*_ev_mutants were confirmed by colony PCR using primers 177seqF/R. The complemented Δ*dacB* or Δ*ampD* deletion mutant strains and *dacB* or *ampD*_ev_mutants were subsequently used for agar-disk diffusion assays as described above to evaluate the heteroresistant phenotype in the presence of CAZ.

### Stability analysis of antibiotic resistance

Stability analysis of antibiotic resistance was performed using ECC *dacB*_ev_mutant (37) and ECC *ampD*_ev_mutant (39). Three single colonies originating from overnight growth on MH-II plates of each strain were incubated separately in 6 mL MH-II liquid broth without antibiotics at 37 °C and 200 rpm. Subcultures were made every 2.5 h (max. 5 generations) with a 1:100 dilution in fresh MH-II liquid broth to ensure that growth did not exceed the late exponential phase. At time points t0, subcultivation 4, 8, 12 and 16, we generated bacterial pellets, which were washed in NaCl and further processed as described in the section ‘agar diffusion tests’ to test the resistance to CAZ.

### DNA/RNA extraction

All DNA isolations were performed using standard protocols. Isolation of genomic DNA from bacterial suspension cultures of potential *dacB* or *ampD*_ev_mutants of the evolution experiments with CAZ plates was performed with the QIAamp DNA Mini Kit (Qiagen) and the isolated DNA used for qPCR to detect GDAs, for PCR product processing for Sanger sequencing of *dacB* or *ampD*, or WGS in selected mutants. The high molecular weight DNA used for qPCR and WGS of all replicates obtained in long-term evolution in 32 µg/ml CAZ to analyse GDA alterations during the evolution experiment in WGS was performed using the Lucigen/Epicentre pure genomic DNA purification Kit (Biozym). RNA extraction was performed for the ECC (WT) source population IMT49658-1 and the *dacB or ampD_ev_mutants* after growth to OD_600_ 0.5 with standard protocols of purification of total RNA from bacteria with the RNeasy Mini Kit from Qiagen. RNA was used for subsequent RT-qPCR gene expression measurements of β-lactamases.

### Whole genome sequencing

Illumina short-read sequencing was performed for all five replicates of long-term evolution in 32 µg/ml CAZ at time points 0, 5, 10, 15 and 20 days post-inoculation, as well as of selected colonies from evolution on 128 µg/ml CAZ plates (see Supplementary Table S2). Isolates were subjected to WGS using the Illumina MiSeq platform (Illumina, Inc., San Diego, USA). From the extracted DNA, 1 ng was used for the library preparation using the Nextera XT DNA Library Preparation Kit (Illumina) according to the manufacturer’s recommendations. The 2×300-bp paired-end sequencing in 40-fold multiplexes was performed on the Illumina MiSeq platform with the MiSeq® Reagent Kit v3 (600 cycle; Illumina Inc., San Diego, CA, USA). The 300-bp paired-end reads were trimmed with Trim Galore v0.6.7 (https://www.bioinformatics.babraham.ac.uk/projects/trim_galore/) and quality-checked with FASTQCv0.11.9 (https://www.bioinformatics.babraham.ac.uk/projects/fastqc/). Unicycler v0.4.9^77^ was used for de novo assembly at default settings, and the resulting contigs were annotated with Prokka v1.14.5^78^. Long-read sequencing was performed for ECC *ampD*_ev_mutant IMT 49658-1 (39) and long-term evolution isolates in 32 µg/ml CAZ ECC IMT 49658-1 (7) at time 0, 5, 10, 15, and 20 days (d), and ECC IMT 49658-1 (13) at times 5, 10, 15, 20 d, ECC IMT 49658-1 (8) at times 0, 5, 10, and 20 d, and ECC IMT 49658-1 (9) at times 5 and 15 d. The isolates were subjected to long-read sequencing using Oxford Nanopore MinION (Oxford, UK). One-dimensional (1D) MinION libraries were prepared using the Rapid Sequencing Kit (SQK-RBK004; Nanopore Technologies, Oxford, UK) and loaded onto an R9.4.1 flow cell according to the manufacturer’s instructions. Sequencing data was then collected for a total of 12 h. 400 ng of extracted DNA was used as starting material for sequencing by MinION technologies. Reads for all Nanopore data sets were basecalled and demultiplexed with Guppy basecaller v6.5.7 (Oxford Nanopore Technologies, Oxford, UK) into quality-tagged sequence reads (4000 reads per fastq-file). In addition, Porechop v0.2.4 (https://github.com/rrwick/Porechop and Filtlong v0.2.1 (https://github.com/rrwick/Filtlong) were used for adapter trimming, elimination of reads below 1000 bp, or sequences of quality below QV 7.5. Finally, a length quality check of the raw reads was performed with LongQC-1.2.0c^79^ (https://github.com/yfukasawa/LongQC). Closed genomes were generated with Flye 2.9^80^ assembler using Oxford Nanopore long-reads and polished with NextPolish v1.4.1^81^ using Illumina short-reads.

### SNP-analysis

Sequences of all replicates of the long-term evolution in 32 µg/ml CAZ and the population-rescue/hindering of SNPs via.GDA experiment, ECC *dacB*_ev_mutant (37), ECC *ampD*_ev_mutant (39) and IMT 49658-1 (48), (51), (59), (141), (151), (153), (154), (155), (168), (175) from evolution on 128 µg/ml CAZ plates were analysed for SNPs using Geneious 11.1.5. The Illumina MiSeq short reads of the indicated isolates were mapped separately to the non-evolved reference genome, IMT 49658-1, which has been previously sequenced (Kupke et al.)^31^ using the Geneious algorithm and the ‘mapping to reference’ option in Geneious. We subsequently used the ‘Find Variations/SNPs’ feature from the ‘Annotate & predict’ menu to identify SNPs. We specified that SNPs with a minimum variant frequency of 0.25 were recognized, i.e. a SNP must occur in at least 25% of all available mapped illumina raw data. In addition, SNPs with a variant *p* value < 0.05 were considered to improve the likelihood that a SNP represented a genuine variant and was not a sequencing error. To detect new arising SNPs, a minimum variant frequency of 0.05% was used in selected replicates of the long-term evolution. The SNPs were exported to an Excel file (Microsoft), and all SNPs of the different isolates were differentiated with the VLOOKUP formula in Excel. In this procedure, those SNPs that only occur in isolates with reduced or no GDA identified by qPCR (see method section detection of GDAS with qPCR) were of particular interest, as SNPs that could only be found in these isolates were most likely to contribute to the reduction of amplifications. To filter out results originating from sequencing errors, potential SNPs identified when comparing Illumina raw reads of the non-evolved strain with its own reference genome were excluded from the beginning of the SNP analysis. The final approval of a SNP for a possible role in GDA-reduction was performed by complementation experiments (see Complementation experiments above). For all further SNP analysis for colonies from 128 µg/ml CAZ plates without WGS data, we first performed a detection of GDAs with qPCR and then examined all colonies with reduced GDAs. Here, we performed PCR with the Promega GoTaq DNA Polymerase (M300) Protocol, including the 5x Green GoTaq^®^ Reaction Buffer 1 (7,5 mM MgCl_2_) and using the primers dacB_For_IMT1 and dacB2.2_Rev_IMT1 for the *dacB* gene, and primers Neu_ampD_IMT1_For and Neu2_ampD_IMT1_Rev for *ampD*. After purification of the PCR products of *dacB* and *ampD* reactions with the QIAquick PCR purification Kit (Qiagen), the PCR products were prepared for Sanger sequencing at LGC Genomics GmbH according to the manufacturer´s instructions. Briefly, 4 µl of 5pMol primers were added to 10 µl of DNA (>40 ng/µl) per sample and subsequently sequenced at LGC Genomics GmbH. The primers For_dacB_comp_XBAI, dacB2.2_Rev_IMT1, dacB2_For_IMT1 and dacB3_For_IMT1 were used to cover the *dacB* PCR product and Neu_ampD_IMT1_For and Neu2_ampD_IMT1_Rev for the *ampD* PCR product. The sequences obtained were then mapped to the reference sequences of *dacB* and *ampD* from ECC (WT) IMT 49658-1 using the Geneious algorithm and the ‘mapping to reference’ option in Geneious to detect potential SNPs after evolution on 128 µg/ml CAZ plates. Primer sequences are listed in Supplementary Table S1. Finally, complementation was performed in selected mutants as described above.

### Detection of gene duplication-amplifications (GDAs) with qPCR

GDA copy number was determined by qPCR as previously described^31^ for all replicates in long-term evolution with 32 µg/ml CAZ and from selected colonies from evolution on 128 µg/ml CAZ plates (see Supplementary Tables S3.1–S3.4) using a PCR cycler StepOnePlus^TM^ (Thermo Fisher Scientific) and Power SYBR Green Master Mix (Thermo Fisher Scientific). Genomic DNA (gDNA) was extracted as described above. Subsequently, 15 µl Mastermix and 5 µl (6,7 ng) gDNA were used in a final volume of 20 µl per well in 96-well plates (Eppendorf twin.tec® PCR Plate 96, unskirted, white). qPCR was performed in triplicate per primer pair. The Ct values of *bla*_DHA-1_, (*pspA* in the *bla*_DHA-1_ deletion mutant), represented the amplified region relative to the housekeeping gene tryptophan synthetase β-chain (*trpB*) used as a control sequence remote from the GDA. The resulting data were calculated using the 2^-ΔΔCt^ method^82^. Briefly, normalisation to the *trpB* gene was performed to obtain ΔCt values and then the relative fold-change values were calculated. To do this, the GDA copy number of the ΔCt values of the evolved replicates ‘sample’ was subtracted from the susceptible replicates ‘control’ to obtain ΔΔCt, which was used in the 2^-ΔΔCt^ formula. Finally, a graphical representation with median and IQR per analysed gene was created in GraphPad Prism.

### Gene expression analysis with RT-qPCR

RT-qPCR was performed similar as previously described^31^. ECC_*dacB*_ev mutant (37) and ECC *ampD*_ev_mutant (39) have been used for genes *AmpR*/*bla*_DHA-1_, *bla*_DHA-1_, *AmpR/ bla*_ACT-16_, *bla*_ACT-16,_ *bla*_TEM_ and *bla*_CTX_. The same PCR cycler, Mastermix and 96-well plates were used as in the ‘Method’ section ‘Detection of GDAs with qPCR’. First, the standard protocols of the RNeasy Mini Kit (Qiagen) were applied for RNA extraction as described in the ‘Method’ section ‘DNA/RNA extraction’, including an on-column DNA digestion step with the RNAse-Free DNase Set from Qiagen. RNA concentration and purity were determined using a NanoDrop 1000 Spectrophotometer (Thermo Fisher Scientific) (A260/A280; ≥1.9, A260/A230 ≥ 1.9). Subsequently, a control-qPCR was performed for the RNA purity with 25 ng/well of DNase-digested RNA. In case of DNA contamination (Ct earlier than 35), we repeated DNA-digestion with RQ1 RNase-free DNase (Promega) according to the manufacturer´s instructions. Finally, 840 ng non-contaminated RNA was used to generate complementary DNA (cDNA) according to the manufacturer’s instructions with RevertAID and Riboblock (reverse transcriptase and RNase inhibitor, Thermo Fisher Scientific). Then, qPCR was carried out with 42 ng/well cDNA and technical triplicates per tested gene, in 96-well plates. For gene expression analysis we used the 2^-ΔΔCt^-method as described in the method-section ‘Detection of GDAs with qPCR’.

### Minimum inhibitory concentration (MIC) assays

MIC assays were performed for the evolved ECC *dacB*- and *ampD* mutants, originating from the single-step evolution experiment on plates containing 128 µg/ml ceftazidime (ECC_*dacB*_ev_mutant (37) & ECC_*ampD*_ev_mutant (39)) in MH II broth with ceftazidime (CAZ) as the antibiotic of interest. Briefly, a 12-step twofold serial dilution series of the antibiotic, ranging from 1.95 to 4000 µg/ml, was prepared in untreated flat-bottom polystyrene 96-well plates (Costar/Corning 3370; Corning Incorporated, 2 Alfred Road, Kennebunk, ME 04043, USA) and an inoculum of ~10^5^ CFU/ml was then added to triplicate wells for each CAZ dilution. Immediately after inoculation of all wells (*t* = 0), the optical density for each well of the plates was determined using a BioTek Agilent *Synergy HTX* Multimode *Plate Reader (BioTek)* at 600 nm (OD600) and 460 nm, followed by incubation of the plates at 37 °C for 20 h. At *t* = 20 h, the optical densities were again determined. The MIC was defined as the lowest antibiotic concentration resulting in an OD600 value within the background range (OD600 < 0.1) after 20 h of incubation. For each assay and strain, the controls included wells without antibiotics, the ECC (WT) parent population IMT 49658-1, and growth media alone (typically OD = 0.08–0.09). MIC experiments were performed in three independent experimental repeats, with three technical replicates included per experiment. Individual replicate values are provided in Supplementary Table S10.

### Mathematical modelling of population dynamics

The model used in this study is based on previous models of GDA and population dynamics^25,39,41,83^. The model is set up to simulate the experiments performed in Fig. 1B, i.e. the population grows to a finite population size until the growth-limiting resource is depleted. In the model, subpopulations carrying different numbers of GDAs are constantly generated and lost^39,40^. Additionally, the abundance of GDAs in the population changes due to competition. Each GDA increases the capability to degrade CAZ, thereby changing the MIC of the subpopulation, and is associated with a fitness cost in terms of growth rate. Evolved resistant mutants (*dacB* or *ampD*) with experimentally determined fitness costs (Fig. 1D, E) and MIC values (Supplementary Table S10) emerge in the model at a rate set by the mutation rate observed in previous studies for *Enterobacter* spp^52^. Resistant mutants (*dacB* or *ampD*) emerge at the same rate in all cells, independent of the number of GDAs that are present in these cells. In the *dacB* and *ampD* background, we assume that GDAs do not provide a benefit but maintain their fitness cost^39^. The model was parameterized based on literature values and experimental data from this study (Fig. 1A, D, E) and then evaluated based on the model’s ability to reproduce the experimental observations in Fig. 1A (Supplementary Table S12, and Supplementary Fig. S11).

The model assumes a constant, identical recombination rate (*k*_*rec*_) per GDA copy *n*, resulting in the recombination probability *p*_*rec*_:

1 $${p}_{{rec}}=\frac{{k}_{{rec}}\left(n-1\right)}{1+{k}_{{rec}}\left(n-1\right)}$$

Here, the model was extended to account for different antibiotic concentrations, competition for a common resource, and cyclic dilution into fresh medium. In the model, fitness as function of antibiotic and number of GDAs is described by a dose-response curve such that fitness *w* as function of CAZ concentration *c* is given by:

2 $$w\left(c\right)=min +\frac{\left(1-min \right)}{1+{\left(c/I{C}_{50}\right)}^{p}}$$

where *min* is the lower asymptote of the curve, *p* is the Hill coefficient related to the steepness of the curve and *IC*_*50*_ is the concentration of CAZ that reduces fitness by 50%. Here, *min* is set to a negative value, which allows calculation of the MIC as the concentration at which fitness *w(c)* = 0. Negative fitness values are set to 0. In the model, the benefit *b* provided by carrying *n* GDA copies is incorporated by multiplying the copy number by the benefit per copy number, with the exponent *a* governing whether the benefit of additional GDA copies scales linearly (*a* = 1; as in the main text), sub-linearly with diminishing returns (*a* < 1), or accelerates (*a* > 1), giving the *IC*_*50*_ as:

3 $${IC}50\left(n\right)=b{n}^{a}$$

A fitness burden *d* per copy number *n* incurred by carrying GDAs is calculated as (1-*d*)^*n-1*^ and multiplied by the fitness calculated by the dose-response curve in Eq. (2). When no CAZ is present, GDAs only incur costs, yet GDAs are still generated via duplication and recombination events. If mutations in *dacB* or *ampD* occur, which are independent of the presence or number of GDAs, the benefit of the GDAs is lost, and only costs are incurred, selecting against mutants that carry GDAs. The IC_50_ is then set to a value based on the experimental measure of the MIC of the mutant, and the fitness of the mutant without GDAs is set to the experimentally determined values (Fig. 1).

Because experimental parameters are important in evolution and selection experiments, the model is implemented to mimic growth in a defined volume of growth medium, from which nutrients are consumed by the cells, followed by dilution into fresh medium. Genotypes with a cell number of less than one after dilution are set to zero upon dilution.

The model is initialized to start from an individual cell, which forms a colony and initially carries one copy of the fragment amplified in ECC. During the growth of the colony, the population is seeded with cells carrying different numbers of GDAs through duplication and recombination. This in silico colony is then used to inoculate a defined volume of growth medium with or without CAZ. Our data suggest that *dacB*- and *ampD* mutations are frequent enough to not be diluted out under our experimental conditions.

Model parameters were determined experimentally, taken from literature or, if not available, estimated by calibrating the model on the GDA-fold-change per CAZ from Fig. 1A (Supplementary Fig. S11, and Supplementary Table S12).

Key assumptions of the model:

- The cost per GDA is constant and independent of the copy number; total fitness cost is multiplicative^25,39,41^.
- GDAs lose their benefit in *dacB/ampD* background^39^.
- Mutations in *dacB* and *ampD* occur independently of presence or number of GDAs.

### Experimental testing of GDA mediated population rescue and delay of SNP-mutant fixation

We performed the experiment with the ECC (WT) parent population and the GDA-deficient *bla*_DHA-1_ knockout mutant, ECC Δ*catA2*Δ *bla*_DHA-1_, based on the model results to GDA-driven population rescue and hindering of SNPs. After overnight growth on MH II plates, individual colonies of the respective strains were incubated in 6 ml MH II broth and grown at 37 °C and 200 rpm until the exponential phase (OD_600_ 0.5). The bacterial suspensions were then inoculated into 6 ml MH II broth with 32 µg/ml (two-fold the breakpoint concentration for CAZ and *Enterobacterales*^46^) CAZ with concentrations of 10^1^, 10^3^, 10^5^, 8 × 10^6^ and 5 × 10^7^ CFU/ml. Each dilution/initial inoculum was incubated in triplicate, except for 13 tubes for the 10^5^ CFU/ml starting inoculum. After 24 h at 37 °C and 200 rpm aeration, the cultures were visually examined for growth, and the results were noted. Subsequently, pellets were prepared for the ECC (WT) parental population and the ECC Δ*catA2*Δ *bla*_DHA-1_ from three grown replicates per strain, and the subsequent DNA was extracted using the QIAamp DNA Mini Kit (Qiagen) (see method section ‘DNA/RNA extraction’). To detect amplification of the gene duplication, qPCR was performed for all replicates (see section ‘Detection of gene duplication and amplification (GDAs) by qPCR’) and SNP analysis was performed after Sanger sequencing on the *dacB* and *ampD* gene (see section ‘SNP analysis’).

### Fluctuation assays

To determine mutation rates, fluctuation assays were performed with the ECC (WT) parental population and the GDA-deficient *bla*_DHA-1_ knockout mutant, ECC Δ*catA2*Δ *bla*_DHA-1_. After a single colony of each strain was grown at 37 °C and 200 rpm to log-phase, a volume of 20 ml Mueller Hinton (MH II) broth with a bacterial concentration of 10^4^ cells/ml was prepared. Untreated flat-bottom polystyrene 96-well plates (Costar/Corning 3370; Corning Incorporated, 2 Alfred Road, Kennebunk, ME 04043, USA) were used to produce 48 replicate cultures from both strains, each containing 200 µl with a starting inoculum of 2 × 10^3^ cells. In addition, three replicates of MH II were produced as negative controls and six replicates for the estimation of population size per strain. After overnight incubation at 37 °C, replicates were plated onto MH II agar plates containing nalidixic acid (64 µg/ml) for enumeration of resistant mutants and onto MH II plates without antibiotics for population size estimation. Colony count was performed after overnight incubation at 37 °C, and the results were calculated using the Maximum likelihood method on the website https://shinyflan.its.manchester.ac.uk/ based on the R-package flan (Supplementary Table S13).

### Statistics

Statistical analysis was performed using GraphPad Prism. A value *p* < 0.05 was considered statistically significant. Significance of the appearance times from ECC (WT) parental population, its heteroresistant phenotype and evolved *dacB*- and *ampD* mutants in ScanLag was tested with Mann–Whitney-U Test (Fig. 2D, and Supplementary Fig. S7.1) and data presented with median and interquartile range (IQR). Significance of Colony pixel-sizes of the ECC (WT) parental population, its heteroresistant phenotype, the evolved *dacB*- and *ampD* mutants, and their respective knockout strains in ScanLag at t24 hours were calculated with the Kruskal–Wallis test (Fig. 2E, Supplementary Fig. S7.1 inset, and Supplementary Fig. S7.2). In addition, we performed a one-sample t-test against zero for the selection coefficients and against one for the competition indices of the competition assays to analyse significant differences in fitness between strains (Fig. 1D, E, and Supplementary Tables S8.1 and S8.2). Data was presented with mean and standard deviation (SD).

## Supplementary information

Supplementary information

## Data Availability

The sequencing data of the *Enterobacter cloacae* complex (ECC), *Enterobacter hormaechei*, IMT 49658, used for this study, can be found in the NCBI Sequence Read Archive (NCBI bioproject number: PRJNA1020684, biosample number: SAMN37527804, accession numbers CP135270-CP135274). Raw data for the generation of the main figures or any additional information required to reanalyze the data reported in this paper is available from the corresponding authors upon request.

## Code availability

The Python code of the deterministic model generated during the current study is available at https://github.com/nnordhol/GDA_dynamics_model/.
