# Supplementary material for: Population size modulates the transition from gene amplification-mediated heteroresistance to stable resistance
Source: NPJ Antimicrob Resist. 2026 Oct 9;4:65. doi: 10.1038/s44259-026-00280-x (PMC13652803; doi:10.1038/s44259-026-00280-x)
Supplement: Supplementary file 1 [file 44259_2026_280_MOESM1_ESM.pdf]

Supplementary materials for

Population size modulates the transition from gene amplification-mediated heteroresistance to stable resistance

J. Kupke, Y. Fang, F. Ghazisaeedi, D. Hanke, F. Schreiber, K. Tedin, N. Nordholt* & M. Fulde*

*Corresponding authors: marcus.fulde@tiho-hannover.de (M.F.) ; niclas.nordholt@bam.de (N.N.)

Content of this supplementary file 1

- Table S1. Primers used for this study
- Table S2. Bacterial strains used for this study
- Table S3.1 Lag times and reduced gene duplication-amplification (GDA) copy numbers in evolved *dacB*- and *ampD_ev*_mutants evolved on 128 µg/ml ceftazidime (CAZ) plates
- Table S3.2. Lag times and GDAs of ECC GDA containing resistant subpopulations (CFUs) from 128 CAZ plates in ScanLag
- Table S3.3. Reduced gene duplication-amplification (GDA) copy numbers in evolved *dacB* mutants evolved on 128 µg/ml ceftazidime (CAZ) plates.
- Table S3.4. GDAs of ECC GDA containing resistant subpopulations (CFUs) from 128 CAZ plates.
- Table S4. Overview of mutations in *dacB*_ev_mutants from evolution in 32 µg/ml CAZ liquid broth and on 128 µg/ml CAZ plates
- Table S5. Overview of mutations in *ampD*_ev_mutants from evolution on 128 µg/ml CAZ plates.
- Table S6. Relative Growthrates of ECC *dacB*- and *ampD*_ev_mutants and the corresponding knock-out strains
- Table S7. Lag times of the *dacB*- and *ampD*_ev_mutants compared to the *Enterobacter cloacae* complex IMT 49658 (ECC WT).
- Table S8.1 competitive indexes (CI) of competition assays for different strain combinations.
- Table S8.2. Selection coefficients (*s*) of competition assays for different strain combinations.

- Table S9. lag times of the *dacB- and ampD* knockout-strains compared to the *Enterobacter cloacae* complex IMT 49658 (ECC WT).

- Table S10. Minimum inhibitory concentration (MIC) assays comparing the ECC (WT) parental population with the *dacB*- and *ampD* mutants.

- Table S11.1. Experimental results on long-term evolution in 32 µg/ml CAZ with the ECC (WT) parental population and its GDA-deficient counterpart.

- Table S11.2. Experimental results for gene-duplication amplification (GDA)-mediated population rescue and hindering of SNP-mutants.

- Table S12: Model parameters for the deterministic modelling of evolutionary dynamics in *Enterobacter cloacae* complex (ECC) IMT 49658.

-Table S13: Comparison of mutation rates in the ECC (WT) parental population and the GDA deficient counterpart

- Figure S1. Schematic overview of the experimental design for the evolution under A) high ceftazidime (CAZ) pressure on agar plates and B) under long-term CAZ pressure in liquid broth.

- Figure S2. Fold change β-lactamases gene expression in *dacB- and ampD_ev_*mutants.

- Figure S3. IMT 49658-1 (ECC) Δ*catA2*Δ*dacB* and IMT 49658-1 (ECC) Δ*catA2*Δ*ampD* and their corresponding complemented strains in agar-disk diffusion.

- Figure S4. Stability of resistance in evolved ECC *dacB*- and *ampD_ev_*mutants.

- Figure S5. IMT 49658-1 (ECC) *dacB_ev_mutants* and their corresponding complemented strains in agar-disk diffusion.

-Figure S6. IMT 49658-1 (ECC) *ampD_ev_mutants* and their corresponding complemented strains in agar-disk diffusion.

- Figure S7.1. Lag times and colony size distribution at t 24 hours of ECC *dacB*- and *ampD*_ev_mutants in ScanLag without CAZ.

- Figure S7.2. Distribution of colony sizes of *dacB*- and *ampD* knockout strains and ECC (WT) parental population at time point t 24 in ScanLag.

- Figure S8. Loss of heterogeneity in the IMT 49658-1 (ECC) *ampD*_ev_mutant.

- Figure S9. Comparative growth of the ECC (WT) parental population and the ECC *dacB*_ev_mutant in different ceftazidime (CAZ) concentrations.

-Figure S10. Growth inhibition as a function of antibiotic concentration (MIC Assay) of the ECC (WT) parental population and the ECC *dacB* – and *ampD*_ev_mutant.

- Figure S11. Model calibration and analysis.

-Figure S12. Influence of benefit function form and parameter values of cost and benefit on model predictions.

-Figure S13. Influence of parameter values of duplication rate and recombination rate on model predictions.

-Figure S14. Exploring the influence of different fitness cost functions on model predictions and the model’s ability to reproduce the average GDA copy number under different CAZ concentrations.

Table S1. Primers used for this study

Name of primers	Detected gene	Sequence 5´- 3´	Used for the method
blaDHA-1 forward *	*bla*$_{DHA-1}$	GTTCAGCCGTATAACCGTGCTTC	qPCR (gDNA,
blaDHA-2 reverse *		TCACAATCGCCACCTGTTTTTCC	cDNA); PCR
transreg_AmpR_forward *	*ampR/bla*$_{DHA-1}$	CACACAGCCGAGTTGTTCTTCC	qPCR (cDNA)
transreg_AmpR_reverse *		CAGACGTTATCTCCCCCTTAACCC	
bla_ACT-16_forward *	*bla*$_{ACTH-16}$	AGCCCAGACCCTGATACATTGAC	qPCR (cDNA)
bla_ACT_16_reverse *		CAACGTGCAGGATATGGCGAAC	
Regulator AmpR forward *	*ampR/bla*$_{ACTH-16}$	TGACCCATTCAGCCATTAGCCAG	qPCR (cDNA)
Regulator AmpR reverse *		AAAGAATCATTCAGCACCGGCAG	
blaTEM forward *	*bla*$_{TEM-1}$	CTGCAACTTTATCCGCCTCCATCC	qPCR (cDNA)
blaTEM reverse *		ATACCAAACGACGAGCGTGACACC	
Bla_CTX_M-3 forward *	*bla*$_{CTX-M-3}$	TACCGAGCCGACGTTAAACACC	qPCR (cDNA);
Bla_CTX_M-3 reverse *		TGTCGCCCAATGCTTTACCCAG	PCR
For_psp_qPCR *	*pspA*	TCATCGGTGAACCCAGGAAACAACG	qPCR (gDNA)
Rev_psp_qPCR *		CGTTACGCGAACGTCAAAGCGATAT	
trpA (EC) primer forward *	*tryptophan*	CTACGACAACGCATTTGCACAAC	qPCR (gDNA,
trpA (EC) primer reverse *	*synthetase β-chain*	TCGATAATCTTCAGCGACTGCTCC	cDNA)
177XbaIF		GACTAGCTTCTAGAGCATTGGTAACTGTCAGACCAAG TTTACTC	Introduction of XBAI into
177XbaIR		ATCGGCATTCTAGAGTGCCACCTGACGTCTAAGAAAC C	pACYC177
177seqF		CTTGAAGACGAAAGGGCCTC	
177seqR		AAGGATCTTCACCTAGATCC	
For_dacB_comp_XbaI	*dacB*	CATGCATGTCTAGAGCTAACCCTTCCTGCAATAACA	*dacB* ligation into
Rev_dacB1_comp_XbaI		AGCTAGCTTCTAGACAGGTCAGCAGCAATTTTTCAAT	pACYC177_XbaI
ampDIMT1_XBAI_F	*ampD*	GACTAGCTTCTAGAGGCATGATAAAACTCCAAATTGG T	*ampD* ligation into pACYC177_XbaI
ampDIMT1_XBAI_R		ATCGGCATTCTAGACCAGCAGCATGGTAAACAACG	
dacB_For_IMT1	*dacB*	GTATTCACCGCTCTGGATCA	SNP Analysis
dacB2.2Rev_IMT1		ATTTGATACGCATTTTACCGCG	(*dacB*)
dacB2_For_IMT1		CATCGTGACCGGGTAGTAA	
dacB3_For_IMT1		CAAGCGGCAGCATCGTAAT	
Neu_ampD_IMT1_For	*ampD*	GGCGGCATGATAAAACTCCA	SNP Analysis
Neu2_ampD_IMT1_Rev		ATGATCCAGATGCCAGTGC	(*ampD*)
For_konstit_mScarlet_NheI *	mScarlet	GACTAGCTGCTAGCCCATAAACTGCCAGGAATTGGG GATC	constitution of pProbe´-gfp
Rev_konstit_mScarlet_EcoRV *		ATCGGCATGATATCGGATTCTCACCAATAAAAAACGC CCG	(LAA)+mScarlet
p49658catA2H1P1 *	*catA2 pIncHI2/2A*	GCCTTCTGATTATTAATATTTTTCACTATTAATCAGAA GGAATAACCATGTGTAGGCTGGAGCTGCTTCGA	Wanner mutagenesis
p49658catA2H2P2 *		ATCCTGCCGGATACGGTGGCTTAAATACAGAATTAAT TAATTTATTTCAGCATATGAATATCCTCCTTAG	
49658catF3 *		GCGCCTGGTAAGCAGAGTT	
49658catR2 *		CCAGACAGCTTACGGAGGAC	
49658_DACBH1P1	*dacB*	CATGCACCGTAGTATGACGGTTTGTTTTCGGGTTGTT AGCGCGAGATTATGTGTAGGCTGGAGCTGCTTCGA	Wanner mutagenesis
49658_DACBH2P2		TTCAACTATCAGTAGTTTCATCGCTAGTTATTCTGGTA GATGTCCTTATAGCATATGAATATCCTCCTTAG	
49658_dacB_F		CCAGAAGCAAAAAGAAAACCGAC	
49658_dacB_R		TATTCACCGCTCTGGATCAGG	

49658_AMPDH1P1	*ampD*	CATGCTACTCTGAACCGAGTAACAGCACCATAGATAA GGAGTTCCAGCATGTGTAGGCTGGAGCTGCTTCGA	Wanner mutagenesis
ampDreserve (2.H2P2 primer)		ATAGCGCCCGATTAACGTTTGCGTTACAGCAACCAGT TTCTCATATTGCGCCATATGAATATCCTCCTTAG	
49658_AMPDF		GGCATGATAAAACTCCAAATTGGT	
49658_AMPDR		AACGTAATGGCCATCACGCC	

*also used in previous study (Kupke *et al.*) [1]

Table S2. Bacterial strain used for this study: *Enterobacter cloacae* complex IMT 49658

Strain	General explanations	characteristics to ceftazidime (CAZ)	WGS	Explanations to genotype	Reference
**IMT 49658-1 (ECC(WT) parental population)**	Source/parental population.	Heteroresistant	Oxford Nanopore MinION & llumina MiSeq (sequenced in previous study,Kupke *et al.*[1]	Under ceftazidime (CAZ) pressure gene duplication-amplification (GDA) create resistant subpopulations (Heteroresistance)	Our laboratory (NCBI biosample number: SAMN37527804, accession numbers CP135270-CP135274) previous study, Kupke *et al.*[1]
**IMT 49658-1 (7) ev0**	CFU of IMT 49658-1 originating from 32 µg/ml CAZ plate in ScanLag	Resistant mutant	Oxford Nanopore MinION & Illumina MiSeq	*dacB* mutation	This study
**IMT 49658-1 (7) ev5, ev10, ev15, ev20**	IMT 49658-1 (7) evolved in 32 µg/ml CAZ liquid broth - day 5, 10, 15 and 20	Resistant mutant	Oxford Nanopore MinION & Illumina MiSeq	*dacB* mutation	This study
**IMT 49658-1 (8) ev0**	CFU of IMT 49658-1 originating from 32 µg/ml CAZ plate in ScanLag	Resistant mutant	Oxford Nanopore MinION & Illumina MiSeq	GDAs	This study
**IMT 49658-1 (8) ev5, ev10, ev15, ev20**	IMT 49658-1 (8) evolved in 32 µg/ml CAZ liquid broth - day 5, 10, 15 and 20	Resistant mutant	Oxford Nanopore MinION (except ev15) & Illumina MiSeq	GDAs	This study
**IMT 49658-1 (9) ev0**	CFU of IMT 49658-1 originating from 32 µg/ml CAZ plate in ScanLag	Heteroresistant subpopulation	Illumina MiSeq	GDAs	This study
**IMT 49658-1 (9) ev5, ev10, ev15, ev20**	IMT 49658-1 (9) evolved in 32 µg/ml CAZ liquid broth - day 5, 10, 15 and 20	Heteroresistant subpopulation	Oxford Nanopore MinION (for ev5 & ev15) & Illumina MiSeq	GDAs	This study
**IMT 49658-1 (10) ev0**	CFU of IMT 49658-1 originating from 32 µg/ml CAZ plate in ScanLag	Heteroresistant subpopulation	Illumina MiSeq	GDAs	This study
**IMT 49658-1 (10) ev5, ev10, ev15**	IMT 49658-1 (10) evolved in 32 µg/ml CAZ liquid broth - day 5, 10, 15	Heteroresistant subpopulation	Illumina MiSeq	GDAs	This study

**IMT 49658-1 (10) ev20**	IMT 49658-1 (10) evolved in 32 µg/ml CAZ liquid broth - day 20	Resistant mutant	Illumina MiSeq	*dacB* mutation	This study
**IMT 49658-1 (13) ev0**	CFU of IMT 49658-1 originating from 32 µg/ml CAZ plate in ScanLag	Heteroresistant subpopulation	Illumina MiSeq	GDAs	This study
**IMT 49658-1 (13) ev5, ev10, ev15, ev20**	IMT 49658-1 (13) evolved in 32 µg/ml CAZ liquid broth - day 5, 10, 15 and 20	Heteroresistant subpopulation	Oxford Nanopore MinION & Illumina MiSeq	GDAs	This study
**IMT 49658-1 (48)**	CFU of IMT 49658-1 originating from 4 µg/ml CAZ plate in ScanLag (Fig.1A)	Heteroresistant subpopulation	Illumina MiSeq	GDAs	This study
**IMT 49658-(51)**	CFU of IMT 49658-1 originating from 16 µg/ml CAZ plate in ScanLag (Fig. 1A)	Heteroresistant subpopulation	Illumina MiSeq	GDAs	This study
**IMT 49658-(59)**	CFU of IMT 49658-1 originating from 16 µg/ml CAZ plate in ScanLag (Fig. 1A)	Heteroresistant subpopulation	Illumina MiSeq	GDAs	This study
**ECC *dacB*_ev_mutant (37)** (IMT 49658-1 (37))	CFU of IMT 49658-1 originating from 128 µg/ml CAZ plate in ScanLag (Fig. 1A)	Resistant mutant	Illumina MiSeq	*dacB* mutation	This study
**ECC *ampD*_ev_mutant (39)** (IMT 49658-1 (39))	CFU of IMT 49658-1 originating from 128 µg/ml CAZ plate in ScanLag (Fig. 1A)	Resistant mutant	Oxford Nanopore MinION & Illumina MiSeq	*ampD* mutation	This study
**IMT 49658-1 (153), (168)**	CFUs of IMT 49658-1 originating from 128 µg/ml CAZ plate in ScanLag	Resistant mutants	Illumina MiSeq	*ampD* mutation	This study
**IMT 49658-1 (141), (151), (154)-(155), (175)**	CFU of IMT 49658-1 originating from 128 µg/ml CAZ plate in ScanLag	Resistant mutants	Illumina MiSeq	*dacB* mutation	This study
**IMT 49658-1 (40), (52), (53), (142) - (150), (156)-(160), (169)-(174), (176), (177)**	CFUs of IMT 49658-1 originating from 128 µg/ml CAZ plate in ScanLag	Heteroresistant subpopulation	Illumina MiSeq: IMT 49658-1(155)	GDAs	This study
**IMT 49658-1 (78), (80), (83), (88), (90), (91), (93),**	CFUs of IMT 49658-1 originating from	Heteroresistant subpopulations		GDAs	This study

**(94), (97), (100), (103), (104)**	128 µg/ml CAZ plates				
**IMT 49658-1 (79), (81), (82), (84)-(87), (89), (92), (95), (96), (98), (99), (101), (102), (105)-(107)**	CFUs of IMT 49658-1 originating from 128 µg/ml CAZ plates	Resistant mutants	sequencing at LGC Genomics GmbH for *dacB*/*ampD*	*dacB* mutations	This study
**IMT 49658-1 (pProbe´-gfp (LAA)+mScarlet)**	Used in competition assay	Heteroresistant		No GDAs; Contains plasmid pProbe´-gfp (LAA) for neomycin resistance; with mScarlet red fluorescence protein	Previous study, Kupke *et al.* [1]
**IMT 49658-(52) (pProbe´-gfp (LAA)+mScarlet)**	Used in competition assay. Resistant subpopulation from 128 µg/ml CAZ plate	Resistant subpopulation from		GDAs; Contains plasmid pProbe´-gfp (LAA) for neomycin resistance; with mScarlet red fluorescence protein	This study
**IMT 49658-1 (52)**	Used in competition assay. Resistant subpopulation from 128 µg/ml CAZ plate (Fig.1A)	Resistant subpopulation from		GDAs (~39)	This study
**ECC *dacB*_ev_mutant (37) (pProbe´-gfp (LAA)+mScarlet)**	Used in competition assay	Resistant mutant		*dacB* mutant; Contains plasmid pProbe´-gfp (LAA) for neomycin resistance; with mScarlet red fluorescence protein	This study
**ECC *dacB*_ev_mutant (39) (pProbe´-gfp (LAA)+mScarlet)**	Used in competition assay	Resistant mutant		*ampD* mutant; Contains plasmid pProbe´-gfp (LAA) for neomycin resistance; with mScarlet red fluorescence protein	This study
**IMT49658-1 (26), (27), (41), (42), (54)**	CFU from plate with 0 µg/ml CAZ in ScanLag (Fig. 1A)	susceptible			This study
**IMT 49658-1 (28) - (30) (43), (44), (55),**	CFU from 1 µg/ml CAZ plate in ScanLag (Fig. 1A)	Resistant subpopulations		GDAs	This study
**IMT 49658-1 (31), (32), (45)-(47), (56)**	CFU from 2 µg/ml CAZ plate in ScanLag	Resistant subpopulations		GDAs	This study
**IMT 49658-1 (33), (34), (49), (57), (58)**	CFU from 4 µg/ml CAZ plate in ScanLag (Fig. 1A)	Resistant subpopulations		GDAs	This study
**IMT 49658-1 (35), (36), (50), (60)**	CFU from 16 µg/ml CAZ plate in ScanLag	Resistant subpopulations		GDAs	This study
**IMT 49658-1 (40), (52), (53), (38)**	CFU from 128 µg/ml CAZ plate in ScanLag (Fig. 1A)	Resistant subpopulations		GDAs	This study
**IMT 49658-1 (ECC) Δ*catA2*Δ*dacB***	*Enterobacter cloacae* complex	resistant		Deletion of *catA2, dacB*	This study

	(ECC) knockout strain			
**IMT 49658-1 (ECC) Δ*catA2*Δ*ampD***	*Enterobacter cloacae* complex (ECC) knockout strain	resistant	Deletion of *catA2, ampD*	This study
**IMT 49658-1 (ECC) Δ*catA2*Δ*ampD* (pACYC177_XbaI_*ampD*)**	Complementation of the intact *ampD* in *ampD* knockout strain	heteroresistant	pACYC177_XbaI_*ampD* in *ampD* knockout strain	This study
**IMT 49658-1 (ECC) Δ*catA2*Δ*dacB* (pACYC177_XbaI_*dacB*)**	Complementation of the intact *dacB* in *dacB* knockout strain	heteroresistant	pACYC177_XbaI_*dacB* in *dacB* knockout strain	This study
**ECC *dacB*_ev_mutant (39) (pACYC_XbaI_*dacB*)**	Complementation of the intact *ampD* in *ampD*_ev_mutant from 128 µg/ml CAZ plate	heteroresistant	pACYC177_XbaI_*ampD* in *ampD*_ev_mutant	This study
**ECC *dacB*_ev_mutant (37) (pACYC_XbaI_*dacB*)**	Complementation of the intact *dacB* in *dacB*_ev_mutant from 128 µg/ml CAZ plate	heteroresistant	pACYC177_XbaI_*dacB* in *dacB*_ev_mutant	This study
**IMT 49658-1 (79), (81), (82), (86), (107)**	Complementation of the intact *dacB* in *dacB*_ev_mutants from 128 µg/ml CAZ plate	heteroresistant	pACYC177_XbaI_*dacB* in *dacB*_ev_mutants	This study
**IMT 49658-1 (ECC) Δ*catA2*Δ *bla*$_{DHA-1}$**	*Enterobacter cloacae* complex (ECC) knockout strain	susceptible	Deletion of *catA2, bla*$_{DHA-1}$	Previous study, Kupke et al. [1]

Table S3.1

ECC *dacB*/*ampD*_ev_mutants from 128 µg/ml CAZ plate in ScanLag	CAZ plate [µg/ml]	Gene duplication-amplification (GDA) copy number (qPCR)	Lag time [min.]
ECC *dacB*_ev_mutant (37)	128	2.63	633
ECC *dacB*_ev_mutant IMT49658-1 (141)	128	1.25	591
ECC *dacB*_ev_mutant (151)	128	1.02	576
ECC *dacB*_ev_mutant (154)	128	1.00	607
ECC *dacB*_ev_mutant (155)	128	0.84	578
ECC *dacB*_ev_mutant (175)	128	0.99	637
ECC *dacB*_ev_mutant (7)	32	0.84	498
ECC *ampD*_ev_mutant (39)	128	0.89	513
ECC *ampD*_ev_mutant (153)	128	0.97	637
ECC *ampD*_ev_mutant (168)	128	0.84	591

Table S3.1. Lag times and reduced gene duplication-amplification (GDA) copy numbers in evolved *dacB*- and *ampD*_ev_mutants evolved on 128 µg/ml ceftazidime (CAZ) plates in the ScanLag system. All mutants grew with early lag times.

Table S3.2

ECC GDA containing resistant subpopulations (CFUs) from 128 µg/ml CAZ plates in ScanLag	CAZ plate [µg/ml]	Gene duplication-amplification (GDA) copy number (qPCR)	Lag time [min.]
ECC IMT 49658-1 (142)	128	24.31	667
ECC IMT 49658-1 (52)	128	39.73	665
ECC IMT 49658-1 (53)	128	27.27	1101
ECC IMT 49658-1 (40)	128	21.44	1161
ECC IMT 49658-1 (38)	128	28.46	1205
ECC IMT 49658-1 (143)	128	24.94	758
ECC IMT 49658-1 (144)	128	23.69	728
ECC IMT 49658-1 (145)	128	23.65	728

ECC IMT 49658-1 (146)	128	23.65	788
ECC IMT 49658-1 (147)	128	27.43	879
ECC IMT 49658-1 (148)	32	22.38	1107
ECC IMT 49658-1 (149)	128	12.50	1108
ECC IMT 49658-1 (150)	128	13.20	1199
ECC IMT 49658-1 (152)	128	19.21	667
ECC IMT 49658-1 (156)	128	22.06	1031
ECC IMT 49658-1 (157)	128	14.28	834
ECC IMT 49658-1 (158)	128	11.89	1259
ECC IMT 49658-1 (159)	128	12.13	1199
ECC IMT 49658-1 (160)	128	6.24	1048
ECC IMT 49658-1 (169)	128	22.52	652
ECC IMT 49658-1 (170)	128	20.45	652
ECC IMT 49658-1 (171)	128	17.55	925
ECC IMT 49658-1 (172)	128	17.42	1046
ECC IMT 49658-1 (173)	128	20.46	925
ECC IMT 49658-1 (174)	128	24.70	531
ECC IMT 49658-1 (176)	128	16.45	926
ECC IMT 49658-1 (177)	128	21.14	865

Table S3.2. Lag times and GDAs of ECC GDA containing resistant subpopulations (CFUs) from 128 CAZ plates in ScanLag.

Table S3.3

ECC *dacB*/*ampD*_ev_mutants from 128 µg/ml CAZ	CAZ plate [µg/ml]	Gene duplication-amplification (GDA) copy number (qPCR)
ECC *dacB*_ev_mutant (79)	128	1.99
ECC *dacB*_ev_mutant (81)	128	2.09

ECC *dacB*_ev_mutant (82)	128	1.79
ECC *dacB*_ev_mutant (84)	128	1.22
ECC *dacB*_ev_mutant (85)	128	1.42
ECC *dacB*_ev_mutant (86)	128	1.77
ECC *dacB*_ev_mutant (87)	128	1.83
ECC *dacB*_ev_mutant (89)	128	1.76
ECC *dacB*_ev_mutant (92)	128	2.53
ECC *dacB*_ev_mutant (95)	128	1.07
ECC *dacB*_ev_mutant (96)	128	1.07
ECC *dacB*_ev_mutant (98)	128	1.00
ECC *dacB*_ev_mutant (99)	128	1.80
ECC *dacB*_ev_mutant (101)	128	1.40
ECC *dacB*_ev_mutant (102)	128	1.30
ECC *dacB*_ev_mutant (105)	128	1.26
ECC *dacB*_ev_mutant (106)	128	3.07
ECC *dacB*_ev_mutant (107)	128	1.52

Table S3.3. Reduced gene duplication-amplification (GDA) copy numbers in evolved *dacB* mutants evolved on 128 µg/ml ceftazidime (CAZ) plates.

Table S3.4

ECC GDA containing resistant subpopulations (CFUs) from 128 µg/ml CAZ plates	CAZ plate [µg/ml]	Gene duplication-amplification (GDA) copy number (qPCR)
ECC IMT 49658-1 (78)	128	28.54
ECC IMT 49658-1 (80)	128	22.54
ECC IMT 49658-1 (83)	128	19.91
ECC IMT 49658-1 (88)	128	27.00

ECC IMT 49658-1 (90)	128	14.40
ECC IMT 49658-1 (91)	128	27.67
ECC IMT 49658-1 (93)	128	21.53
ECC IMT 49658-1 (94)	128	22.11
ECC IMT 49658-1 (97)	128	12.86
ECC IMT 49658-1 (100)	128	25.99
ECC IMT 49658-1 (103)	128	23.08
ECC IMT 49658-1 (104)	128	22.02

Table S3.4. GDAs of ECC GDA containing resistant subpopulations (CFUs) from 128 CAZ plates.

Table S4

ECC *dacB*_ev_mutant	CDS position	Polymorphism Type	Amino acid change	Protein Effect
ECC *dacB*_ev_mutant (37) *	1123	SNP (transition) G -> A		Truncation ->protein stop
ECC *dacB*_ev_mutant IMT49658-1 (141)	1210	SNP (transversion) C->A	Gly ->Cys	Substitution
ECC *dacB*_ev_mutant (151)	607	SNP (transition) G->A		Truncation ->protein stop
ECC *dacB*_ev_mutant (154)	607	SNP (transition) G->A		Truncation ->protein stop
ECC *dacB*_ev_mutant (155)	474	Insertion of A		Frame shift -> protein stop
ECC *dacB*_ev_mutant (175)	494	Deletion of GG		Frame shift -> protein stop
ECC *dacB*_ev_mutant(7) *	445	Insertion of G (tandem repeat)		Frame shift ->protein stop
ECC *dacB*_ev_mutant (10) ev20 *	474	Insertion of A		Frameshift ->protein stop
ECC *dacB*_ev_mutant (79) *	98	SNP (transversion) C->A	Gly->Val	Substitution
ECC *dacB*_ev_mutant (81) *	916	SNP (transversion) A->G	Ser->Pro	Substitution
ECC *dacB*_ev_mutant (82) *	1169	SNP (transversion) A->T	Leu->Gln	Substitution
ECC *dacB*_ev_mutant (84)	98	SNP (transversion) C->A	Gly->Val	Substitution
ECC *dacB*_ev_mutant (85)	1169	SNP (transversion) A->T	Leu->Gln	Substitution
ECC *dacB*_ev_mutant (86) *	700	SNP (transversion) C->T	Gly->Asp	Substitution
ECC *dacB*_ev_mutant (87)	1169	SNP (transversion) A->T	Leu->Gln	Substitution
ECC *dacB*_ev_mutant (89)	1169	SNP (transversion) A->T	Leu->Gln	Substitution

ECC *dacB*_ev_mutant (92)	916	SNP (transversion) A->G	Ser->Pro	Substitution
ECC *dacB*_ev_mutant (95)	916	SNP (transversion) A->G	Ser->Pro	Substitution
ECC *dacB*_ev_mutant (96)	1169	SNP (transversion) A->T	Leu->Gln	Substitution
ECC *dacB*_ev_mutant (98)	916	SNP (transversion) A->G	Ser->Pro	Substitution
ECC *dacB*_ev_mutant (99)	98	SNP (transversion) C->A	Gly->Val	Substitution
ECC *dacB*_ev_mutant (101)	1169	SNP (transversion) A->T	Leu->Gln	Substitution
ECC *dacB*_ev_mutant (102)	98	SNP (transversion) C->A	Gly->Val	Substitution
ECC *dacB*_ev_mutant (105)	98	SNP (transversion) C->A	Gly->Val	Substitution
ECC *dacB*_ev_mutant (106)	98	SNP (transversion) C->A	Gly->Val	Substitution
ECC *dacB*_ev_mutant (107) *	1169	SNP (transversion) A->T	Leu->Gln	Substitution

Table S4. Overview of mutations in *dacB*_ev_mutants from evolution in 32 µg/ml CAZ liquid broth and on 128 µg/ml CAZ plates. The table contains information about the SNPs regarding the CDS position, the polymorphism type, if applicable, the amino acid change, and the effects on proteins. Different shades highlight SNPs at the same position.

* = these mutants were complemented with the intact *dacB* gene restoring the heteroresistant phenotype in agar disk diffusion

Table S5.

ECC *ampD*_ev_mutant	CDS position	Polymorphism Type	Amino acid change	Protein Effect
ECC *ampD*_ev_mutant (39) *	499	Deletion of 5 bp (CCGGC)		Frame shift -> protein stop
ECC *ampD*_ev_mutant (153) *	245	SNP (transition) G->A	Gly->Asp	Substitution
ECC *ampD*_ev_mutant (168) *	227	SNP (transition) G->A	Cys->Tyr	Substitution

Table S5. Overview of mutations in *ampD*_ev_mutants from evolution on 128 µg/ml CAZ plates.

* = these mutants were complemented with the intact *ampD* gene restoring the heteroresistant phenotype in agar disk diffusion

Table S6

Strain	Relative growth rate
ECC *dacB*_ev_mutant (37)	0.9605 (+/-0.3)
ECC *ampD*_ev_mutant (39)	0.7019 (+/- 0.14)
ECC Δ*catA2*Δ*dacB*	0.9656 (+/- 0.13)
ECC *ΔcatA2ΔampD*	0,9100 (+/- 0.19)

Table S6. Relative growth rates of ECC *dacB*- and *ampD*_ev_mutants and the corresponding knock-out strains. The *dacB*_ev_mutant and the corresponding knock-out strain have an almost identical growthrate compared to the ECC (WT) parental population. The *ampD*_ev_mutant and the corresponding knock-out strain have higher fitness losses. The relative growth rates were indicated with the median from n= 3 biological replicates with three technical replicates; standard deviations are given in parentheses.

Table S7

	Appearance time/lag time [min] median w/o CAZ	Appearance time/lag time [min] median with CAZ
ECC *ampD_ev_*mutant (39)	530	529
ECC *dacB_ev_* mutant (37)	514	515
ECC (WT) parental population	500	634

Table S7. Lag times of the *dacB*- and *ampD*_ev_mutants compared to the *Enterobacter cloacae* complex IMT 49658 (ECC WT). Without CAZ, a lower loss of fitness is observed for the lag times of the *dacB*_ev_mutants and a higher loss of fitness for the *ampD*_ev_mutant. The lag times for *dacB*/*ampD*_ev_mutants remain similar on plates with CAZ as on plates without CAZ. The lag time median of the ECC WT shifts backwards, as GDA-driven resistant subpopulations appear with very heterogeneous lag times, as previously illustrated [1]. Appearance times were significantly different to each other ($p < 0.0001$, Mann-Whitney-U Test).

Table S8.1

Competition assay	competitive index (CI)
	t 8 [h]
ECC *dacB*_ev_mutant (37) vs. ECC resistant subpopulation with ~ 39 GDAs	1.48 (+/- 0.27); (p=0.0910)
ECC *dacB*_ev_mutant (37) vs. ECC *ampD*_ev_mutant (39)	2.79 (+/- 0.6); (p=0.0357)
ECC *dacB*_ev_mutant (37) vs. ECC *ampD*_ev_mutant (39) in CAZ	1.29 (+/- 0.16); (p=0.0929)
ECC (WT) parental population vs. ECC *dacB*_ev_mutant (37)	1.07 (+/- 0.03); (p=0.0634)
ECC (WT) parental population vs. ECC *ampD*_ev_mutant (39)	3.51 (+/- 0.86); (p=0.0372)

Table S8.1 competitive indexes (CI) of competition assays for different strain combinations. Used strains: ECC resistant subpopulation (=ECC IMT 49658-1 (52)), ECC WT (IMT 49658-1 ECC(WT) parental population), evolved *dacB* mutant (=ECC_*dacB*_ev_mutant (37)) and evolved *ampD* mutant (=ECC_*ampD*_ev_mutant (39)). A competitive index with a value above one shows a better fitness of strain one in competition with strain two. Formula: CI = (strain 1 output/strain 2 output) / (strain 1 input/strain 2 input). The standard deviations of the selection coefficients n= 3 biol. replicates are shown in parentheses. One sample t-tests against one (=no difference in fitness between two strains) show that the observed differences in fitness are significant for the competition of the ECC (WT) vs. evolved *ampD*_ev_mutant and the evolved *dacB* mutant vs. evolved *ampD* mutant, whereas differences in fitness for ECC (WT) vs. evolved *dacB* mutant as well as the evolved *dacB* mutant vs. ECC resistant subpopulation are too minor for a significant differences.

Table S8.2

Competition assay	Selection coefficient *s*
	t 8 [h]
ECC *dacB*_ev_mutant (37) vs. ECC resistant subpopulation with ~ 39 GDAs	0.047 (+/- 0.024); (p=0.0729)
ECC *dacB*_ev_mutant (37) vs. ECC *ampD*_ev_mutant (39)	0.126 (+/- 0.026); (p=0.0135)
ECC *dacB*_ev_mutant (37) vs. ECC *ampD*_ev_mutant (39) in CAZ	0.031 (+/- 0.016); (p=0.08)
ECC (WT) parental population vs. ECC *dacB*_ev_mutant (37)	0.008 (+/- 0.004); (p=0.0628)
ECC (WT) parental population vs. ECC *ampD*_ev_mutant (39)	0.175 (+/- 0.031); (p=0.0105)

Table S8.2. Selection coefficients (*s*) of competition assays for different strain combinations. A value of *s* above zero shows a better fitness of strain one in competition with strain two. Formula: S = (ln(strain 1 output / strain 1 input) – ln(strain 2 output / strain 2 input)) / 8 hours. The standard deviation of n=3 biological replicates is shown in parentheses. One sample t-tests against zero (= no difference in fitness between two strains) show that the same strain combinations differ significantly in their fitness, as for the competitive indices in the supplementary Table S8.1.

Table S9

Strains	Appearance time/lag time [min] median w/o CAZ	Appearance time/lag time [min] median with CAZ
ECC Δ*catA2*ΔampD	528	517
ECC Δ*catA2*Δ*dacB*	514	502
ECC (WT) parental population	485	654

Table S9. Lag times of the *dacB*- and *ampD* knockout-strains compared to the *Enterobacter cloacae* complex IMT 49658 (ECC WT). On plates without CAZ and on plates with CAZ there are the same order of lag times to each other as with the *dacB*/*ampD*_ev_mutants. The *ampD* knockout strain has the largest fitness loss compared to the *dacB* knockout strain on plates with and without CAZ. All appearance times were significantly different to each other ($p < 0.0001$, Mann-Whitney-U Test).

Table S10

Strains	ECC (WT) parental population	ECC *dacB*_ev_mutant (37)	ECC *ampD*_ev_mutant (39)
MIC summary (n=3) [µg/ml]	125	1000	4000
MIC replicate 1 [µg/ml]	125	1000	4000
MIC replicate 1 [µg/ml]	125	2000	4000
MIC replicate 1 [µg/ml]	125	1000	2000

Table S10. Minimum inhibitory concentration (MIC) assays comparing the ECC (WT) parental population with the *dacB*- and *ampD* mutants. *DacB*- and, in particular, the *ampD* mutant exhibit significantly higher MIC values than the ECC (WT) parent population, which must first undergo selection for gene duplication/amplification of bla_{DHA-1} in order to confer resistance. The MIC was determined as the initial antibiotic concentration at which no increase in optical density ($OD_{600\ nm}$) was observed 20 hours after inoculation, based on three biological replicates per strain.

Table S11.1

	IMT 49658-1 (ECC) Δ*catA2*Δ *bla*$_{DHA-1}$<br>Replicate 1,2,3 and starting inoculum $5x10^7$<br>-one cycle of evolution-	ECC (WT) parental population<br>Replicate 2,3,4 at 32 µg/ml CAZ and starting inoculum 10^5 CFU/ml<br>-one cycle of evolution-
GDAs (fold change value of the ECC (WT) w/o CAZ)	Replicate 1: 1.03 GDAs<br>Replicate 2: 1.07 GDAs<br>Replicate 3: 1.07 GDAs	Replicate 2: 44.23 GDAs<br>Replicate 3: 25.57 GDAs<br>Replicate 4: 0.4 GDAs
SNPs (*dacB*/*ampD*)	Replicate 1-3: *ampD* SNP<br>CDS Pos. 497:<br>Bases: G->C<br>AS: G-A	Replicate 4: *dacB* SNP<br>CDS Pos. 253:<br>Bases: T->G<br>AS: T->P

Table S11.1. Experimental results on evolution in 32 µg/ml CAZ with the ECC (WT) parental population and its GDA-deficient counterpart. These data accompany Figure 3B in the main text. At the lowest starting cell concentration at which the ECC WT managed to rescue the population, we detected GDAs responsible for the population rescue in 32 µg/ml CAZ for two of three replicates. In the third replicate we detected reduced GDAs as a *dacB* mutant overtook the population, which is may be caused by stochasticity within the founder population. For the GDA deficient *bla*$_{DHA-1}$ mutant, we detected within one cycle of evolution no GDAs but a SNP in the *ampD* gene for all three replicates at the lowest starting cell concentration at which the mutant grew in 32 µg/ml CAZ. Since SNPs immediately appeared in the GDA-deficient mutant, we stopped the evolution after one cycle of evolution

Table S11.2

Starting inoculum [CFU/ml] at 32 µg/ml CAZ	IMT 49658-1 (ECC) Δ*catA2*Δ *bla*$_{DHA-1}$ (incapable of selecting gene-duplication amplifications (GDAs))	ECC (WT) parental population (capable of forming gene-duplication amplifications (GDAs))
10^1	No growth in 3/3 replicates	No growth in 3/3 replicates
10^3	No growth in 3/3 replicates	No growth in 3/3 replicates
10^5	No growth in 12/13 replicates	Growth in 11/13 replicates
$8x10^6$	Growth in 3/3 replicates	Growth in 3/3 replicates
$5x10^7$	Growth in 3/3 replicates	Growth in 3/3 replicates

Table S11.2. Experimental results for gene-duplication amplification (GDA)-mediated population rescue. These data are shown in Fig. 3C in the main text. In summary, at high antibiotic concentrations the ECC (WT) parental population manages to grow and rescue the population at starting concentrations of 10^5 CFU/ml. A population rescue for the *bla*$_{DHA-1}$ deletion mutant, incapable of selecting GDAs, is only possible with a population size > 10 fold higher (starting concentration 8x10^^{6} CFU/ml).

Table S12

Parameter	Parameter value	Parameter description	Literature value?	Source?
gda_benefit	3	Capacity of antibiotic degradation per GDA (effect on IC50; Eq. 3)		Estimated based on ability of the model to reproduce avg. GDA at different CAZ (Fig. S11A)
gda_cost	10^{-3}	Fitness cost per GDA		Estimated from competition assay (10^{-4} – 10^{-3}), same range as Pettersson *et al.* [2]/Pal *et al.* [3]
k_dup	6×10^{-4}	Rate of initial duplication event		Estimate from range in Pettersson *et al.*[4]
k_rec	0.2	Intrinsic probability of recombination between two GDAs		Estimate, Sun *et al.* [5]
min_dr	-0.1	Lower asymptote of dose-response curve		Estimated based on ability of the model to reproduce avg. GDA at different CAZ (Fig. S11A)
max_gdas	300	Maximal allowed number of GDAs		Arbitrary
p	8	Hill coefficient of dose-response curve		Estimated based on ability of the model to reproduce avg. GDA at different CAZ (Fig. S11A)
mumax [hr^{-1}]	1.7	Maximal growth-rate of ECC (WT) parental population		Experimental value
K_m	0.25	Monod constant		Estimate, Nordholt *et al.* [6]
e	5×10^{-9}	Inverse yield constant		Experimental estimate based on final cell density
R [mM]	40	Initial resource concentration		Estimate, Nordholt *et al.* [6]
mr_dacB	$2 \times 10^{-8} \times (1434/(564+1434)$	Rate of emergence of *dacB* (scaled by gene length)		Kohlmann *et al.* [7]

mr_ampD	$2 \times 10^{-8} \times (564/(564+1434)$	Rate of emergence of *ampD (*scaled by gene length)		Kohlmann *et al.* [7]
ic50_dacB [µg/ml]	750	IC50 of ECC *dacB*_ev_mutant (37)		Calculated from Table S10, Eq. (2) (MIC: 1 mg/ml ceftazidime (CAZ))
ic50_ampD [µg/ml]	3000	IC50 of ECC *ampD*_ev_mutant (39)		Calculated from Table S10, Eq. (2) (MIC: 4 mg/ml ceftazidime (CAZ))
cost_dacB	0.008	Fitness cost of *dacB* mutation		Experiment (Fig. 1 D, E)
cost_ampD	0.175	Fitness cost of *ampD* mutation		Experiment (Fig. 1 D, E)

Table S12. Model parameters for the deterministic modelling of evolutionary dynamics in *Enterobacter cloacae* complex IMT 49658.

Table S13

	Mutation rate μ (per generation)	Lower 95% CI	Upper 95% CI	*p* (ECC (WT) vs. GDA deficient strain)
ECC (WT) parental population	3.09E-10	1.55E-10	4.63E-10	*p* = 0.3292 (two sample t-test)
ECC Δ*catA2*Δ bla_{DHA-1} (GDA deficient strain)	2.29E-10	9.26E-11	3.65E-10	

Table S13. Comparison of mutation rates in the ECC (WT) parental population and the GDA deficient counterpart. Based on fluctuation assays using nalidixic acid as the mutation rate marker, we demonstrate that the mutation rate μ is not altered due to the ability to form gene duplication-amplifications (GDA). The mutation rates of the ECC (WT) parental population (ability to form GDA) and the ECC with the deletion of bla_{DHA-1} (GDA deficient) were similar and unsignificant ($p = 0.3292$).

Figure S1

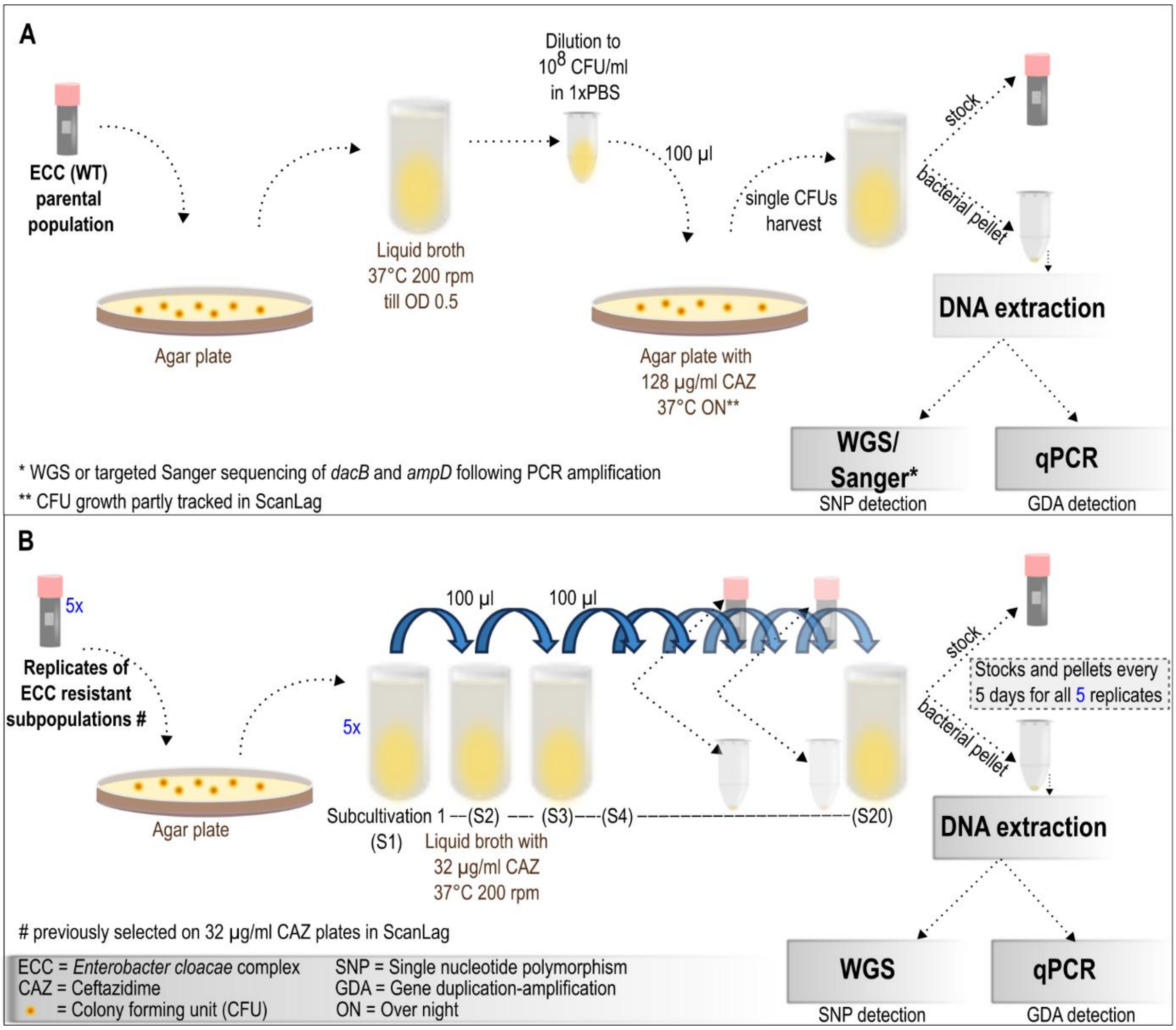

Figure S1. Schematic overview of the experimental design for the evolution under A) high ceftazidime (CAZ) pressure on agar plates and B) under long-term CAZ pressure in liquid broth. This graphic and all elements within were generated by the authors using Inkscape vector graphics editor.

Figure S2

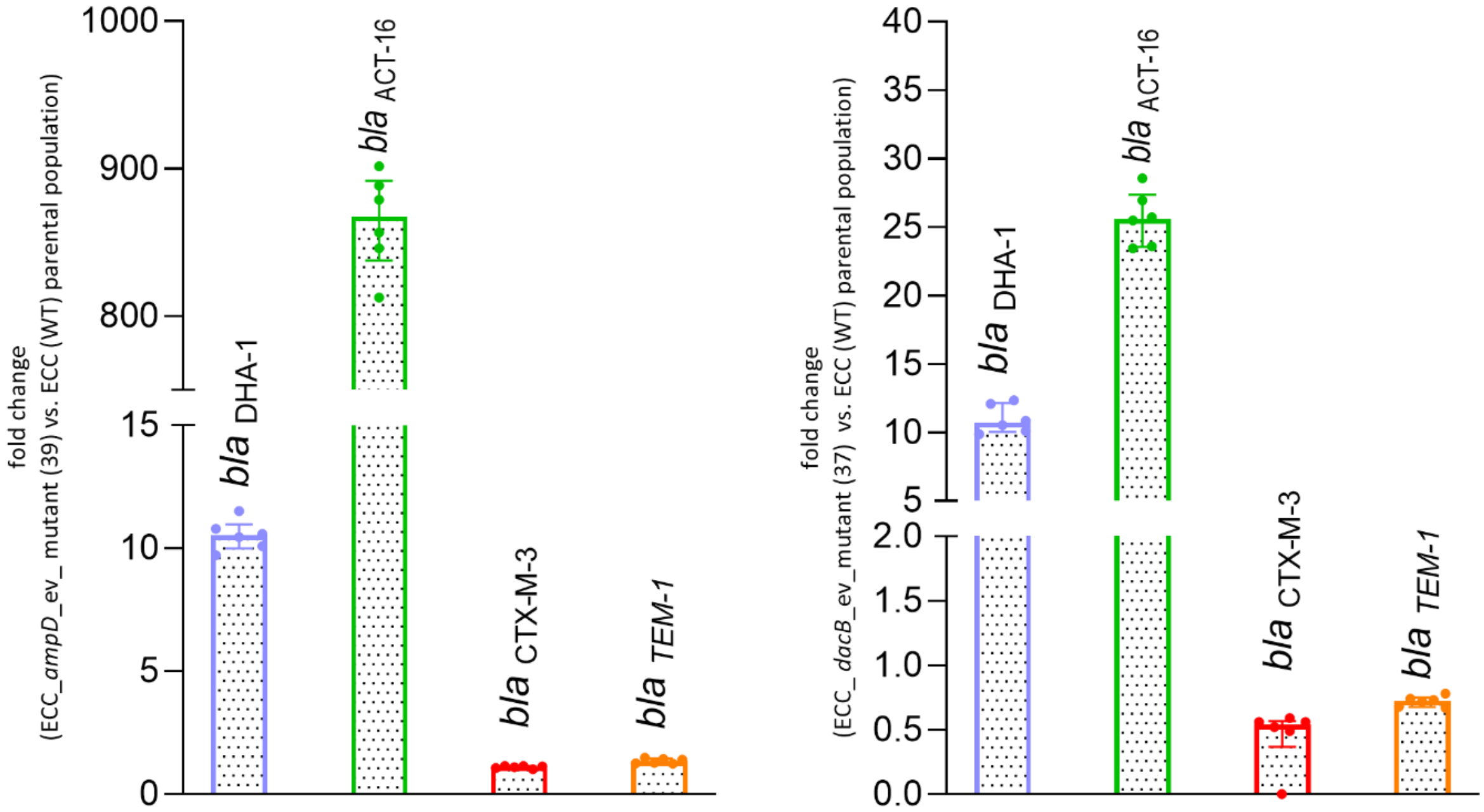

Figure S2. Fold change β-lactamases gene expression in *dacB*- and *ampD_ev*_mutants. Fold change gene expression of all four β-lactamases of the *Enterobacter cloacae* complex ECC_*ampD*_ev_mutant (39) (left) and ECC_*dacB*_ev_mutant (37) (right) compared to the ECC (WT) parental population, grown to exponential phase without ceftazidime (CAZ). *DacB*- and *ampD* mutants show ampC derepression with high expression of the chromosomal ampC β-lactamases bla_{ACT-16} and to a lesser extend the plasmid borne ampC bla_{DHA-1} that is upregulated in the gene duplication-amplification driven resistant subpopulations of the heteroresistant ECC (WT) parental population in CAZ pressure (Referenz mein manuscript 1). Class A β-lactamases bla_{CTX} and bla_{TEM-1} are not upregulated in the ECC_*ampD- and dacB*_ev_mutant. Results from cDNA are plotted with median and IQR of n=6 replicates per gene.

Figure S3

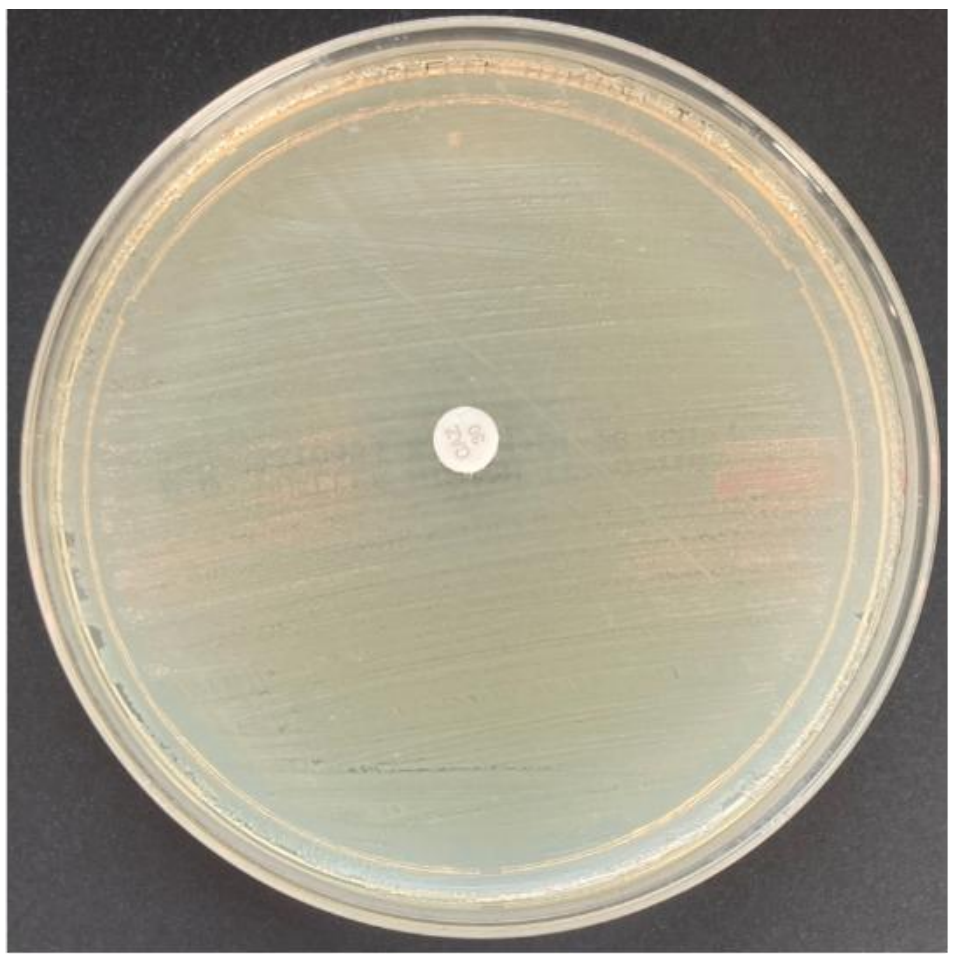

IMT 49658-1 (ECC) Δ*catA2*Δ*dacB*

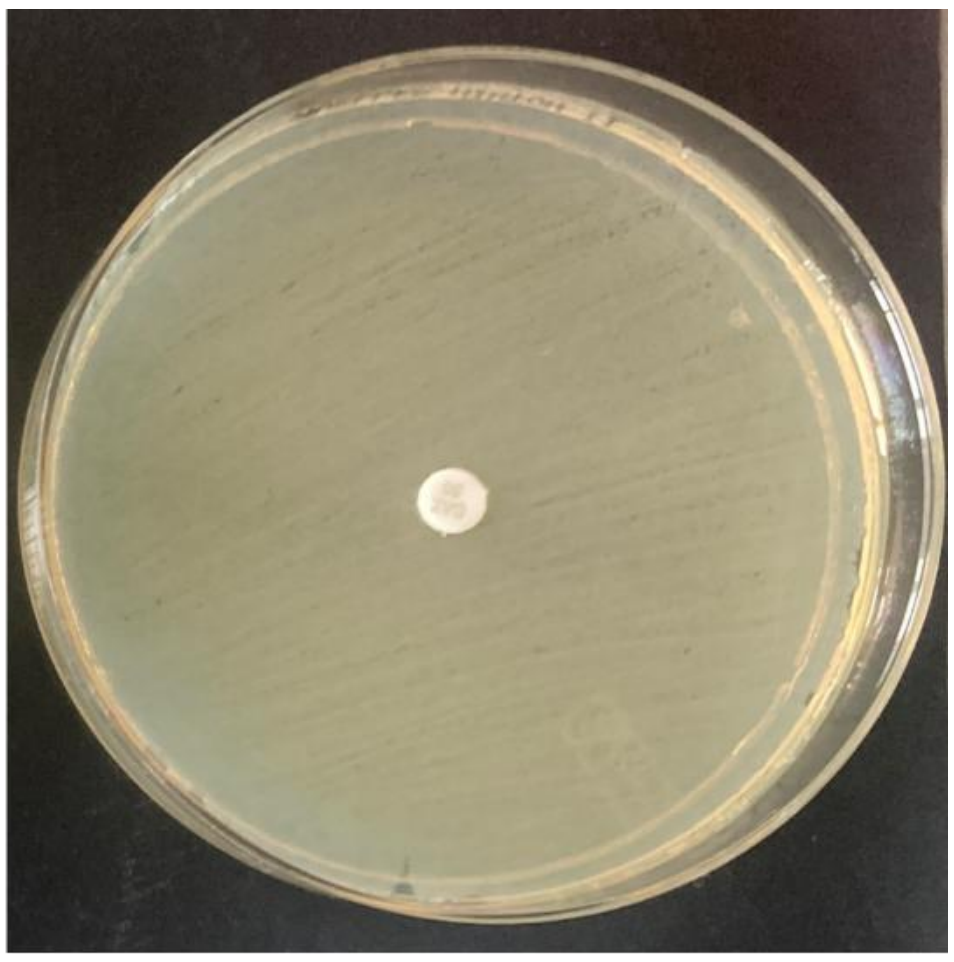

IMT 49658-1 (ECC) Δ*catA2*Δ*ampD*

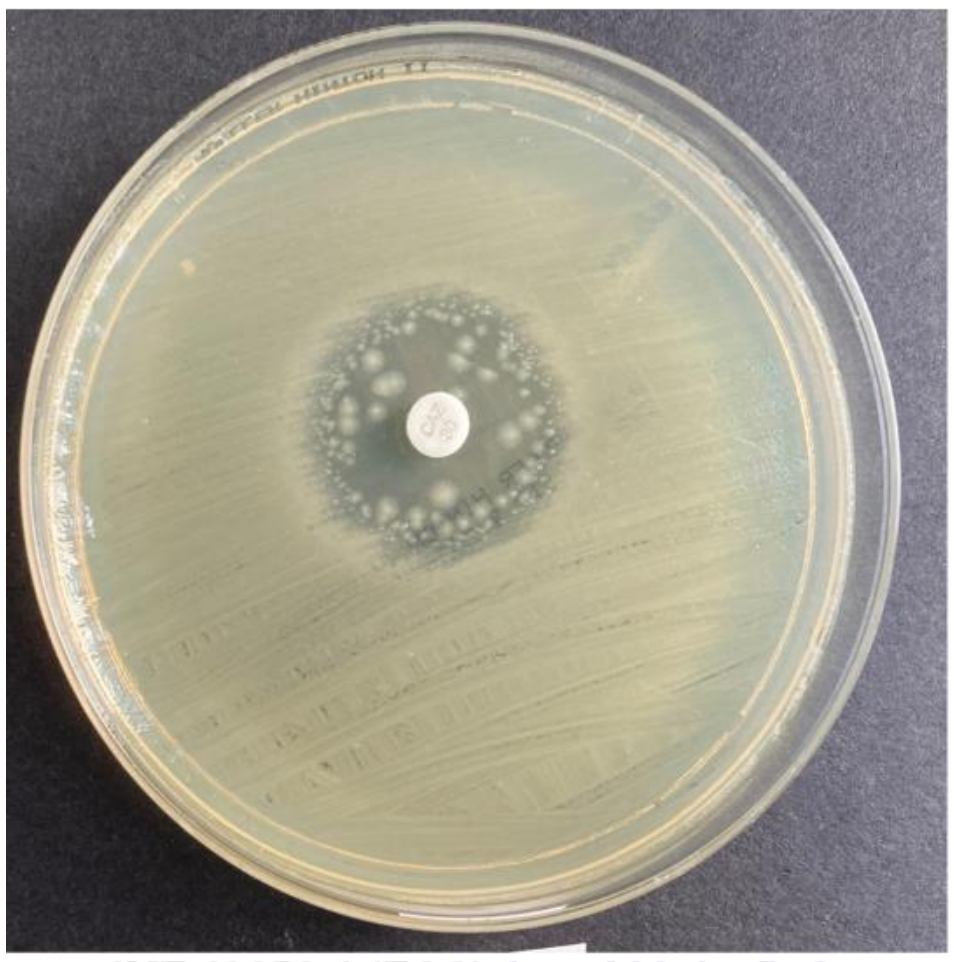

IMT 49658-1 (ECC) Δ*catA2*Δ*dacB*, C

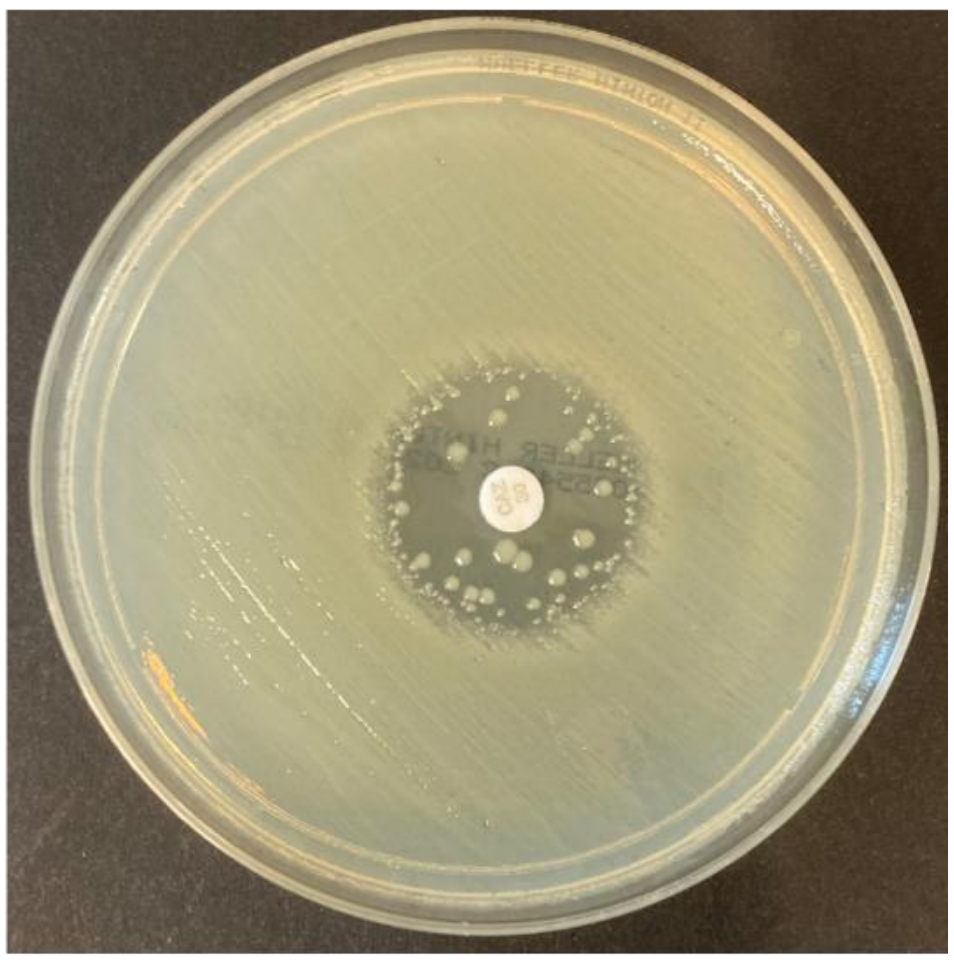

IMT 49658-1 (ECC) Δ*catA2*Δ*ampD*, C

Figure S3. IMT 49658-1 (ECC) Δ*catA2*Δ*dacB* and IMT 49658-1 (ECC) Δ*catA2*Δ*ampD* and their corresponding complemented strains in agar-disk diffusion. IMT 49658-1 (ECC) Δ*catA2*Δ*dacB* and IMT 49658-1 (ECC) Δ*catA2*Δ*ampD* show a resistant phenotype in agar-disk diffusion with CAZ. When pACYC177_XbaI_*dacB/ampD* is complemented in the corresponding knockout-strain, a restoration of the heteroresistant phenotype is visible. Complementation is annotated with a "C".

Figure S4

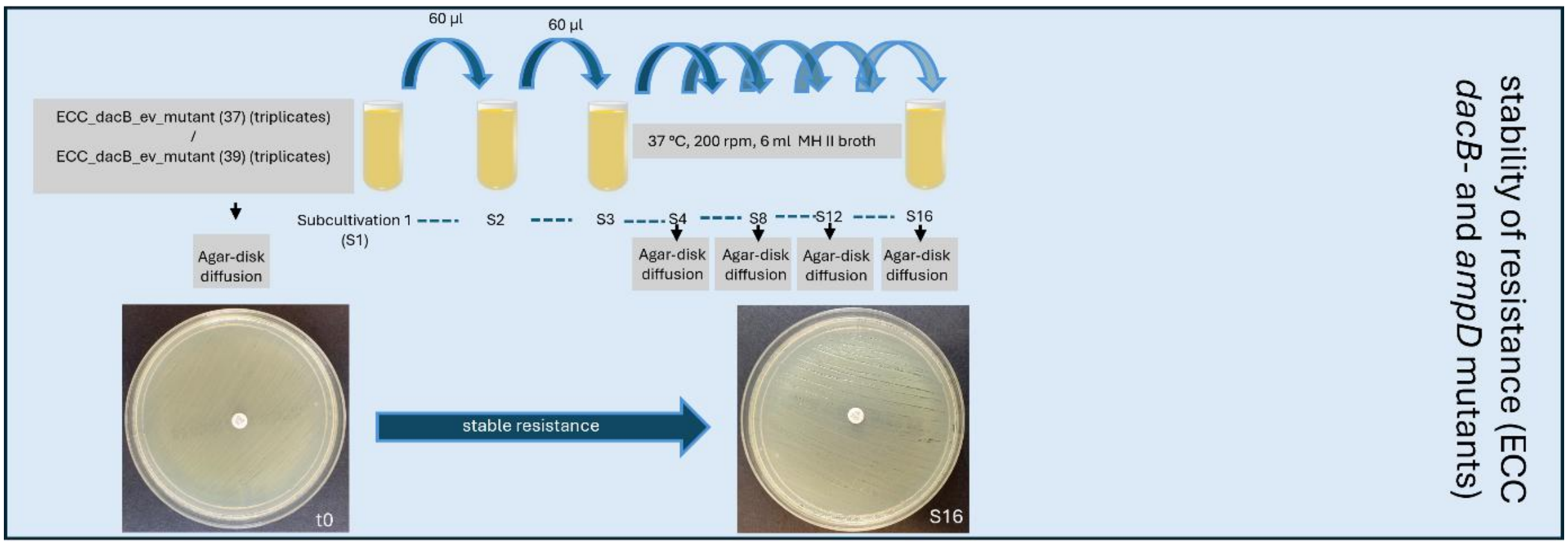

Figure S4. Stability of resistance in evolved ECC *dacB*- and *ampD*_ev_mutants. The ECC *dacB* mutant (ECC_*dacB*_ev_mutant (37)) and ECC *ampD* mutant (ECC_*ampD*_ev_mutant (39)), originating from single-step evolution on plates with 128 µg/ml CAZ, showed stable resistance in agar-disk diffusion assays when grown in MH II broth without antibiotics (CAZ) for S16 subcultivations (n=3 replicates per mutant). Subcultivations took place every 2.5 hours to maintain exponentially growing bacteria to maximize bacterial generations and thus opportunity for resistance reversion.

Figure S5

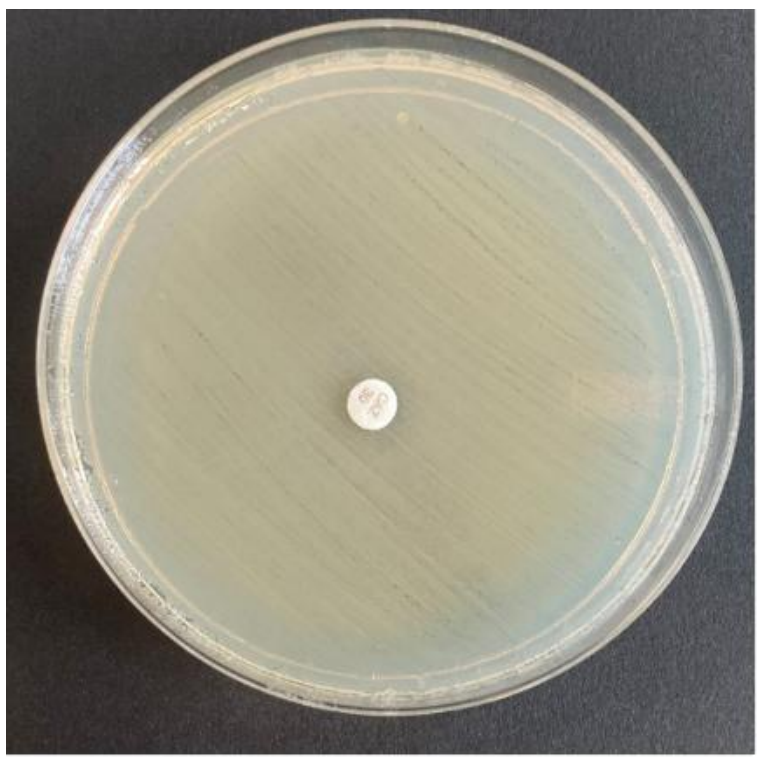

ECC *dacB*_ev_mutant(7)

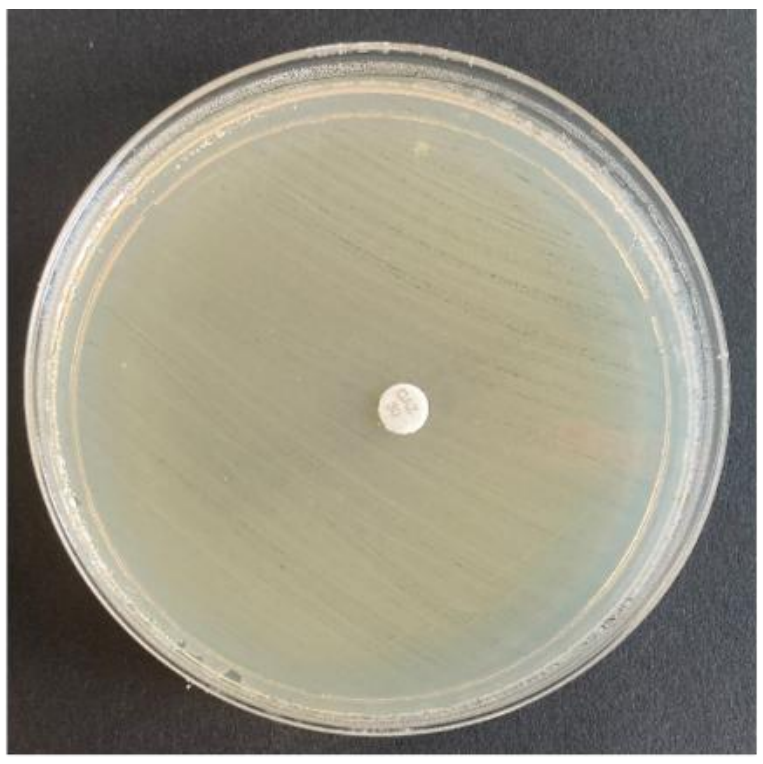

ECC *dacB*_ev_mutant (37)

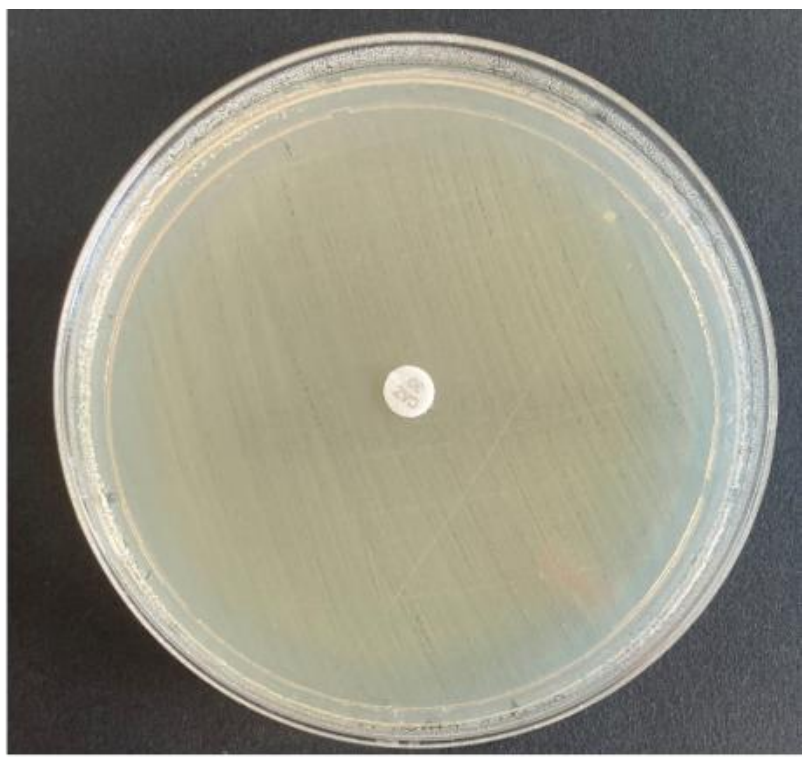

IMT 49658-1 (10) ev20

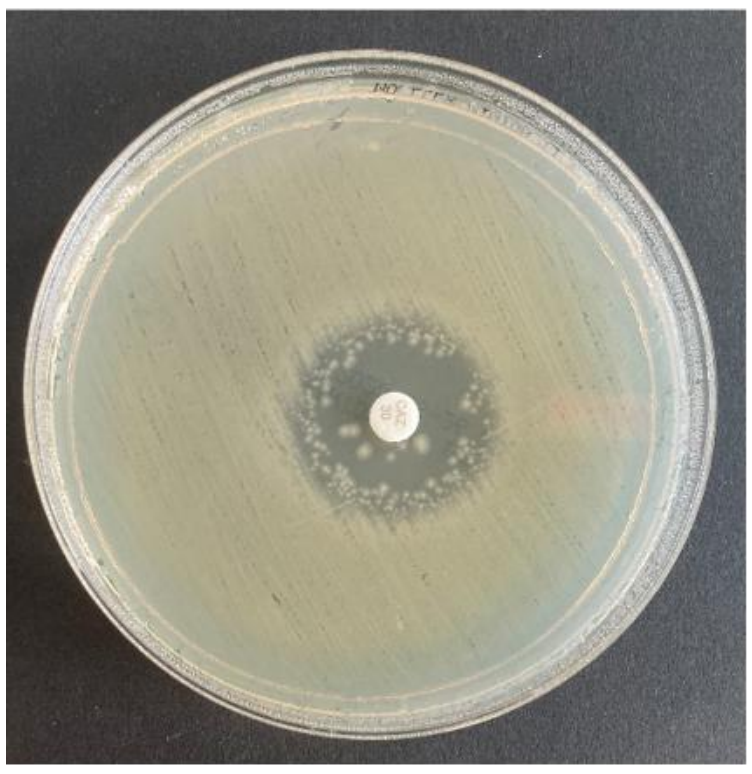

ECC *dacB*_ev_mutant(7), C

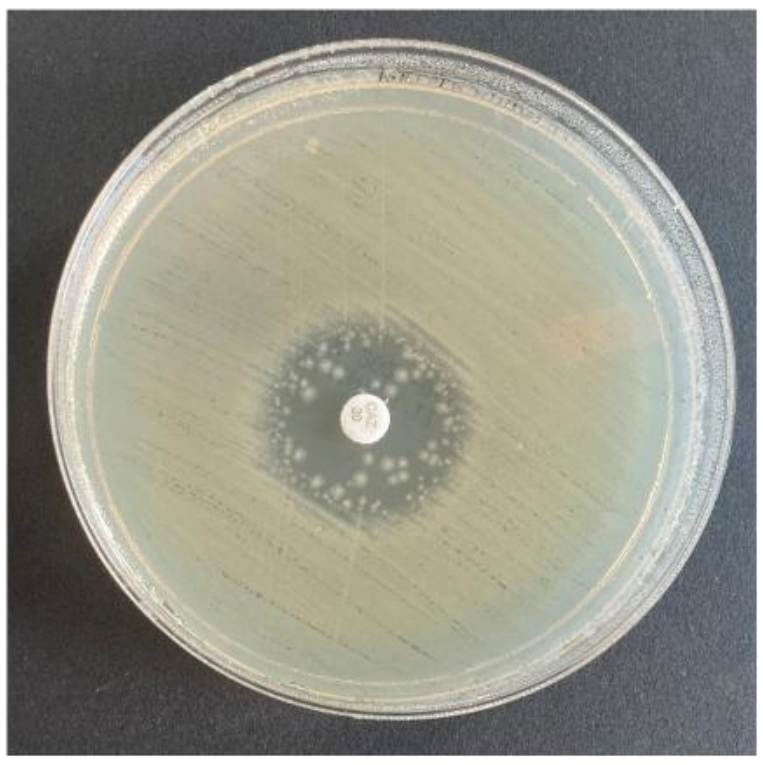

ECC *dacB*_ev_mutant (37), C

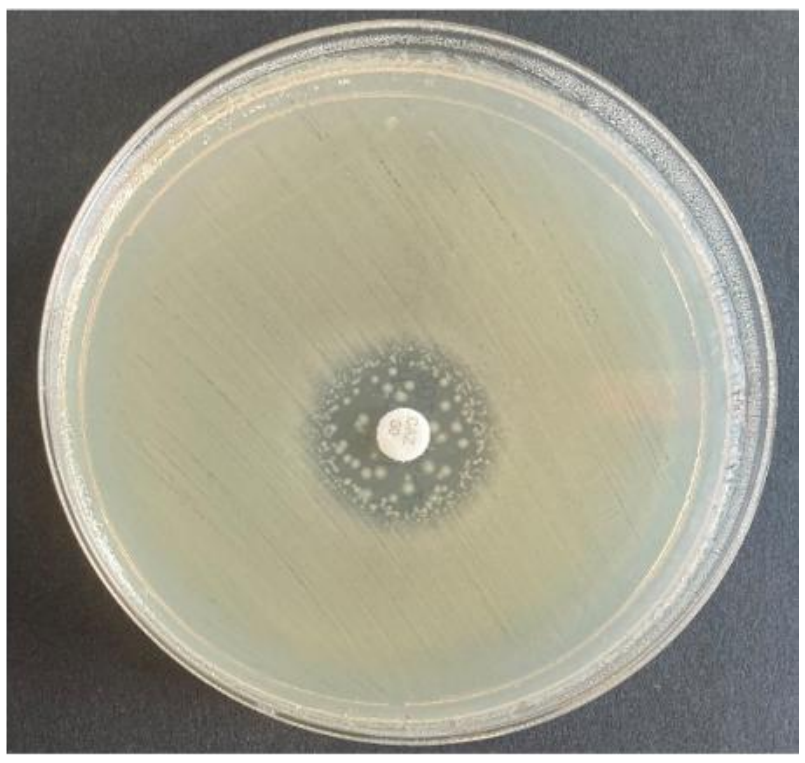

IMT 49658-1 (10) ev20, C

Figure S5. IMT 49658-1 (ECC) *dacB*_ev_mutants and their corresponding complemented strains in agar-disk diffusion. IMT 49658-1 (ECC) *dacB*_ev_mutant (7), (37), (10)ev20 grow with a resistant phenotype on MH II plates, when agar-disk diffusion with CAZ is performed. When pACYC177_XbaI_*dacB* is complemented in the corresponding knockout-strain, a restoration of the heteroresistant phenotype is visible. Complementation is annotated with a “C”.

Figure S6

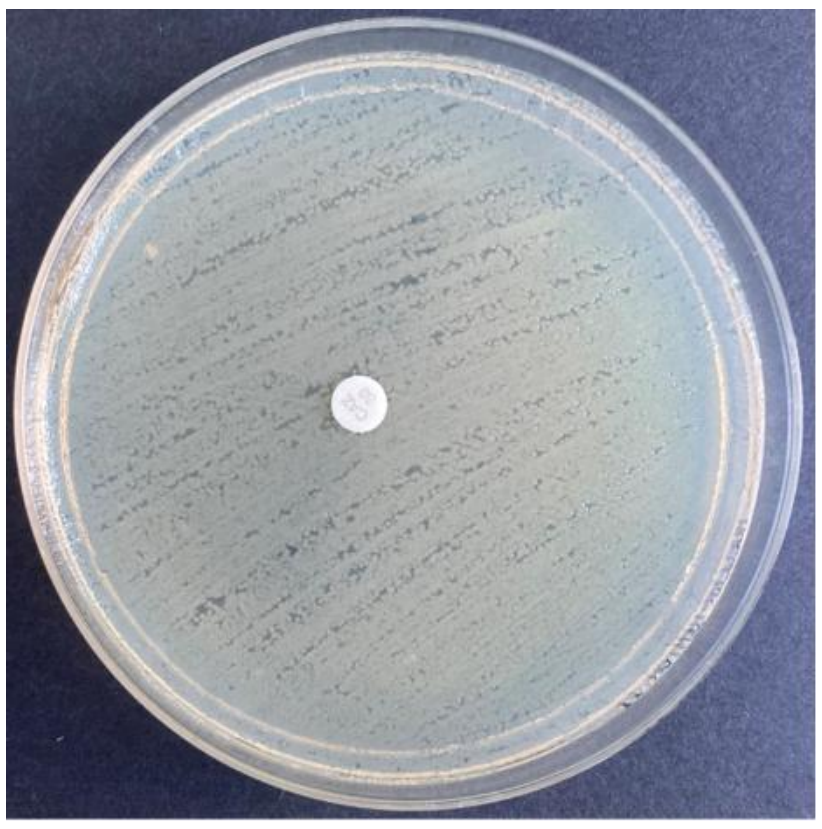

ECC *ampD*_ev_mutant (39)

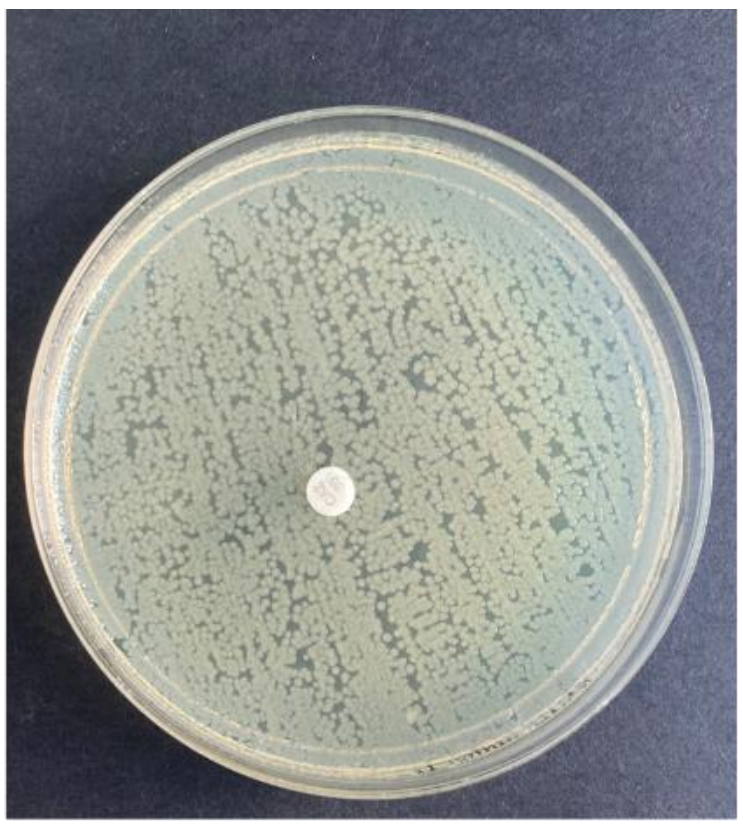

ECC *ampD*_ev_mutant (153)

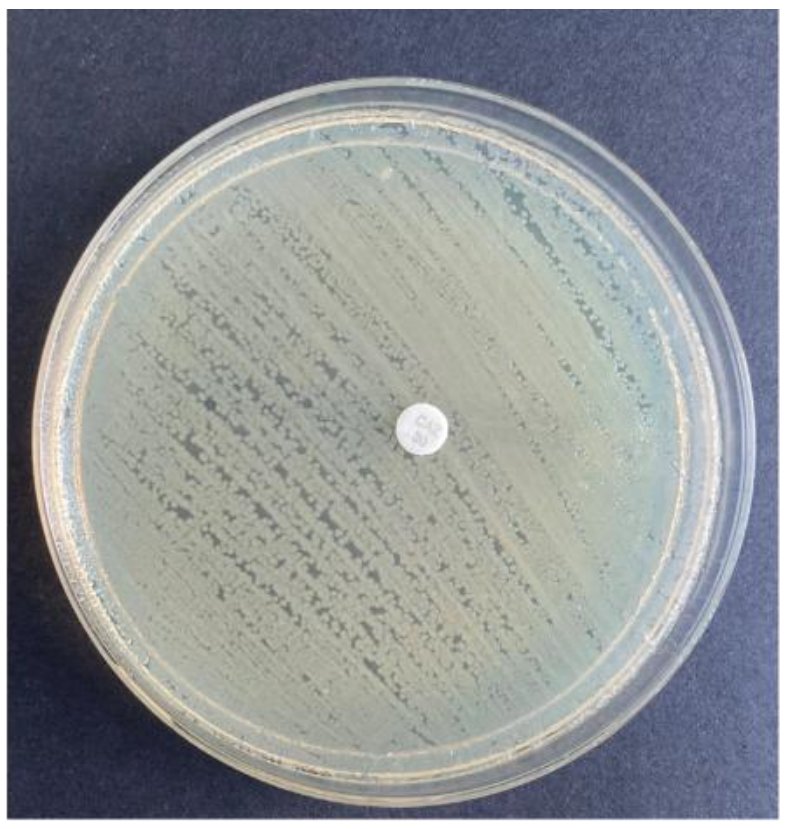

ECC *ampD*_ev_mutant (163)

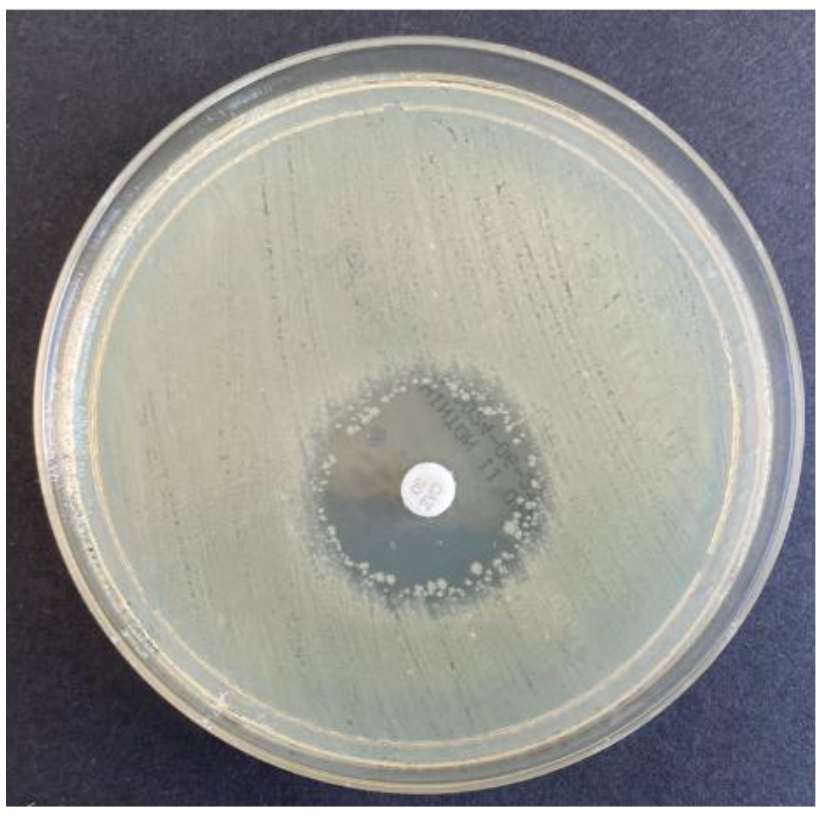

ECC *ampD*_ev_mutant (39), C

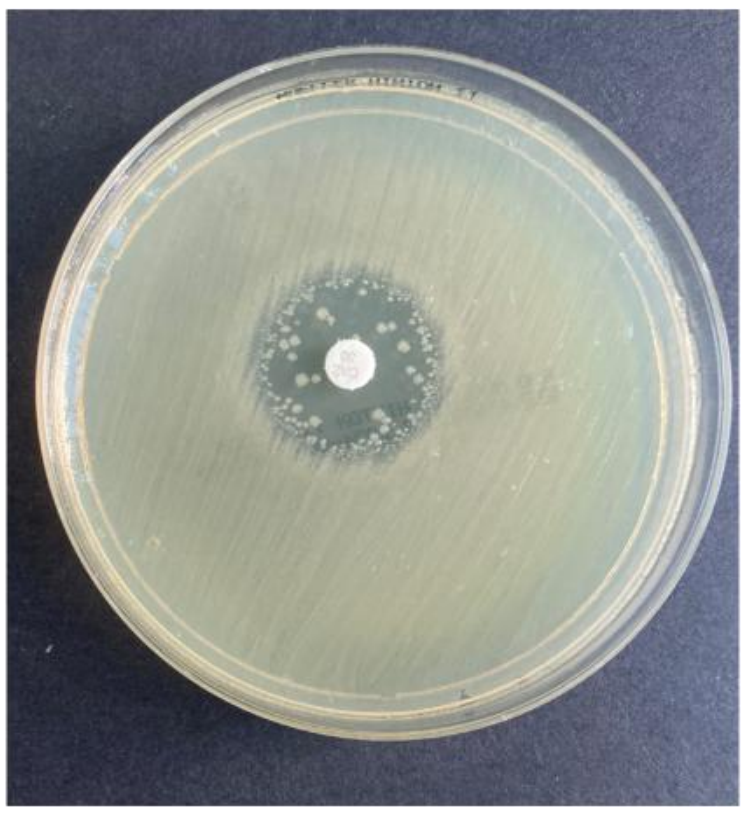

ECC *ampD*_ev_mutant (153), C

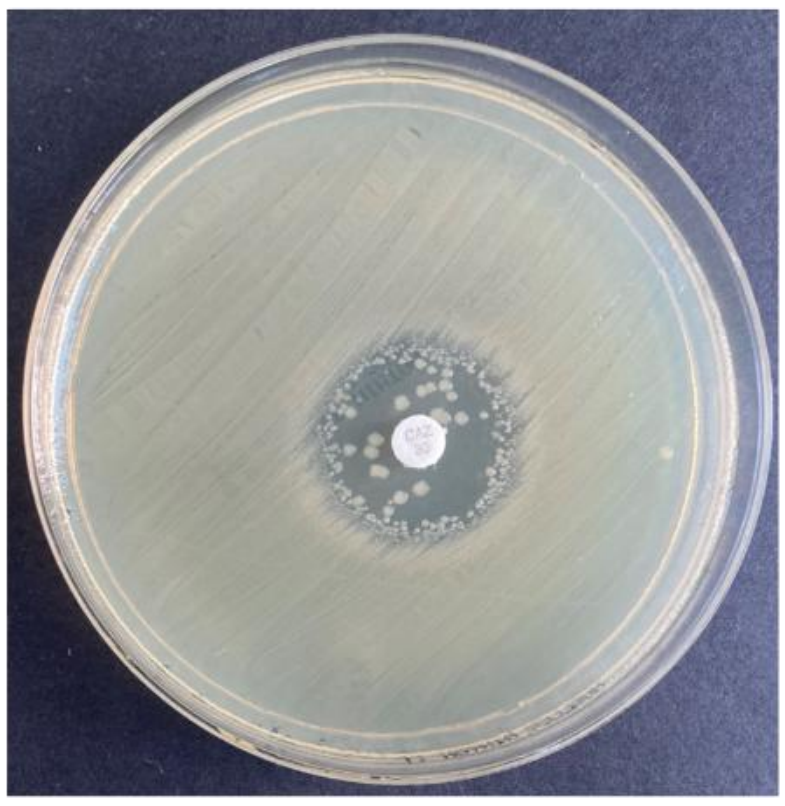

ECC *ampD*_ev_mutant (163), C

Figure S6. 3 IMT 49658-1 (ECC) *ampD_ev_mutants* and their corresponding complemented strains in agar-disk diffusion. IMT 49658-1 (ECC) *ampD*_ev_mutant (39), (153), (163) display a resistant phenotype on agar-disk diffusion with CAZ. When pACYC177_XbaI_*ampD* is complemented in the corresponding knockout-strain, a restoration of the heteroresistant phenotype is visible. Complementation is annotated with a “C”.

Figure S7.1

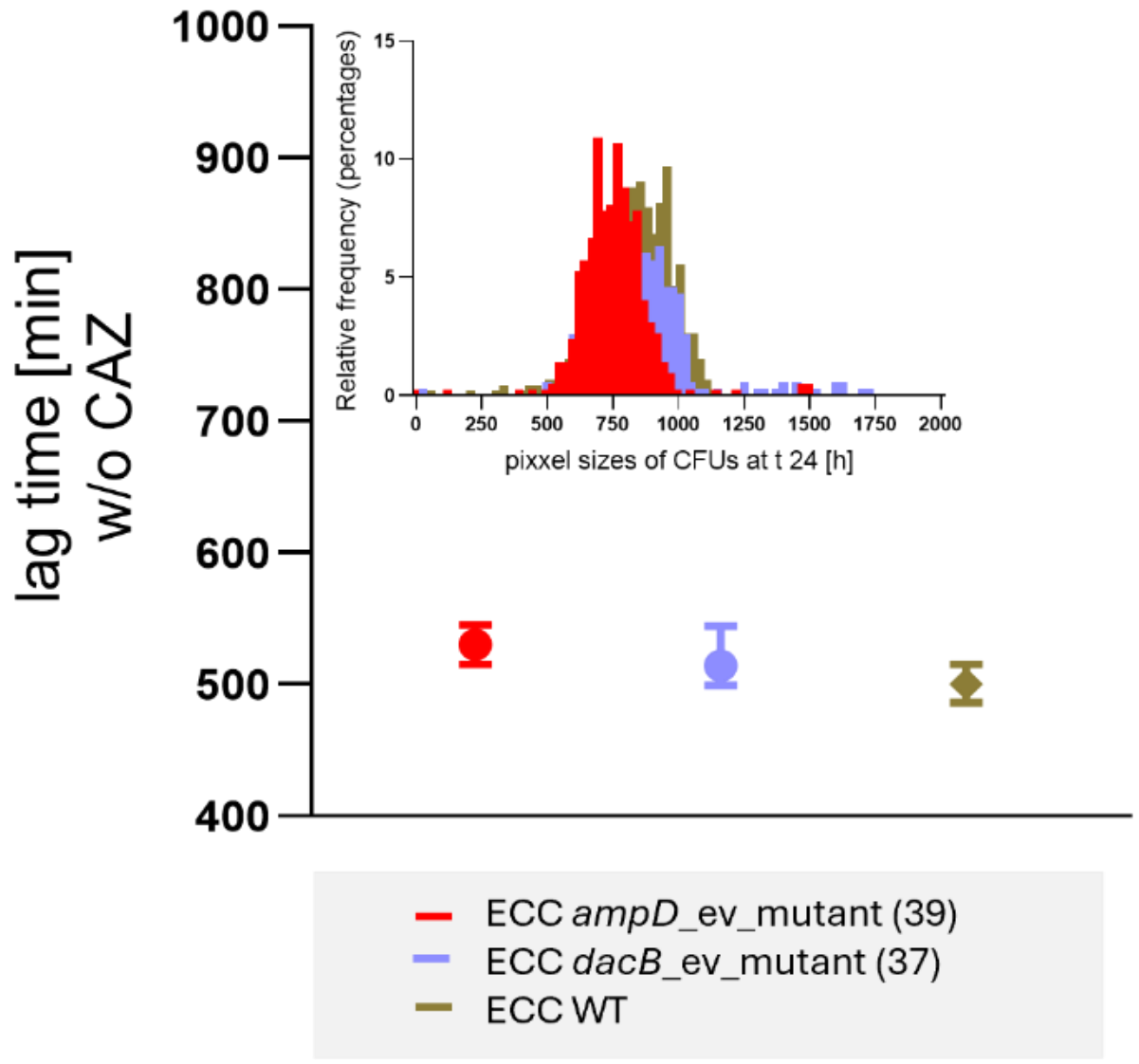

Figure S7.1. Lag times and colony size distribution at t 24 hours of ECC *dacB* and *ampD*_ev_mutants in ScanLag without CAZ. The strongest fitness loss was observed for the *ampD* mutant (ECC_*ampD*_ev_mutant (39)) with a median lag time of 530 min. followed by the *dacB* mutant with 514 min. which lag times were only ten min. longer than these of the ECC WT (504 min.) (Supplementary table 7). The appearance time of the *dacB* mutant was significantly different to that of the *ampD* mutant, as well as the *ampD*- and *dacB* mutant each to the ECC WT (for all combinations $p < 0.0001$, Mann-Whitney-U Test). The colony size distribution of the *ampD* mutant was the smallest with a median colony size of 758 pixels, followed by the *dacB* mutant with 826.5 pixels and the ECC WT parental population with 866 pixels. Colony sizes between these three groups were significantly different ($p < 0.0001$, Kruskal-Wallis Test).

Figure S7.2

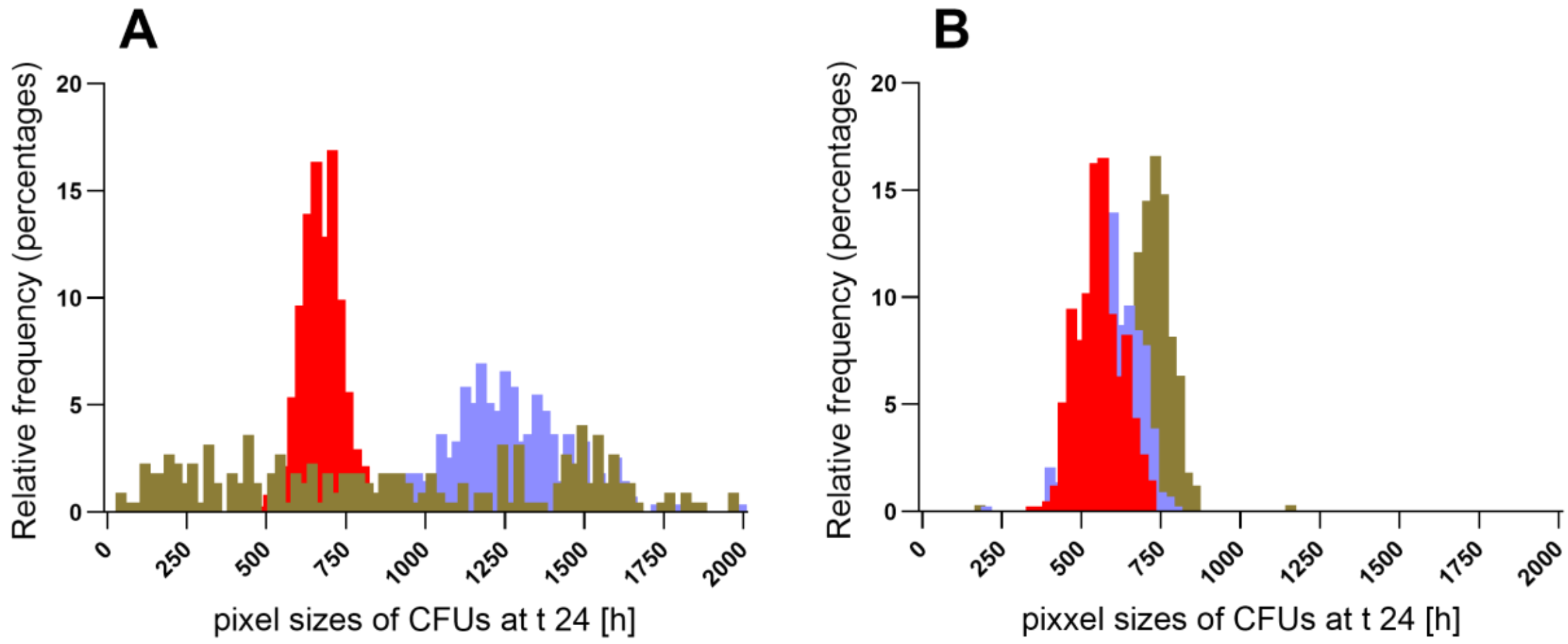

Figure S7.2 Distribution of colony sizes of *dacB*- and *ampD* knockout strains and ECC (WT) parental population at time point t24 in ScanLag. Y-axis represents the distribution of the relative frequency of colony sizes which are shown on the the x-axis.

A. Colony sizes (pixel) of the ECC *ampD* knockout strain population (red) are smaller compared to the ECC *dacB* knockout strain population (light blue) on plates with 16 µg/ml CAZ plates. The ECC WT source population (beige) shows wide heterogeneity in colony sizes (varying lag times) of GDA driven resistant subpopulations of the ECC source population. **B**. Colony sizes (pixel) of the ECC *ampD* knockout strain population are smaller compared to ECC source population and ECC *dacB* knockout strain population on plates w/o CAZ. Colony sizes between the ECC (WT parental population, the *dacB*- and *ampD*-knockout strain were significantly different on both, plates with and without ceftazidime (CAZ) ($p < 0.0001$, Kruskal-Wallis Test). Results in Supplementary Fig. 6.2 A & B match with the results of ECC *dacB*/*ampD*_ev_mutant shown in Supplementary Fig. 6.1 (inset) and Fig. 2 E of the main manuscript.

Figure S8

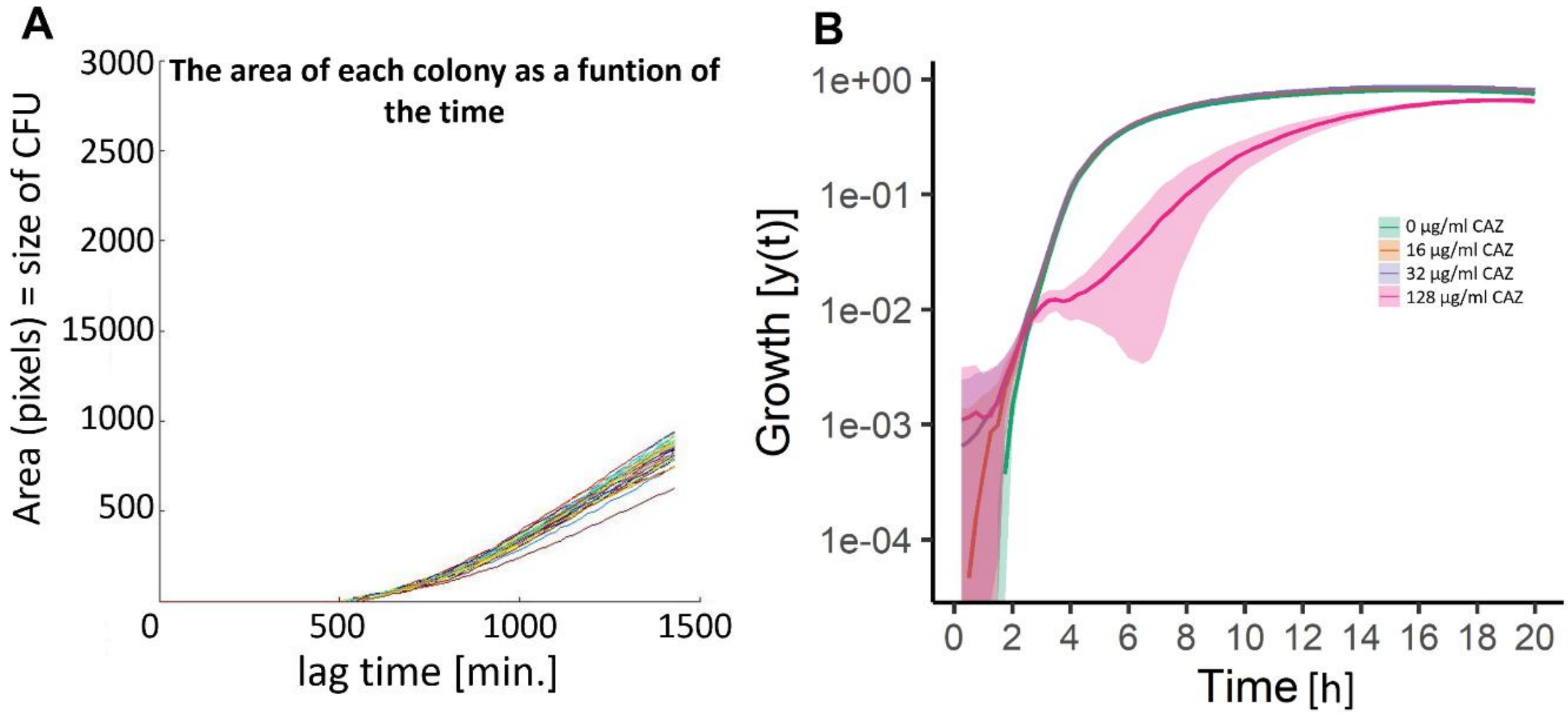

Figure S8. Loss of heterogeneity in the IMT 49658-1 (ECC) *ampD*_ev_mutant. **(A)** The area of each colony as a function of time for the evolved ECC *ampD_ev*_mutant (39) with reduced GDAs on a plate with 16 µg/ml CAZ in ScanLag. Lag times homogenize in the evolved ECC *ampD* mutant compared to resistant subpopulations of the ECC (WT) parental population (see Fig. 2C left panel of the main manuscript for the ECC *dacB* mutant). Each line represents one colony that grows over time (x-axis) and increases in size (Area in pixels on y-axis). (**B)** Growth-curve data (OD_{600} y-axis) within 20 hours (x-axis) of the evolved ECC *ampD* mutant. The evolved ECC *ampD* mutant shows a homogeneous, almost identical growth in all CAZ concentrations (0 µg/ml CAZ (green), 16 µg/ml CAZ (orange), 32 µg/ml CAZ (light blue) and 128 µg/ml CAZ (light red)). This contrasts with the ECC (WT) parental population that reveals resistant subpopulations with heterogeneous growth in rising CAZ concentrations (see Fig. 2B left panel, of the main manuscript). Graphs show the result of 3 biological replicates with each n=3 technical replicates.

Figure S9

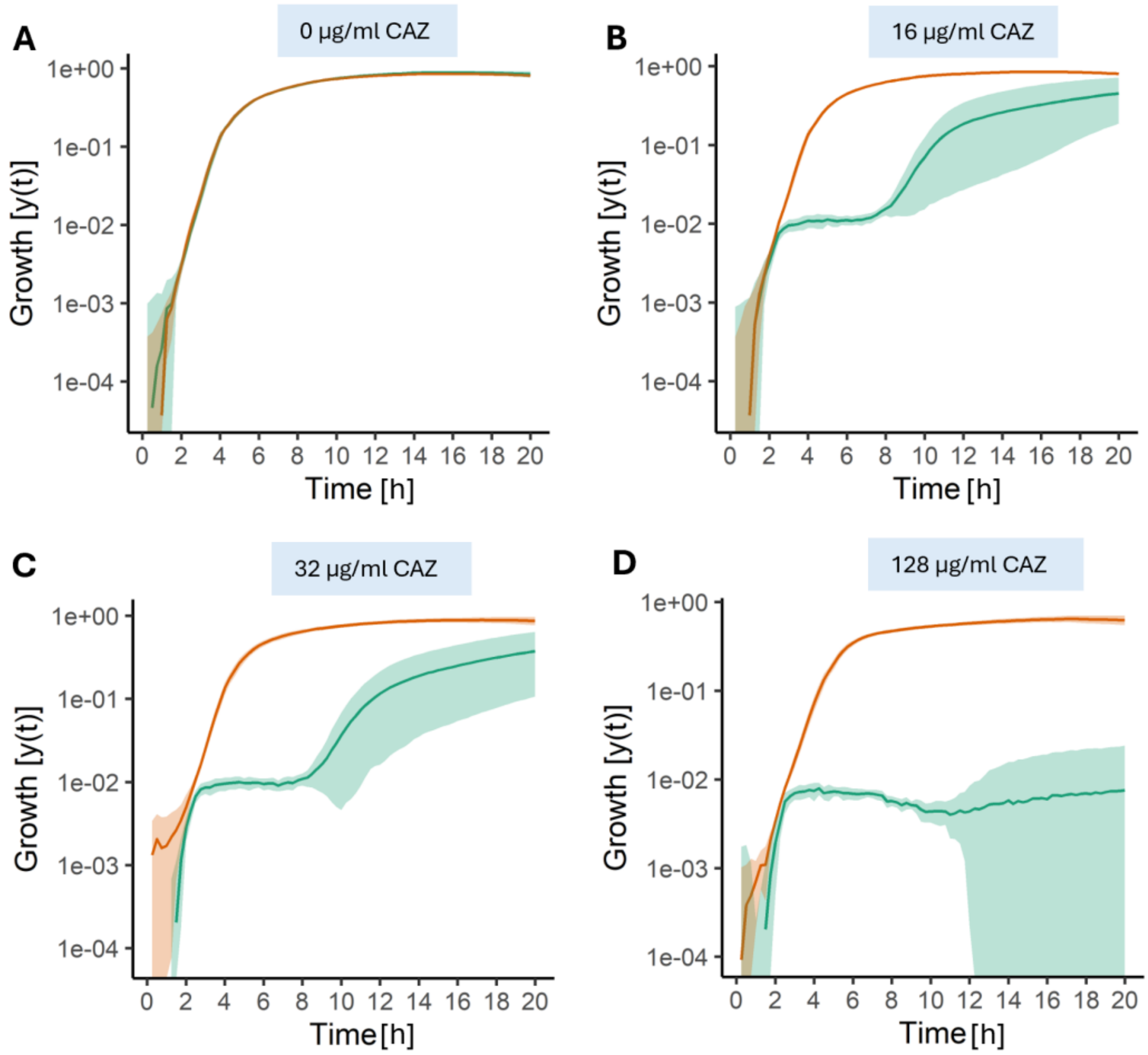

Figure S9. Comparative growth of the ECC (WT) parental population and the ECC *dacB*_ev_mutant in different ceftazidime (CAZ) concentrations. With increasing antibiotic concentrations, ascending from panels A-D, the *dacB*_ev_mutant (37) (orange) maintains stable, homogeneous growth, while the ECC (WT) parent population (green) exhibits an increasingly heterogeneous phenotype based on the gene duplication-amplification (GDA) of the AmpC-β-lactamase gene bla_{DHA-1} in resistant subpopulations. The OD600 growth curve data (y-axis) is shown over time (x-axis) from three biological replicates of each strain.

Figure S10

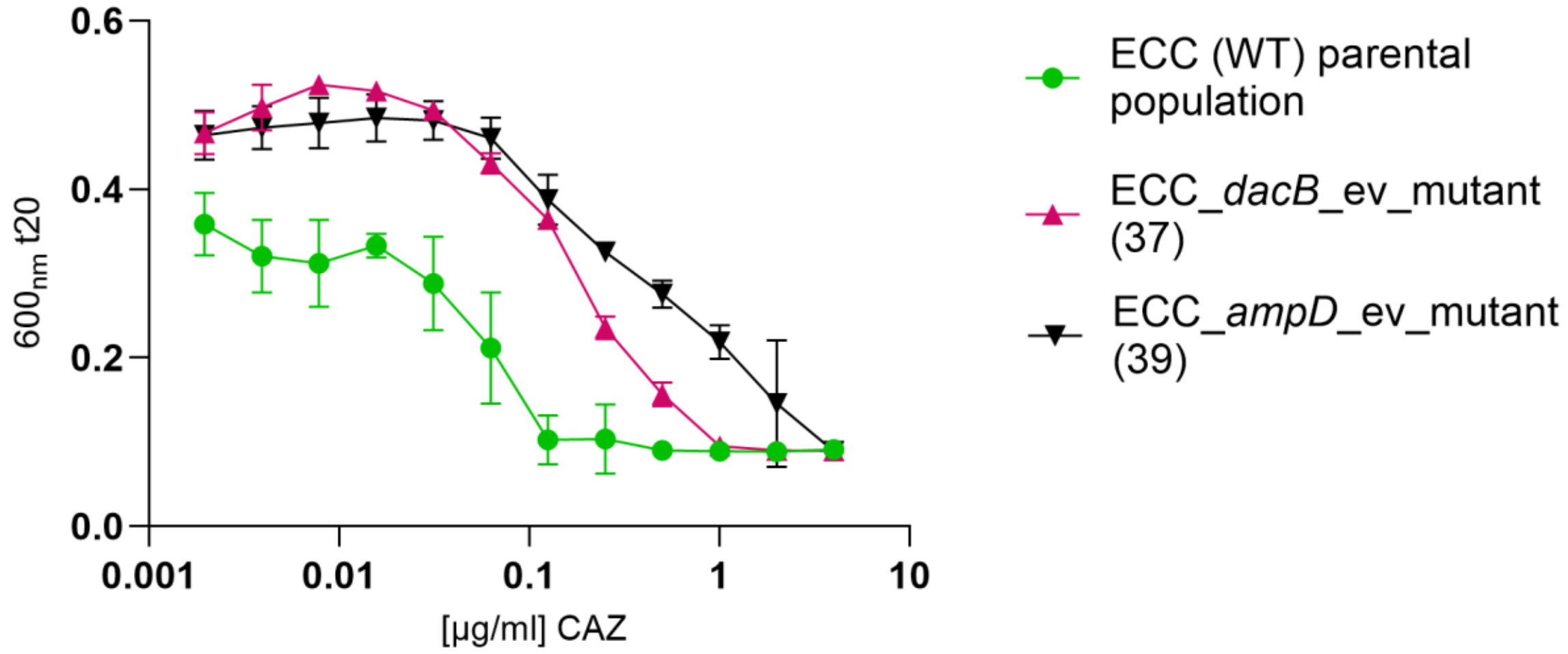

Figure S10. Growth inhibition as a function of antibiotic concentration (MIC Assay) of the ECC (WT) parental population and the ECC *dacB* – and *ampD*_ev_mutant. Bacterial growth inhibition measured by optical density (Y-axis) across concentrations of ceftazidime (CAZ) (X-axis) demonstrate a growth advantage of the *dacB*- and *ampD* mutant over the ECC (WT) parental population which must first undergo selection for gene duplication-amplification (GDA). The corresponding MIC values can be found in Table S10.

Figure S11.

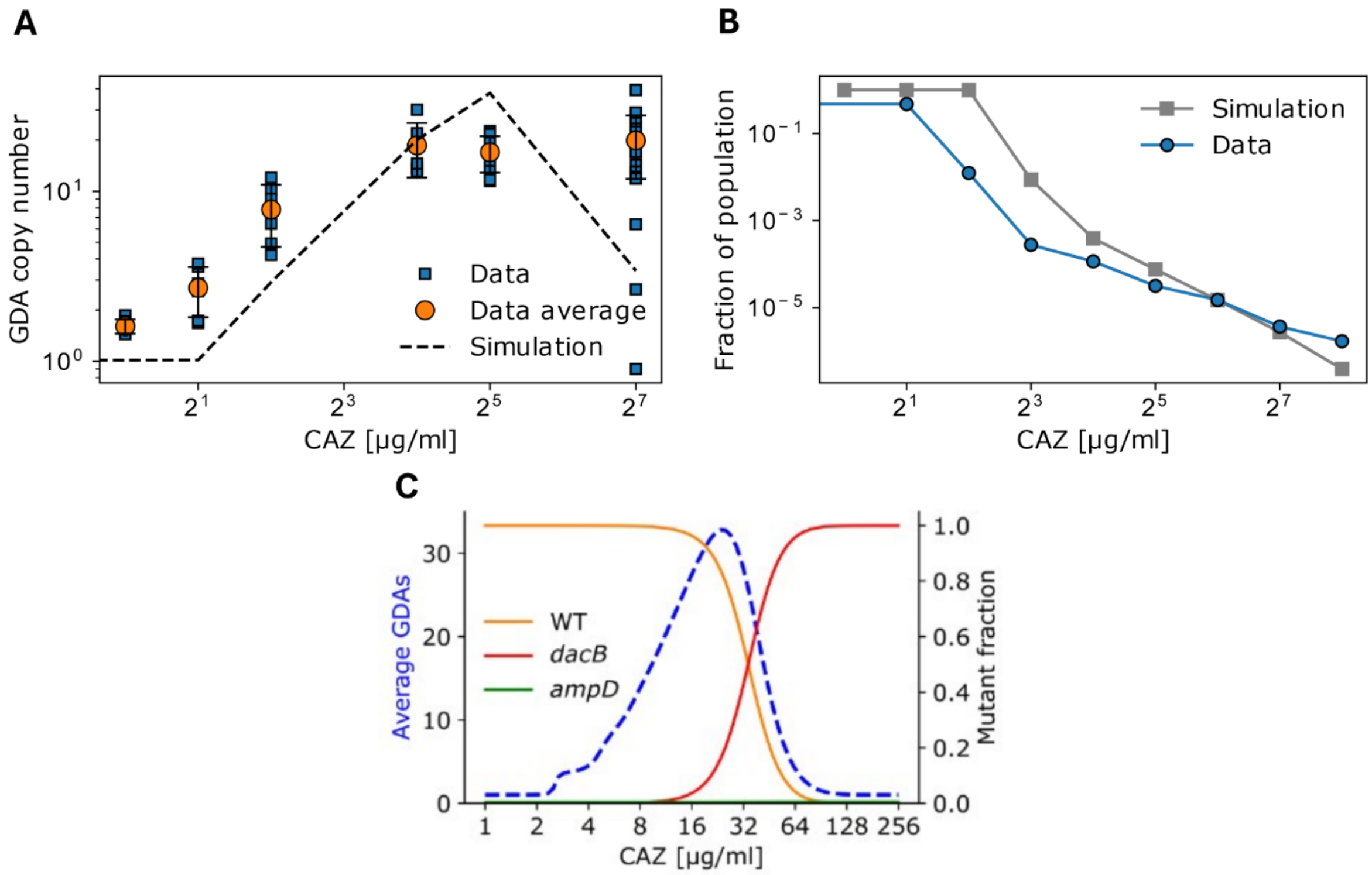

Figure S11. Model calibration and analysis. (A) Model parameters that were not determined experimentally were estimated based on the ability of the model to reproduce the GDA number per CAZ concentration from Figure 1A of the main manuscript and supplementary table 3.2 & 3.4. of the main manuscript (see Supplementary table 11 for all model parameters). (B) With these parameters, the model reproduces the features of a Population Analysis Profile (PAP)-assay performed previously [1], in which gene duplication-amplification (GDA)-driven resistant subpopulations grow on double rising ceftazidime (CAZ) concentrations. (C**)** Model predictions of average GDA copy number and mutant fraction (right axis) following propagation in different CAZ concentrations for 5 cycles. Average GDA copy number (blue dashed line, left axis) changes in a dose-dependent manner until stable resistance mutations (*dacB*, red; *ampD*; green) dominate the population at high CAZ concentrations and GDA copy number declines.

Figure S12.

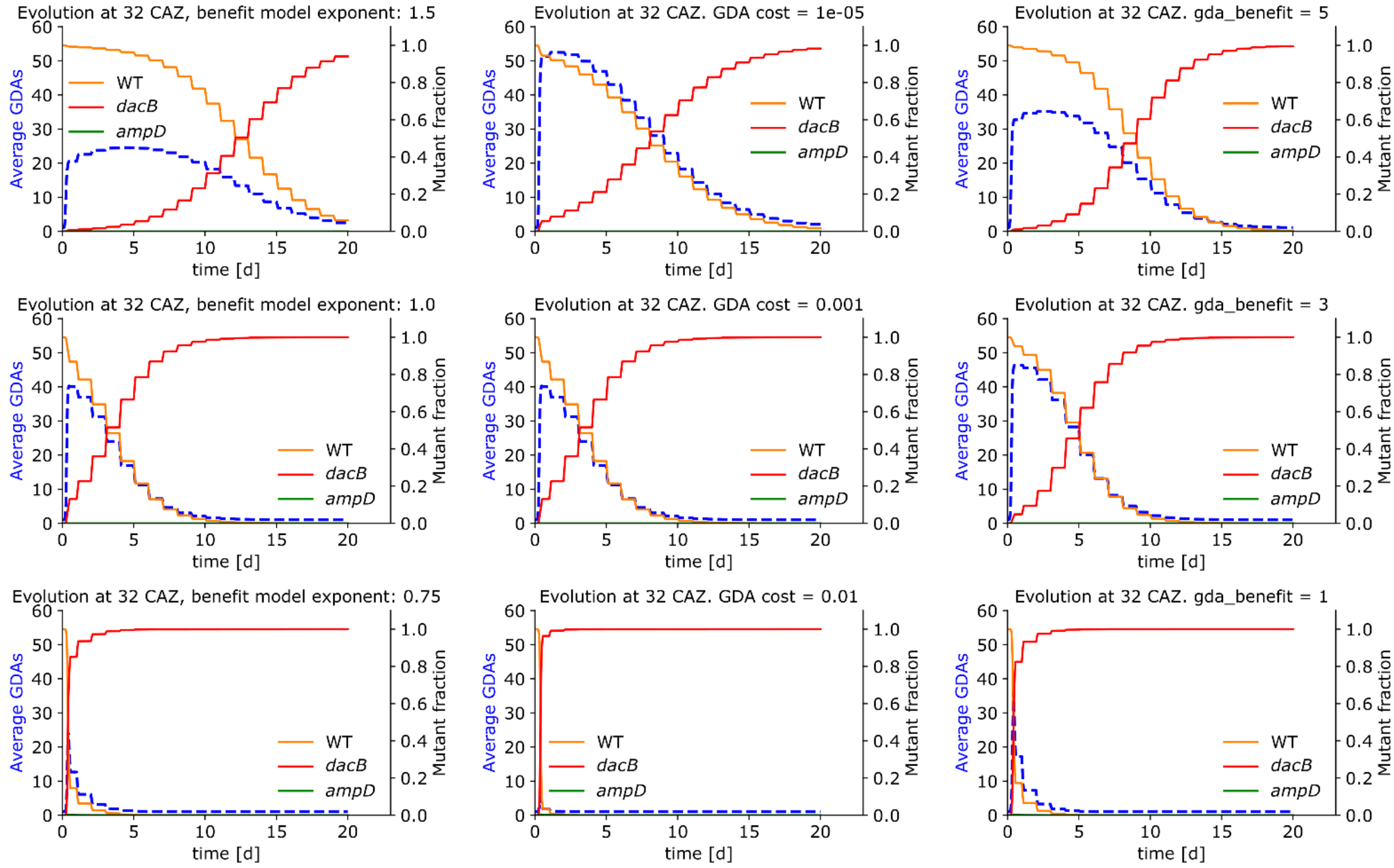

Figure S12. Influence of benefit function form and parameter values of cost and benefit on model predictions. Left column: changing the scaling of GDA benefit between synergistic (top), linear (center) and diminishing returns (bottom) by altering the exponent *a* in Eq. 3 in the main text changes the delay of mutant fixation. Center column: Decreasing the cost per GDA increases the delay of mutant fixation. Right column: Increasing benefit per GDA increases the delay of mutant fixation. The center row represents the values used in the main text.

Figure S13.

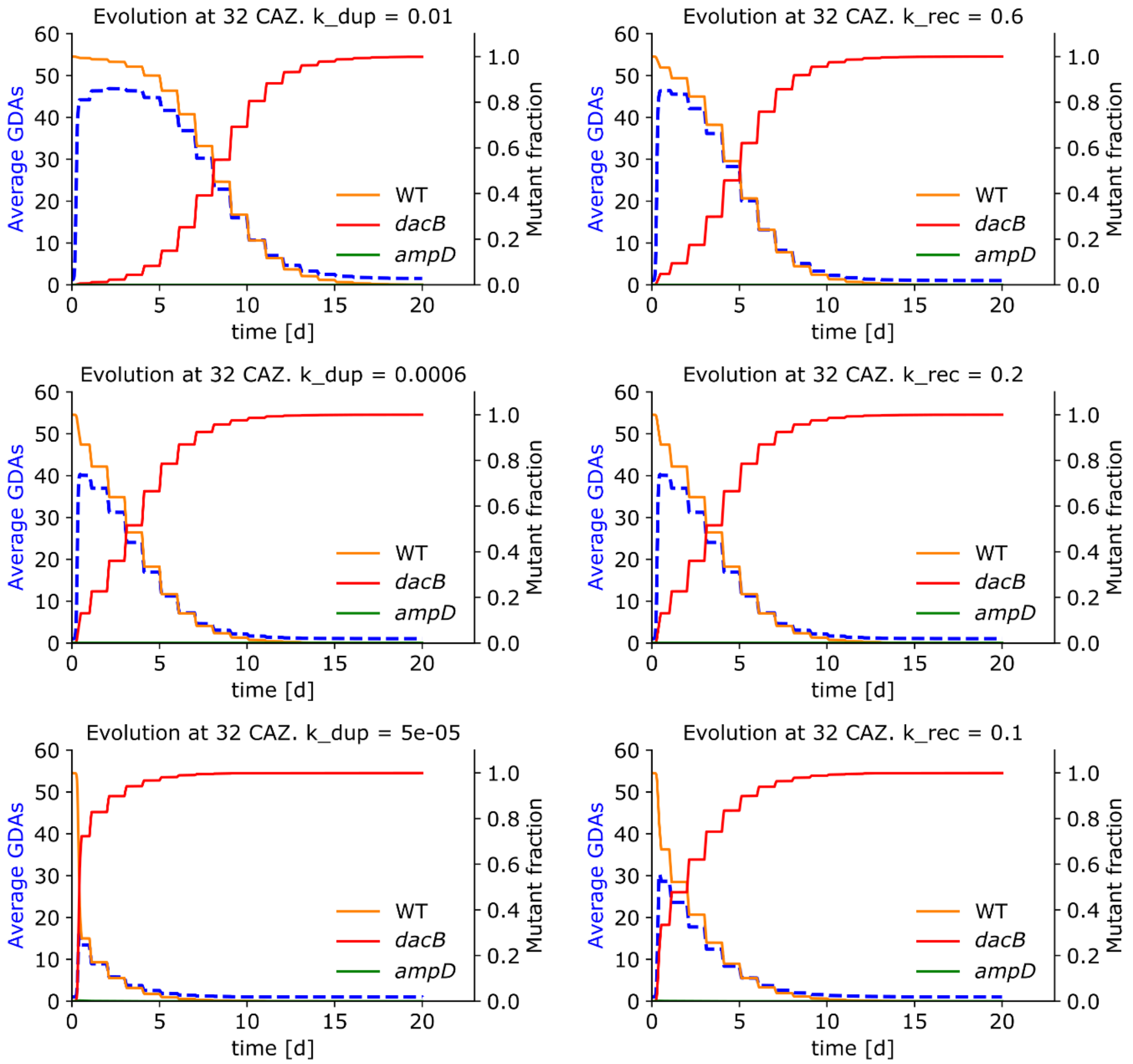

Figure S13. Influence of parameter values of duplication rate and recombination rate on model predictions. Left column: increasing the rate for the initial duplication rate results in higher average GDA copy numbers in the population and thereby delays the fixation of the mutant. Right column: Increasing the recombination rate results in higher average GDA copy number, but also higher chance for GDA collapse, only slightly increasing the delay of mutant fixation. The center row represents the values used in the main text.

Figure S14.

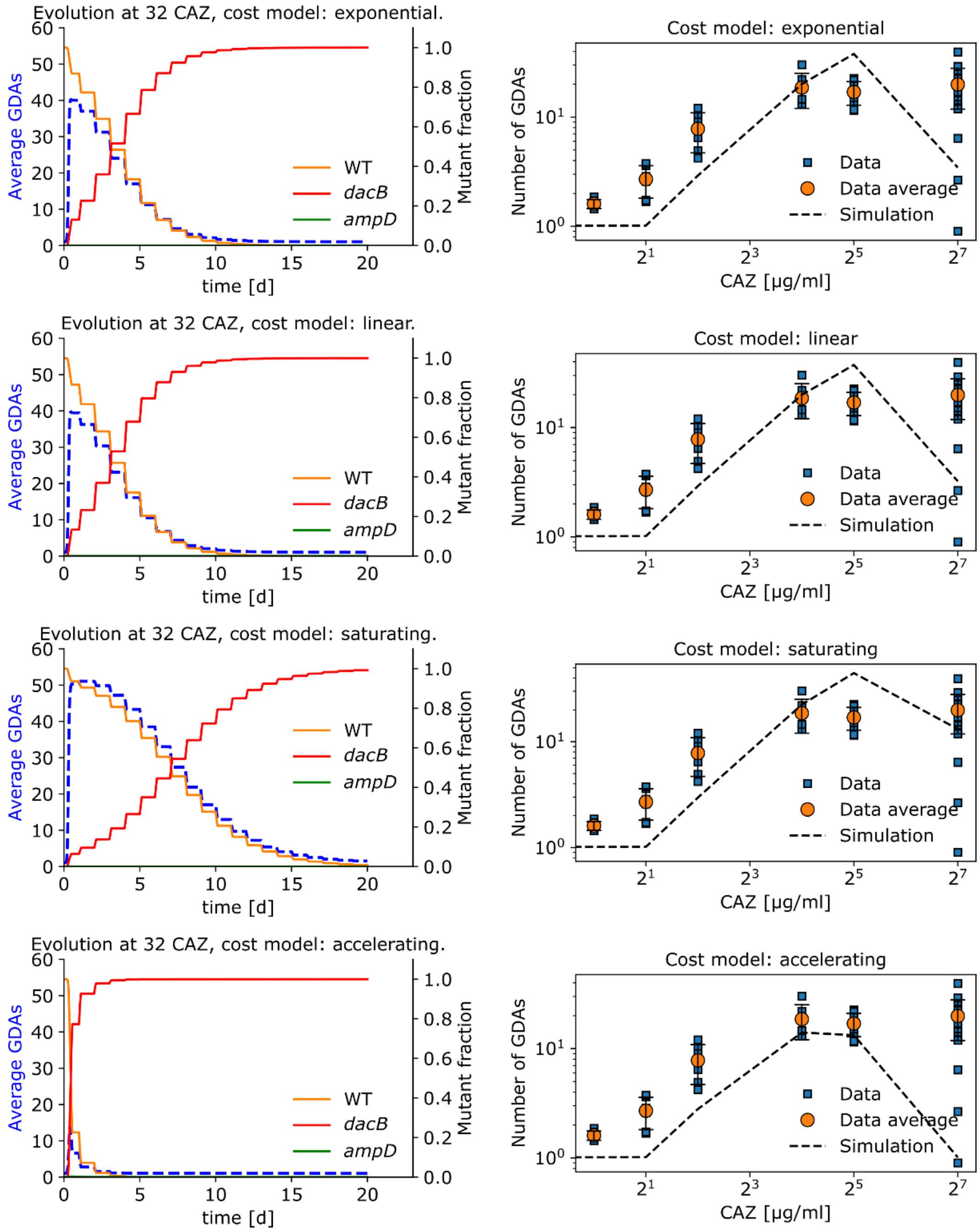

Figure S14. Exploring the influence of different fitness cost functions on model predictions (left column) and the model's ability to reproduce the average GDA copy number under different CAZ concentrations (right column; Fig. S11). The explored fitness cost functions are, from top to bottom: multiplicative/exponential ($(1 - gda\_cost)^{n-1}$; as in the main text), linear ($(1 - gda\_cost(n-1))$), saturating ($(1 - gda\_cost(n-1)^{0.5})$), and accelerating ($(1 - gda\_cost(n-1)^{1.5})$), where *n* is the GDA copy number. The lower bound of the fitness function is set to zero. The top row represents the cost-function used in the main text.

Supplementary References

1. Kupke, J. *et al.* Heteroresistance in *Enterobacter cloacae* complex caused by variation in transient gene amplification events. *npj Antimicrob. Resist.* **3**, 13 (2025) doi:10.1038/s44259-025-00082-7.

2. Pettersson, M. E., Andersson, D. I., Roth, J. R. & Berg, O. G. The amplification model for adaptive mutation: Simulations and analysis. *Genetics* **169**, 1105–1115 (2005).

3. Pal, A. & Andersson, D. I. Bacteria can compensate the fitness costs of amplified resistance genes via a bypass mechanism. *Nat. Commun.* **15**, 2333 (2024) doi:10.1038/s41467-024-46571-7.

4. Pettersson, M. E., Sun, S., Andersson, D. I. & Berg, O. G. Evolution of new gene functions: simulation and analysis of the amplification model. *Genetica* **135**, 309–324 (2009).

5. Sun, S., Berg, O. G., Roth, J. R. & Andersson, D. I. Contribution of gene amplification to evolution of increased antibiotic resistance in *Salmonella* Typhimurium. *Genetics* **182**, 1183–1195 (2009).

6. Nordholt, N., Kanaris, O., Schmidt, S. B. I. & Schreiber, F. Persistence against benzalkonium chloride promotes rapid evolution of tolerance during periodic disinfection. *Nat. Commun.* **12**, 6792 (2021) doi:10.1038/s41467-021-27019-8.

7. Kohlmann, R., Bähr, T. & Gatermann, S. G. Species-specific mutation rates for ampC derepression in *Enterobacterales* with chromosomally encoded inducible AmpC β-lactamase. *J. Antimicrob. Chemother.* **73**, 1530–1536 (2018).
